# Differential Expression of Host miRNAs During Ad14 and Ad14p1 Infection

**DOI:** 10.3390/v17060838

**Published:** 2025-06-11

**Authors:** Eric R. McIndoo, Ethan Wood, Gina Kuffel, Michael J. Zilliox, Jay R. Radke

**Affiliations:** 1 Research Section, Boise Veteran Affairs Medical Center and Idaho Veterans Research & Education Foundation, Boise, ID 83702, USA; eric.mcindoo@va.gov (E.R.M.); ethanwood2@isu.edu (E.W.); 2 Department of Chemistry, Northwest Nazarene University, Nampa, ID 83686, USA; 3 Department of Ophthalmology, Loyola University Chicago—Stritch School of Medicine, Maywood, IL 60153, USAmizilliox@luc.edu (M.J.Z.); 4 Department of Biological Sciences & Biomolecular Sciences Graduate Program, Boise State University, Boise, ID 83725, USA

**Keywords:** adenovirus, miRNA, acute lung injury, acute respiratory distress syndrome

## Abstract

Adenovirus is a frequent cause of mild, usually self-limited infections in infants and young children. Severe infections occur in immunocompromised patients but are rarely observed in healthy, immunocompetent adults. However, there have been outbreaks of infections with different adenoviral (Ad) types around the world that have resulted in acute lung injury (ALI) and acute respiratory distress syndrome (ARDS) in some of those infected. Ad14p1 is the predominant circulating strain of Ad14 worldwide that has caused ARDS. An explanation for the severity of illness caused by Ad14p1 infection in immunocompetent patients is unknown. Previously, we have shown that A549 cells infected with Ad14 repress macrophage pro-inflammatory responses, whereas cells infected with Ad14p1 fail to repress macrophages and instead can increase pro-inflammatory responses. Adenoviral infection has been shown to modulate host miRNA expression, and we hypothesized that differences in miRNA expression between Ad14- and Ad14p1-infected cells might explain the differential responses of macrophages to Ad14- and Ad14p1-infected cells. Analysis of host miRNA showed that 98 miRNAs are differentially expressed when infection reaches full cytopathic effect (CPE), the same point at which Ad14 and Ad14p1 CPE corpses induce differential inflammatory responses in macrophages. Only 10 of the miRNAs that were enriched in Ad14 CPE corpses were expressed at levels that are potentially biologically relevant. Pathway enrichment analysis showed that the differentially expressed miRNAs might explain the increased pathogenesis of Ad14p1 through strain-related loss of modulation of cytokine expression when compared with prototype Ad14. Overall, the data suggest a role for viral regulation of host miRNA expression in pathogenesis by regulating host inflammatory responses through the delivery of de-regulated miRNAs by viral CPE corpses to macrophages.

## 1. Introduction

Ambros and Ruvkun discovered the first microRNA (miRNA), *lin-4*, in *C. elegans* in 1993 [1,2]. miRNAs are small non-protein coding RNAs that can post-transcriptionally regulate gene expression, and they have been found in all animal models and show a high degree of conservation across species [3,4,5]. miRNAs are transcribed from DNA into primary miRNAs and processed into precursor miRNAs and finally mature miRNAs [6,7]. Mature miRNAs average 22–25 nucleotides in length and regulate gene expression by interacting with mRNAs primarily in the 3′ UTR, but they can also interact with the 5′ UTR mRNA coding sequence and gene promoters to suppress mRNA expression [6,8]. Mature miRNA associated with Argonaute proteins to form the RNA-induced silencing complex (RISC) [9]. In the RISC, nucleotides 2–8 of the miRNA (seed sequence) bind to complementary sequences of the target mRNA through Watson–Crick base pairing, resulting in the cleavage of the mRNA [10]. This provides the cell with a mechanism to fine-tune gene expression post-transcriptionally. While the majority of miRNAs reside intracellularly, miRNAs can be excreted by cells into the blood via exosomes or can be shed through other membrane vesicles such as apoptotic bodies, providing a way for communication with neighboring cells [11,12,13,14,15,16,17,18,19,20]. Secreted miRNAs can be biomarkers for many types of cancer, sepsis, nervous system disorders, traumatic brain injury, and infectious diseases [21,22,23].

miRNAs can play a role in regulating the immune response to viral infection and may be used as biomarkers to indicate severe infection [24]. Rhinovirus, respiratory syncytial virus, human metapneumovirus, SARS-CoV and SARS-CoV-2, influenza A, and adenovirus (Ad) infection result in differential miRNA expression in infected cells and in the blood of infected individuals [25,26,27,28,29,30,31,32,33,34,35]. miRNA expression in cells infected with respiratory viruses could regulate host inflammatory responses and could thereby determine the altered pathogenesis of emerging viral strains [36].

Respiratory Ad infection usually results in mild, self-limited infections in immunocompetent individuals. However, outbreaks of emergent strains of Ad have resulted in severe and sometimes fatal infections in otherwise healthy people [37]. Ad14p1 is one such emergent strain, which first emerged in the U.S. and subsequently throughout the world, resulting in acute lung injury (ALI) and acute respiratory distress syndrome (ARDS) [38,39,40,41,42,43,44]. Ad14 is a member of the B2 subgroup of adenovirus and uses desmoglein-2 as a receptor to infect respiratory epithelial cells [45]. Ad14 and Ad14p1 are 99.9% genetically identical [46]. We have shown that Syrian hamsters are fully permissive for Ad14/Ad14p1 infection and that hamster infection with Ad14p1 results in lung pathology that is consistent with ALI and early stages of ARDS [47]. In vitro infection of A549 cells with prototype Ad14 results in dying cells that repress human alveolar macrophage pro-inflammatory responses. In contrast, infection with the pathogenic Ad14p1 strain results in dying cells that enhance pro-inflammatory responses of human alveolar macrophages [48,49]. These results suggest that differential modulation of the host innate immune response to Ad14/Ad14p1 infections may determine whether infection results in resolution of viral pathogenesis or progression to acute lung injury and ARDS [49,50,51,52]. The objective of the current study was to determine whether Ad14p1 infection leads to differential miRNA expression that could explain the disparate inflammatory responses of alveolar macrophages to cells dying from Ad14 vs. Ad14p1 infection.

Here, we show that both Ad14 and Ad14p1 infection of A549 cells results in temporal changes in miRNA expression during the course of HAdV infection. Globally, both Ad14 and Ad14p1 have a similar effect of miRNA expression at early times after infection. However, the miRNA patterns are markedly different at late times after infection, when viral replication results in cell death. miRNA target and pathway enrichment analyses suggest that the differentially expressed miRNAs may account for virus strain-specific differences in immunopathogenesis. These data provide one explanation for the divergent inflammatory responses of alveolar macrophages to cells dying from Ad14 vs. Ad14p1 infection.

## 2. Materials and Methods

### 2.1. Cells and Viruses

A549 cells (CCL-185, ATCC, Manassas, VA, USA) were maintained in DMEM and grown at 37 °C and 5% CO_2_, as described previously [48,49]. A549 cells were validated by short tandem repeat markers (STR, ATCC) and monitored for mycoplasma contamination by PCR (ATCC). Ad14 deWit (VR-15) was obtained from ATCC and Ad14p1 (1986T) was obtained from the United States Naval Health Research Center, San Diego, CA, USA [46]. Viruses were propagated in A549 cells and plaque-titered in A549 cells. Stable κB-luciferase reporter 293 cells have been described [53].

### 2.2. Infection of A549 Cells and Isolation of Total Cellular RNA

A549 cells were infected in suspension with either Ad14 or Ad14p1 at an MOI of 10 pfu/cell for 1 hr at 37 °C, after which cells were plated and allowed to adhere until collected. Adherent and non-adherent cells were collected at 6, 12, 24, 36, and 48 hr post-infection. Total RNA was isolated using the miRNeasy kit (Qiagen, Germantown, MD, USA) with on-column DNase treatment. The total RNA in each sample was quantified using the Qubit 2.0 Fluorometer (Invitrogen, Carlsbad, CA, USA), and quality was measured using the RNA6000 nanochip on the Agilent 2100 Bioanalzyer (Agilent Technologies, Santa Clara, CA, USA). Samples with an RNA integrity number (RIN) greater than 8 were used for sequencing.

### 2.3. miRNA Library Preparation, Sequencing, and Initial Data Processing

Sequencing libraries were generated using the TruSeq Small RNA library prep kit (Illumina, San Diego, CA, USA). The libraries were size-selected using a 6% polyacrylamide gel and concentrated using ethanol precipitation. Purified libraries were normalized and pooled to create a double-stranded cDNA library ready for sequencing. The samples were sequenced on the Illumina MiSeq platform to render 50 base pair single-end reads. Adapter sequences were removed and low-quality reads were trimmed from raw sequencing reads using Cutadapt (v. 1.11), and the samples were demultiplexed.

### 2.4. Human miRNA Data Analysis

CLC Workbench 22 (Qiagen) was utilized for further data analysis. Quantify miRNA 1.3 was used to quantify miRNA counts, allowing for 2 additional upstream or downstream bases and 2 missing upstream or downstream bases with no mismatches. Reads were annotated with miRbase 22, which also included HAdV14 mivaRNA and a piRNA database 1.7.6 [54]. Results were grouped based on mature and seed sequences. Principal component analysis (PCA) was performed with PCA for RNA-seq 1.3 with the data grouped on seed sequence. A heatmap was created with Create Heat Map for RNA-seq 1.5 with the data grouped on seed sequence. Samples were clustered based on Euclidean distance and average linkage with 30 features.

### 2.5. miRNA Differential Expression Analysis

Differential Expression for RNA-Seq 2.7 was used, using the small RNA option on samples grouped on seed sequence. Normalization was performed with the trimmed mean of M values. Comparisons were performed between all groups using the Wald test. False discovery rate (FDR) *p*-values were determined by the Benjamini–Hochberg method. Maximum average of group RPKM values were determined using between groups testing for differential expression based on viral strain. Differential expression of miRNA was considered significant if the FDR was ≤0.05. Venn diagram for RNA-seq 0.2 was used to create the Venn diagrams of significant differentially expressed miRNAs.

### 2.6. Adenoviral RNA Differential Expression Analysis

Library preparation, sequencing, and processing of mRNA libraries have been previously described [54]. RNA-seq Analysis 2.6 from CLC Workbench 22 (Qiagen) was used to map reads to the adenovirus 14 genome (AY803294) with a mismatch cost of 2, insertion cost of 3, deletion cost of 3, and length/similarity fractions set to 0.8. Paired reads were counted as a single read. Differential Expression for RNA-seq 2.7 was used to determine differential gene expression between infected cells using the values determined from RNA-seq analysis. The Wald test was used to compare infection groups and mean expression defined as transcripts per million (TPM). False discovery rate (FDR) *p*-values were determined by the Benjamini–Hochberg method.

### 2.7. Bioinformatics Analysis

KEGG and GO analyses were performed with MirPath V.3 (7 May 2022) using Fisher’s exact test with a *p*-value threshold of 0.05 with FDR correction on the 10 enriched miRNA in Ad14 CPE corpses [55]. Dot plots were created using ggplot with the MirPath data. Heatmaps were downloaded from MiRPath. Mienturnet (http://userver.bio.uniroma1.it/apps/mienturnet/) was used on 11 May 2022 for target enrichment analysis [56]. TargetScan and miRTarBase were used for miRNA–target enrichment with a threshold of a minimum of 2 miRNA–target interactions and a threshold adjusted *p*-value of 1. Network analysis of the miRNA–targets was performed using miRTarBase, allowing for both strong and weak interactions. Functional enrichment was conducted using miRTarBase with the KEGG, REACTOME, and WikiPathways databases. The miRNA Target Filter and Network/My Pathways were generated through a Qiagen Ingenuity Pathway Analysis [57]. Briefly, Ad14 miRNAs were uploaded with expression data to IPA. Results were filtered by (1) cells/immune cells/macrophages; (2) pathways including cellular stress and injury, cytokine signaling, disease-specific pathways, and pathogen-influenced signaling; and (3) confidence to include experimentally observed and highly predicted results. Interaction of Ad14 miRNAs on signaling pathways were generated by overlaying the miRNA expression data on IPA canonical pathways.

### 2.8. NF-kB Luciferase Reporter Assay

A549 cells were infected with Ad14 or Ad14p1 as described above; then, adherent and non-adherent cells were collected at 6, 12, 24, 36, and 48 hr post-infection as follows. Non-adherent cells were collected by collecting media, and adherent cells were collected by incubation with EDTA for 3 min at 37 °C. Infected cells were then washed 3 times with PBS, counted, and resuspended in complete media to an equal cell concentration. Apoptosis was induced by incubating A549 cells with 1 μM staurosporine (Sigma-Aldrich, St. Louis, MO, USA) overnight at 37 °C, and necrosis was induced by incubating cells at 56 °C for 20 min. Apoptotic and necrotic corpses were then processed like Ad-infected cells. Plated 293-κB-luciferase cells were stimulated with 2 nM phorbol myristate acetate (PMA; Sigma) in the absence or presence of infected cells, viable, apoptotic, or necrotic A549 cells (at 10 A549 cells per 293-κB-luciferase cell) for 18 h, after which media and floating cells were removed and adherent cells were washed and then lysed for luciferase assays as performed previously [48]. Luciferase was measured with the Luciferase Assay Kit (Promega, Madison, WI, USA) and expressed as the fold induction of stimulated cells versus unstimulated control cells.

## 3. Results

### 3.1. Cellular miRNA Expression in A549 Cells During Ad14 or Ad14p1 Infection

To examine the effects of Ad14/Ad14p1 infection on cellular miRNAs, A549 cells were infected with either Ad14 or Ad14p1 at an MOI of 10 plaque-forming units (PFUs)/cell. Small RNA sequencing libraries were produced from total RNA extracted from cells at varying points post-infection that represent the full infectious cycle from early infection through to full cytopathic effect (CPE). As shown in Table 1, at 6 h post infection (hpi), there was little change in the percentage of small RNA reads that map to known human miRNAs, with less than one percent of the total reads mapping to Ad14/Ad14p1 viral miRNAs (mivaRNA). mivaRNAs are encoded in the VA RNA gene and are processed by Dicer, resulting in functional miRNAs [58]. Beginning at 12 hpi, the percent of small RNAs that map to cellular miRNAs dropped, as mivaRNA expression increased. At 24 hpi and later, approximately 38% of the reads mapped to cellular miRNAs, and ~20% of the reads mapped to mivaRNAs. This is consistent with previous observations in Ad2- and Ad3-infected tissue culture cells [25,26]. To ensure that cells were equally infected with Ad14 and Ad14p1, RNA-seq was performed at each time point, and reads were mapped to the Ad14 genome. An analysis of all gene expression showed that at 6 hpi, early (E) viral genes were the predominant genes expressed, and by 24 hpi, expression had shifted to late (L) viral genes (Figure S1). Differential expression of reads mapping to the early E1A gene at 6 and 12 hpi and to the late L2 gene at 24 and 36 hpi between Ad14- and Ad14p1-infected cells showed no difference in the expression of either E1A or L2 genes between Ad14- and Ad14p1-infected A549 cells (Table 2).

### 3.2. Ad14 and Ad14p1 De-Regulation of Cellular miRNA Expression

**Figure 1:**
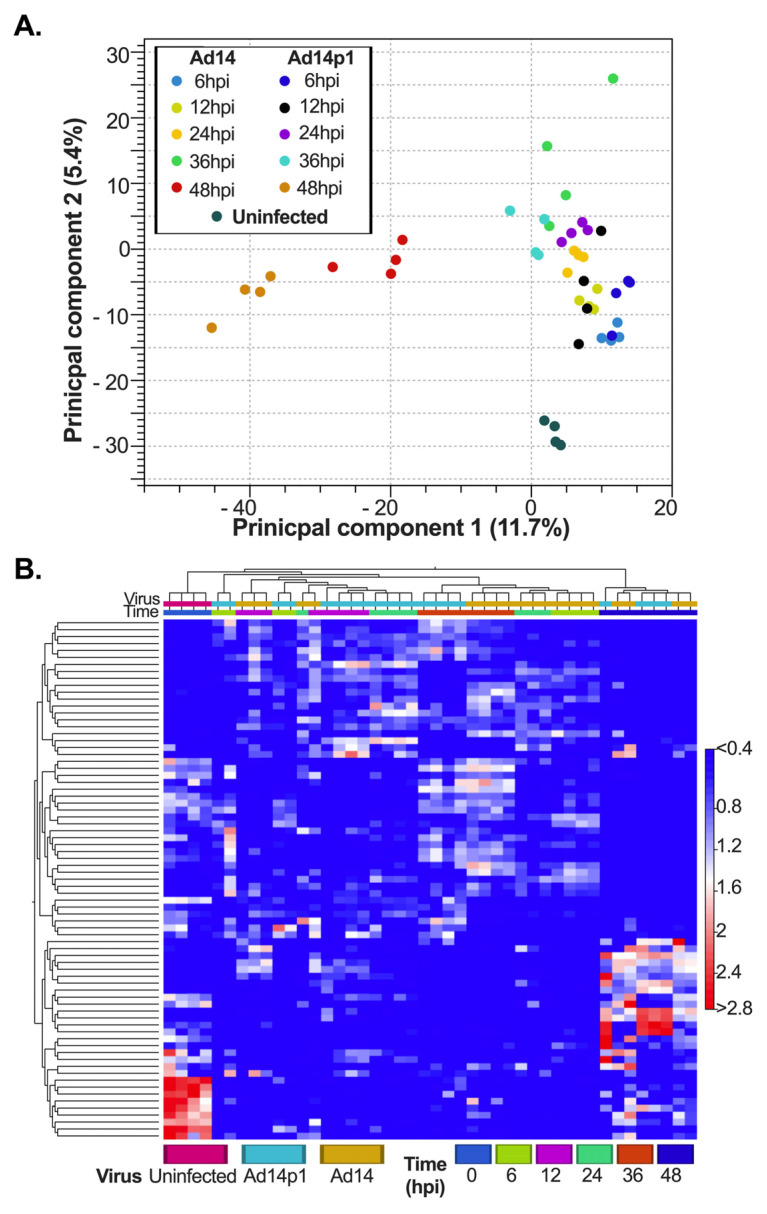
Unsupervised analysis of miRNA expression during Ad14 and Ad14p1 infection. A549 cells were infected with Ad14 or Ad14p1 at 10 PFUs per cell, and miRNAseq was performed from total RNA at the indicated times. (**A**) Principal component analysis plot of cellular miRNA expression during Ad14 and Ad14p1 infection. (**B**) Heatmap with Euclidean distance clustering of miRNA expression of the top 75 features.

While other studies have shown that Ad infection can de-regulate cellular miRNA expression, we wanted to determine whether there are differences in miRNA expression following infection with the non-pathogenic Ad14 compared with the pathogenic Ad14p1 strain [25,26,59]. To understand if Ad14 and Ad14p1 infection have the same effect on cellular miRNA expression, principal component analysis (PCA) and a heatmap with hierarchical clustering were used to identify patterns in the large complex datasets. Both Ad14 and Ad14p1 infection caused dysregulation of cellular miRNAs starting at 6 hpi (Figure 1A, light and dark blue dots) and to a similar degree at 12 hpi. At 24 hpi, de-regulation increased the principal component (PC) 2 direction. Overall, through 24 hpi, Ad14 and Ad14p1 de-regulation of miRNAs was similar. At 36 hpi, strain-dependent effects on cellular miRNA expression could be detected in the PC2 direction (Figure 1A, Ad14 in light green and Ad14p1 in teal), and the strain-related differences were even more apparent (a shift in the PC1 direction) at 48 hpi (Figure 1A, Ad14 in red and Ad14p1 in brown). At that point, infection had proceeded to the full CPE—virus-induced cytopathic effect—of all cells in the culture. The de-regulation seen at 48 hpi was distinct from the de-regulation seen at earlier time points. Overall infection resulted in both temporal- and viral strain-specific de-regulation of cellular miRNAs. Heatmap clustering of the top 75 expressed miRNAs (Figure 1B) showed both time-related and viral strain-specific differences between Ad14 and Ad14p1 infection on miRNA expression profiles, with the largest differences seen at 48 hpi.

### 3.3. Differential miRNA Expression During Ad14 and Ad14p1 Infection

Based on the unsupervised analysis, differential expression analysis was performed on miRNA from both Ad14 and Ad14p1 viral infections at each time point against uninfected cells and compared with each other. miRNAs that had a false discovery rate (FDR) adjusted *p*-value that was <0.05 were considered significant (Table 3 and Table S1). Infections with Ad14 or Ad14p1 both up-regulated and down-regulated miRNA expression compared with uninfected cells. At 6 hpi, both viruses had more up-regulated than down-regulated miRNA. The number of up-regulated miRNA fell at 12 hpi, and the ratio of up-regulated to down-regulated miRNA was nearly equal. From 24 hpi to 48 hpi, the number of total de-regulated miRNA increased in both infections, with some differences. In Ad14-infected cells, the total number of up-regulated and down-regulated miRNAs were very similar at all time points. In Ad14p1-infected cells, the number of de-regulated miRNAs decreased again at 36 hpi before a dramatic increase at 48 hpi that was far higher than in Ad14-infected cells, consistent with the PCA results.

**Figure 2:**
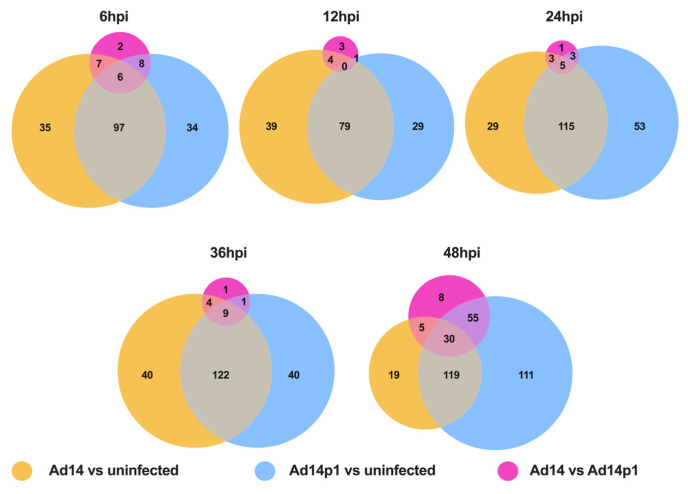
Ad14 and Ad14p1 de-regulation of miRNA expression. Venn diagrams of miRNA expression over time in Ad14- and Ad14p1-infected A549 cells. Comparisons are Ad14-infected vs. uninfected cells (yellow circles), Ad14p1-infected vs. uninfected cells (blue circles) and Ad14- vs. Ad14p1-infected cells (pink circles). Differential expression was determined by an FDR adjusted *p* value < 0.05 and absolute fold change >1.2 with N = 4.

The heatmap analysis showed that both Ad14 and Ad14p1 miRNA profiles between 6 and 36 hpi were similar, while at 48 hpi they were not. A Venn diagram analysis was used to understand the differences in the miRNA expression profiles in Ad14- vs. Ad14p1-infected cells. As seen in Figure 2, approximately 50% of the differentially expressed miRNA in Ad14- and Ad14p1-infected cells were shared (overlap of yellow [Ad14] and blue [Ad14p1] circles). Differential miRNA expression between Ad14- and Ad14p1-infected cells (Figure 2, pink circles) showed that, between 6 and 36 hpi, only 8–23 miRNA were differentially expressed in either strain. At 48 hpi, there were 98 differentially expressed miRNA in Ad14-infected cells compared with Ad14p1-infected cells. A total of 55 of the 98 miRNAs were differentially regulated in Ad14- vs. Ad14p1-infected cells and Ad14p1-infected vs. uninfected cells. Five were differentially regulated in Ad14- vs. Ad14p1-infected cells and Ad14-infected vs. uninfected cells. Thirty were differentially regulated in all comparisons, and eight were only differentially regulated in Ad14- vs. Ad14p1-infected cells.

### 3.4. Ad14 Differentially Expressed miRNA Target Cell Signalling Pathways

**Figure 3:**
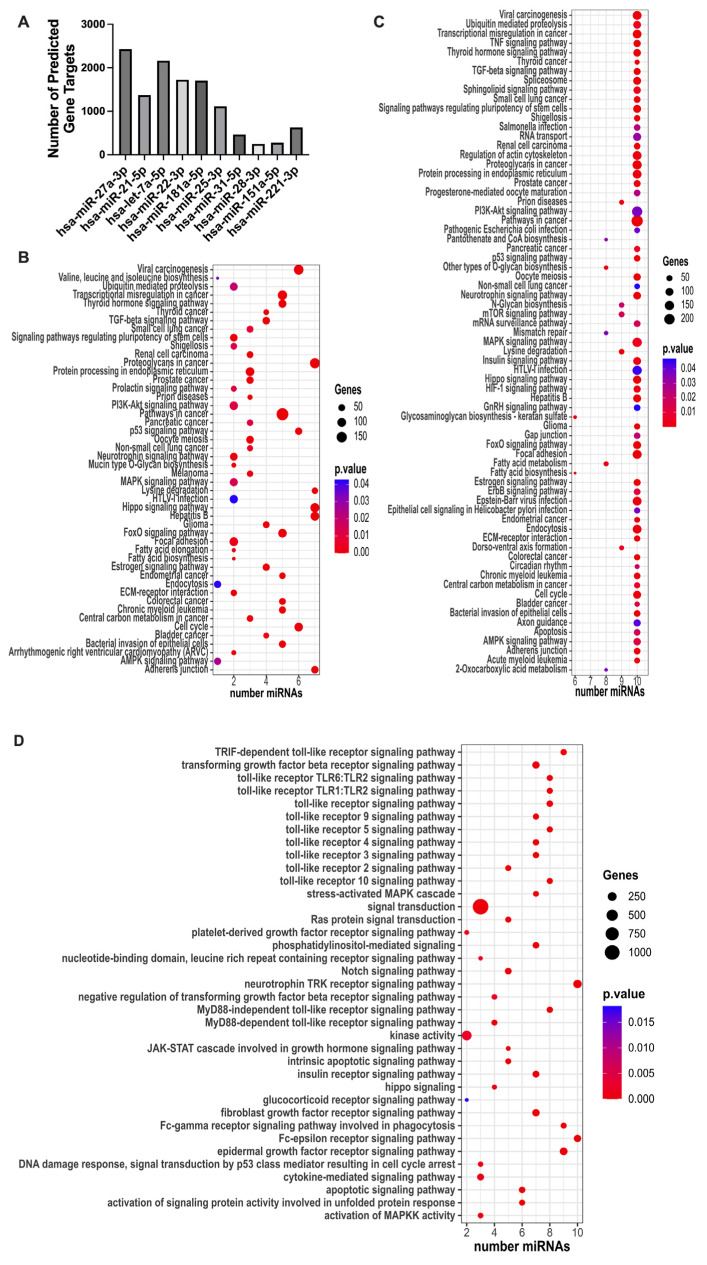
Bioinformatic analysis of Ad14 miRNA by mirPath V3. The 10 enriched miRNAs in Ad14 CPE corpses were uploaded to mirPath V3. (**A**) Total number of predicted target genes per miRNA. (**B**) Dot plot of the KEGG pathways union analysis. (**C**) Dot plot of the KEGG genes union analysis. (**D**) Dot plot of the signaling pathways identified by the GO categories union analysis.

**Figure 4:**
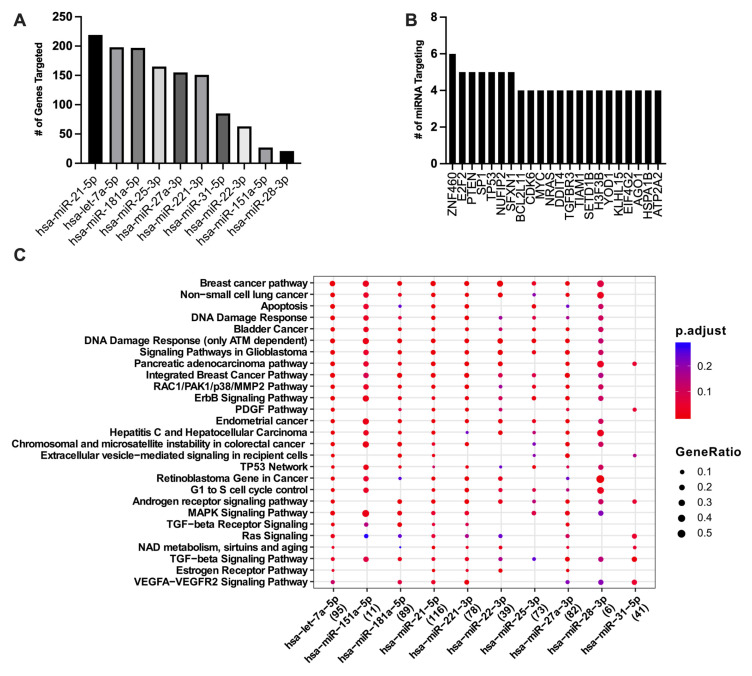
MIENTURNET target and functional enrichment of Ad14 miRNA. The 10 enriched miRNAs in Ad14 CPE corpses were uploaded to MIENTURNET. Target enrichment was performed with miRTarBase with a minimum of 2 miRNA–RNA interactions and an FDR threshold of 1. (**A**) Number of genes targeted per miRNA. (**B**) Number of miRNAs targeting each predicted gene for genes that were targeted by ≥4 miRNAs. (**C**) Dot plot of the WikiPathways functional enrichment. Number of genes targeted by each miRNA are shown in parentheses.

To begin to understand whether macrophage immunosuppression caused by Ad14 corpses was associated with their miRNA content, we focused on the 98 miRNAs that were differentially expressed in Ad14 vs. Ad14p1 corpses at 48 hpi (Table S1). To select for differentially expressed miRNAs in Ad14 CPE corpses compared with Ad14p1 CPE corpses that have the potential for a biological effect, a threshold was set for a mean expression of the miRNAs > 1000. This resulted in detecting 10 miRNAs that were enriched in Ad14 CPE corpses vs. Ad14p1 CPE corpses (Table 4). To understand the potential cellular effects of these miRNAs, mirPath v3 was used for KEGG and GO functional enrichment [55]. Using TarBase v7.0, the enriched Ad14 CPE miRNAs were shown to target between 249 and 2429 genes (Figure 3A and Table S2). The KEGG pathway enrichment analysis showed that multiple miRNAs target thyroid hormone, FOXO, p53, HIPPO, PI3K-Akt, and MAPK signaling pathways (Figure 3B,C and Figure S2). The GO analysis showed that the TLR, MAPK, Fc-epsilon receptor, epidermal growth factor, neurotropin TRK receptor, and TGF-*β* receptor signaling pathways are targeted by the enriched miRNAs (Figure 3D and Figure S2). The web-based tool MIENTURNET was used to further probe network-based analysis of the enriched miRNAs [56]. In MIENTURNET, target enrichment was performed with a minimum of two miRNA–mRNA interactions with the recommended default FDR threshold in TarBase v7.0. This resulted in 568 target genes for the 10 enriched Ad14 CPE miRNAs, of which 22 of the 568 genes are targeted by at least two or more of the enriched miRNAs (Figure 4A,B). Functional enrichment with Wikipathway, KEGG, and Reactome databases showed multiple signaling pathways to be targeted by most of the enriched miRNAs (Figure 4C and Figure S3).

### 3.5. Ad14 Expressed miRNA Targeting Cell Signaling Pathways in Macrophages

**Figure 5:**
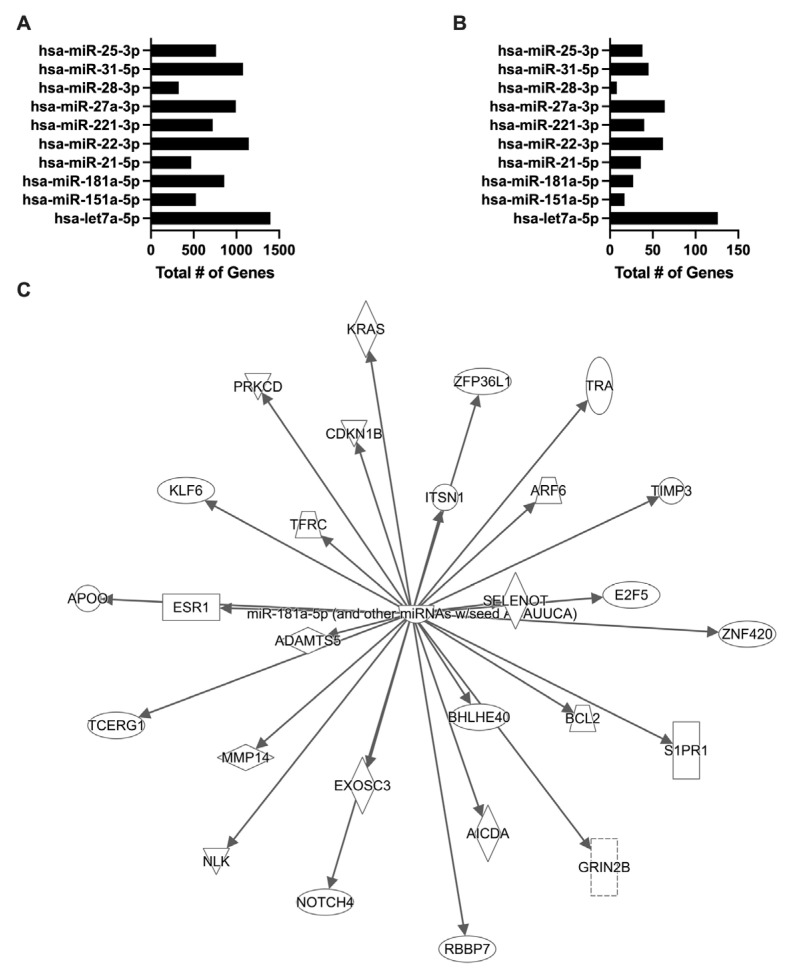
Ingenuity pathway analysis of Ad14-enriched miRNAs. (**A**,**B**). An IPA microRNA filter analysis was performed on the 10 Ad14-enriched miRNAs. The number of genes targeted by each miRNA is shown for unfiltered (**A**) and filtered (**B**) for cell type (macrophages), pathways (cellular stress and injury, cytokine signaling, disease-specific pathways, and pathogen-influenced signaling), and confidence level (experimental observed and highly predicted). (**C**) Visualization of the miR-181a-5p:mRNA target network from the filtered data.

Our previous studies have shown that Ad14 CPE corpses are capable of repressing macrophage inflammatory responses [49]. The bioinformatics analysis performed above indicated that the enriched miRNA in Ad14 CPE corpses can target many cellular signaling pathways. In order to learn more about how the identified miRNAs that are enriched in Ad14 CPE corpses, we used Qiagen’s Ingenuity Pathway Analysis (IPA) software to interrogate the functions of those miRNAs. IPA was used to determine which genes and signaling pathways in macrophages might be regulated by the Ad14 CPE-enriched miRNAs. Initial analysis showed that the 10 miRNAs are predicted to regulate 6233 mRNAs (Figure 5A). After filtering the IPA database for cell type (macrophages), pathways (cellular stress and injury, cytokine signaling, disease-specific pathways, and pathogen-influenced signaling), and confidence level (experimentally observed and highly predicted), 416 different mRNAs were predicted to be targeted for repression (Figure 5B and Table S3). Let7a-5p is predicted to interact with the most targets (Figure 5B). miR-181a-5p is predicted to interact with 28 mRNAs (Figure 5B,C). The network analysis showed that there are 463 interactions for the 10 enriched miRNAs and their targets (Figure S4).

### 3.6. Ad14 miRNA Targeted Signaling Pathways Regulate Inflammatory Responses and Acute Lung Injury

**Figure 6:**
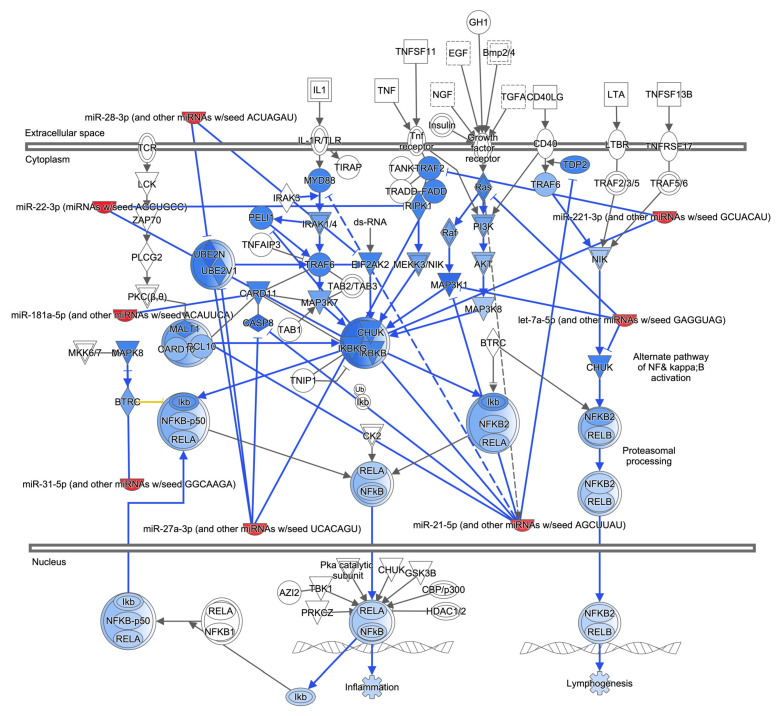
Effect of Ad14 miRNAs on the NF-κB signaling pathway. IPA was used to predict the effects of Ad14 miRNAs on proteins involved in activating NF-κB-dependent transcription. Lines ending with an arrowhead show the direction of activation, while lines ending with a dash show the direction of inhibition. Solid blue lines indicate validated repressive effects of miRNA–target interactions, while dashed blue lines are for predicted repressive effects of miRNA–target interactions. Red crescents are miRNAs, and the remaining shapes are proteins in the NF-κB pathway. Dark blue shapes are direct targets of the Ad14 miRNAs, while light blue shapes are predicted to have decreased activation based on repression of an upstream activator.

**Figure 7:**
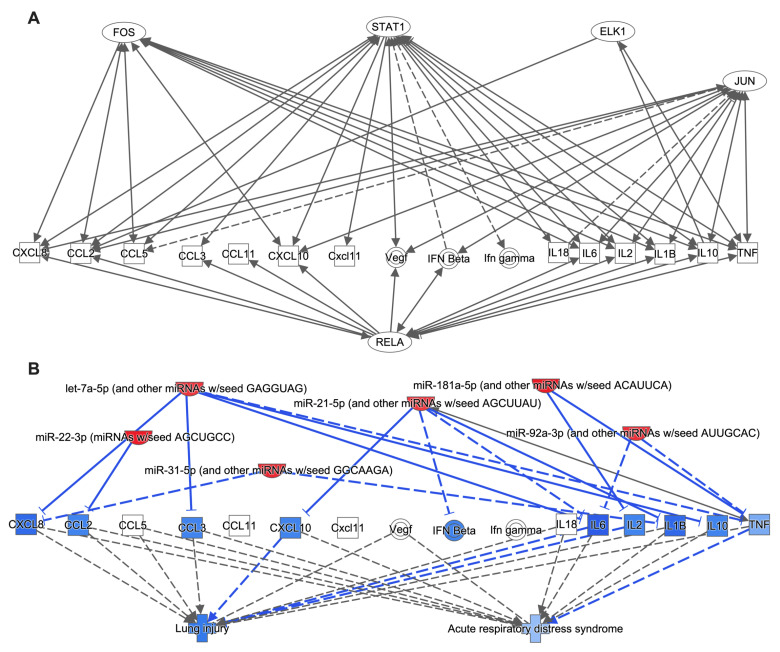
Bioinformatic analysis of regulation of chemokine and cytokine expression. (**A**,**B**). IPA was used to probe the relationships of transcription factors (**A**) and Ad14 miRNA (**B**) on the expression of chemokines and cytokines involved in ALI and ARDS. Lines ending with an arrowhead show the direction of activation, while lines ending with a dash show the direction of inhibition. Solid blue lines indicate validated repressive effects of miRNA–target interactions, while dashed blue lines are for predicted repressive effects of miRNA–target interactions.

We have shown that Ad14 CPE corpses repress NF-κB-dependent transcription [49]. Of the 416 mRNAs predicted to be targeted in signaling pathways in macrophages, IPA showed that 21 proteins are involved in NF-κB signaling pathways and regulated by eight of the Ad14 miRNAs (Figure 6). The targets for these miRNAs are upstream of NF-κB and collectively can repress NF-κB activation through numerous cellular receptors. Other signal transductions pathways, such as mitogen-activated protein kinase (p38 MAPK and ERK) pathways and the c-jun N-terminal kinase (JNK) pathways, also lead to activation of NF-κB-dependent transcription and can drive expression of pro-inflammatory cytokines through both NF-κB-dependent and -independent transcription. IPA analysis showed that the ERK signaling pathway is regulated by 9 of 10 Ad14 miRNAs (Figure S5), targeting proteins upstream and downstream of ERK, including transcription factors that ERK activates. Likewise, 9 of the 10 miRNAs target proteins in the p38 MAPK signaling pathway upstream and downstream of MAPK. MAPK11 (p38β) is directly targeted by miR-151-5p, miR-31-5p, and let7a-5p, while MAPK14 (p38α) is directly targeted by miR-22-3p (Figure S6). The JNK signaling pathway is targeted by 9 of the 10 Ad14 miRNAs, mainly upstream of MAP2K4/7 (Figure S7). ERK, p38, and JNK pathways lead to the activation of multiple transcription factors that drive the expression of cytokines and chemokines that can be involved in the inflammatory response, leading to acute lung injury (ALI). IPA showed that these transcription factors and NF-κB drive the expression of 15 cytokines and chemokines (Figure 7A). Further analysis revealed that six of Ad14 miRNAs can directly repress the activation of CXCL8, CXCL10, CCL2, CCL3, IL6, IL2, IL1β, IL10, and TNFα (Figure 7B). Repression of these chemokines and cytokines is predicted to decrease both ALI and ARDS.

### 3.7. Differential Expression of miRNA Correlates to Immunomodulatory Activity of Ad CPE Corpses

Our laboratory and others have shown that non-professional phagocytes such as 293 cells recognize viable, apoptotic, necrotic, and adenovirus CPE cells in the same manner as macrophages (professional phagocytes) with regards to regulating NF-κB-dependent transcription [48,49,53,60,61]. It is well established that apoptotic cells repress NF-κB-dependent transcription, and it is one of the ways that apoptotic cells are cleared from our bodies daily in the absence of an inflammatory response [62]. In contrast, necrotic cells otherwise fail to repress NF-κB-dependent transcription, and this accounts for one of the reasons necrotic cell death induces a pro-inflammatory response. Previously, we have shown that infection of A549 cells with adenoviruses that express sufficient amounts of E1B 19K results in CPE corpses that repress NF-κB-dependent transcription [48,49]. If our prediction is that these miRNAs are responsible for the differential inflammatory responses of macrophages exposed to Ad14 and Ad14p1 CPE corpses, then only Ad14-infected cells at 48 h post-infection should repress NF-κB-dependent gene expression. A549 cells were infected with Ad14 or Ad14p1 at an MOI of 10. Adherent and floating cells were collected at 6, 12, 24, 36, and 48 h post-infection. Viable, apoptotic corpses, necrotic corpses, or Ad14- or Ad14p1-infected cells were incubated with an NF-κB luciferase reporter cell line to test whether they were able to downmodulate NF-κB-dependent transcription. As expected, apoptotic A549 corpses repress NF-κB-dependent transcription while viable cells and necrotic corpses show little repression. Only Ad14-infected cells that have reached full CPE (48 h post-infection) repressed NF-κB-dependent luciferase activity. All cells infected with Ad14p1 failed to repress NF-κB-dependent luciferase activity. Previously, we have shown that Ad CPE corpses repression is not due to free virus killing the reporter cells, as evidenced by both formaldehyde-fixed and non-fixed Ad CPE corpses repressing NF-κB-dependent luciferase activity equally and by direct incubation of reporter cells with live virus at a ratio of 500 pfu/reporter cell resulting in increased NF-κB-dependent luciferase activity, which is inhibited by a neutralizing antibody [48]. Second, we have shown that repression of NF-κB-dependent luciferase activity requires direct interaction of the Ad CPE corpses with the reporter cell or macrophage, as when Ad CPE corpses are separated from the reporter cell or macrophages, soluble factors released from the Ad CPE corpses do not repress NF-κB-dependent luciferase activity or cytokine expression [48,49].

## 4. Discussion

The majority of adenovirus infections in healthy individuals are mild and self-limiting. However, with the increased surveillance for respiratory virus infections by PCR over the last 20 years, there has been increased detection of outbreaks of severe respiratory Ad infections in healthy patients, some even resulting in ALI/ARDS. Ad14p1 is a strain of Ad that can induce ALI/ARDS in otherwise healthy patients. We have shown that, although Ad14 and Ad14p1 are 99.9% genetically identical, they have different effects on the host inflammatory response. Ad14p1 infection of Syrian hamsters results in an ALI-like response, whereas Ad14-infected hamsters show minimal lung inflammation [47]. Ad14 CPE corpses repress macrophage pro-inflammatory cytokine expression, while Ad14p1 CPE corpses fail to repress these pro-inflammatory responses because of reduced expression of the Ad protein, E1B 20K [49]. Similar results were also observed with a deletion mutant of Ad5 that lacks E1B 19/20K expression [48]. The objective of these studies was to determine whether differential expression of cellular miRNA between Ad14 and Ad14p1 CPE corpses occurs and whether those differentially expressed miRNA control Ad14 CPE corpse immunomodulation of macrophage inflammatory responses.

Infection of lung cell lines with human Ad (Ad2 or Ad3) alters cellular miRNA expression [25,26]. Here, we infected human lung A549 cells with Ad14 and examined cellular miRNA expression using small RNA-seq. Our results (Table 1) show that infection with Ad14 or Ad14p1 results in a decrease in the total cellular miRNA reads over the course of infection, which is consistent with what has been observed [25]. The overall decrease in cellular miRNA reads at and beyond 12 hpi is associated with the processing of Ad14 VA RNA I into its mivaRNAs (Table 1). At 48 hpi, the total cellular miRNAs are further reduced in Ad14p1-infected cells compared with Ad14-infected cells. This is consistent with our previous findings that, at 48 hpi, there are more Ad14 mivaRNAs present in Ad14p1-infected cells than in Ad14-infected cells [54]. Zhao and colleagues reported that, during Ad2 infection, the majority of the significantly de-regulated miRNAs are repressed [25]. In contrast, our data (Table 3) show that, throughout Ad14 and Ad14p1 infection through complete cytopathic effect, roughly the same number of miRNAs are up-regulated or down-regulated. This difference is most likely explained by the fact that our analysis included more miRNAs, as we allowed a lower fold change and did not restrict miRNAs based on overall expression levels. Both Ad14 and Ad14p1 infection resulted in the same degree of miRNA de-regulation at all time points, except for 48 hpi, when Ad14p1 de-regulated nearly twice as many (315 vs. 173) cellular miRNAs (Table 3). A comparison of the miRNAs de-regulated showed that Ad14 and Ad14p1 de-regulated the same miRNAs at each time point (Figure 2) except at 48 hpi. The reason for this difference at 48 hpi is unclear. The only molecular difference we have identified so far between Ad14 and Ad14p1 infected cells is a marked reduction of Ad E1B 20K mRNA and protein in cells after Ad14p1 infection. Whether this difference in viral gene expression is the cause of the differential expression of cellular miRNA requires further study [49]. Expression differences in other viral genes between Ad14 and Ad14p1 would also have to be considered as possible factors in alterations in cellular miRNA expression. For example, E1A expression has been reported to down-regulate the expression of miR-27a, miR-520h, miR-7b, and miR-197 in breast cancer cell lines [63].

**Figure 8:**
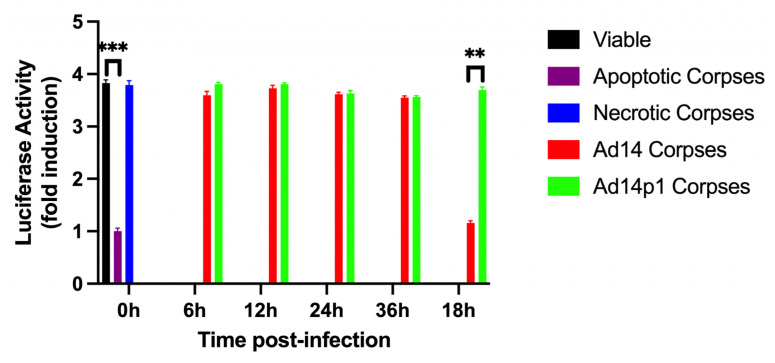
Effect of Ad14- and Ad14p1-infected A549 cells on NF-κB-dependent transcription in non-professional phagocytes. A549 cells were infected with 10 PFUs of Ad14 or Ad14p1 per cell. Adherent and floating cells were collected at 6, 12, 24, 36, and 48 h post-infection. Viable, apoptotic corpses, necrotic corpses, or infected cells were incubated with a 293 NF-κB luciferase reporter cell line at a ratio of 10 infected cells or viable cells per reporter cell in the presence of 2nM PMA. Values are the means ± standard error of the means. A one-way ANOVA test was performed, and *p*-values were determined by a post hoc Holm–Sidak test for infected cells compared with viable cells. *** *p* < 0.001, ** *p* = 0.0063.

Macrophages play a key role in removing cell corpses from sites of inflammation through efferocytosis. This process allows for the transfer of proteins, lipids, and nucleic acids from the dying cellular corpses to the macrophage [20,64,65]. It has been demonstrated that miRNA delivered from either exosomes or apoptotic corpses to macrophages can alter macrophage-mediated inflammatory responses through repression of NF-κB-dependent cytokine expression and the transition of M1 (pro-inflammatory) to M2 (anti-inflammatory) macrophages [17,20,66,67,68,69,70,71,72]. We have reported that Ad5 or Ad14 CPE corpses repress both NF-κB-dependent transcription induced by PMA and pro-inflammatory cytokine expression induced by either LPS/IFNγ or Ad viral particles in macrophages. In contrast, Ad CPE corpses dying as a result of infection with adenoviruses that lack sufficient expression of E1B 20K (such as Ad14p1) fail to repress those same cellular functions [48,49]. However, the mechanism through which Ad CPE corpses convey that immunomodulatory activity is unknown. GO, KEGG, and functional enrichment analysis (Figure 3 and Figure 4) revealed that the 10 miRNAs enriched in Ad14 CPE corpses target genes that are involved in many signal transduction pathways that induce pro-inflammatory cytokine expression. Restricting IPA analysis to known and strongly predicted miRNA–protein interactions in macrophage/monocytes revealed 8 of the 10 Ad14 miRNAs targeting 12 different proteins, with four of those being targeted by more than one miRNA in the NF-κB signaling pathway (Figure 6). The Ad14 miRNAs also target proteins that result in the inhibition of ERK, JNK, and p38 (Figures S4–S6) signal transduction pathways that activate FOS, JUN, ELK1, STAT1, and RELA transcription factors (Figure 7A), which drive pro-inflammatory cytokine expression [70,73,74,75,76,77,78,79,80,81]. Our data showing that only Ad14-infected cells at 48 h post-infection can repress NF-κB transcription (Figure 8) suggests the possibility that the enhanced expression of the Ad14 miRNA drives immuno-repression of macrophages exposed to Ad14 CPE corpses.

Prolonged acute inflammatory response during ALI can result in ARDS. Alveolar macrophages are long-lived resident macrophages that constitute >85% of the leukocytes in airspaces during ALI and, as such, are the first line of defense in the immune response to infection [82,83,84,85]. Many cytokines and chemokines produced by alveolar macrophages drive the progression of ALI/ARDS [86,87,88,89,90,91,92]. IPA analysis showed that six of the Ad14 miRNAs repress ten of the cytokines/chemokines that are regulated by transcription factors regulated by the Ad14 miRNAs, and nine of those cytokines/chemokines drive ALI/ARDS (Figure 7B). The possible relationship between reduced expression of the Ad14 miRNA during Ad14p1 infection and macrophage-mediated inflammatory responses is speculative. However, others have reported that many of the miRNA associated with Ad14 infection can regulate inflammatory responses, ALI/ARDS, and other lung inflammatory diseases. For example, increased expression of miR-181a-5p decreases NK-κB activation and alleviates inflammatory responses in COPD, whereas miR-181a-5p inhibition contributes to macrophage M1 polarization [93,94]. Increased expression of miR-27a-3p decreases expression of TNFα and IL6, while increased expression of let-7a-5p decreases expression of TNFα, IL6, and IL1β [78,95]. Studies by Das and colleagues have shown that miR-21-5p in apoptotic bodies engulfed by macrophages induces an M2-like macrophage phenotype associated with repressed NK-κB activation [19]. Other reports indicated that miR-21-5p can inhibit LPS-induced inflammation in ulcerative colitis [76,77,96]. MiR-22-3p has been reported to be involved in asthma, attenuating airway destruction and tissue damage and attenuating ALI [97]. Overall, it is increasingly apparent that miRNAs from extracellular vesicles (including apoptotic bodies) can regulate macrophage functions and might be targets for the design of therapeutic agents [67,72,98,99,100,101].

In this report, we examined the expression of cellular miRNA during infection with two pathogenically distinct strains of Ad14—Ad14 deWit (prototype Ad14) and the derivative outbreak strain, Ad14p1. Our data showed that, despite being over 99.9% genetically identical, Ad14 and Ad14p1 infections have distinct effects on expression of cellular miRNAs. The bioinformatic analysis of the miRNAs enriched in Ad14 CPE corpses help explain the previously observed repression of inflammatory cytokine expression in macrophages that have engulfed Ad14 CPE corpses. In contrast, the decreased expression of Ad14 miRNAs in Ad14p1 CPE corpses might explain the increased inflammatory responses following efferocytosis of Ad14p1 CPE corpses by macrophages. The roles of these miRNAs in modulating macrophage inflammatory responses to Ad14 and Ad14p1 CPE corpses are under investigation.

## Figures and Tables

**Table 1:** Summary of cellular miRNA and HAdV14/p1 mivaRNA expression.

	Mock	6 hpi	12 hpi	24 hpi	36 hpi	48 hpi
HAdV14						
Total Reads ^a^	2,915,019	3,408,959	3,391,732	3,387,175	3,330,665	2,821,104
miRBase (% ^b^)	1,794,862 (61.57%)	2,198,516 (64.49)	1,628,255 (48.01)	1,389,443 (41.02)	1,277,346 (38.35)	1,376,789 (48.8)
mivaRNA (% ^c^)	300 (0.013)	26,676 (0.78)	506,467 (14.93)	835,406 (24.63)	617,366 (18.54)	419,037 (14.85)
HAdV14p1						
Total Reads		2,803,990	3,267,888	4,990,604	4,160,615	3,310,553
miRBase (% ^b^)		1,755,693 (62.61)	1,533,510 (46.93)	1,738,608 (34.84)	1,507,889 (36.24)	1,242,118 (37.52)
mivaRNA (% ^c^)		14,835 (0.53)	258,305 (7.90)	1,032,127 (20.68)	891,497 (21.42)	640,156 (19.34)

^a^ Cumulative from 4 replicate infections and after discarded reads. ^b^ Percentage of total reads that mapped to miRbase V22. ^c^ Percentage of total reads that aligned to VA RNAs, which map predominantly to 5′ and 3′ mivaRNAs.

**Table 2:** Expression of E1A and L2 genes during Ad14 and Ad14p1 infection.

	E1A 6 hpi	E1A 12 hpi	L2 24 hpi	L2 36 hpi
HAdV14 Reads ^a^	75,426	16,324	209,916	180,365
HAdV14p1 Reads ^a^	74,330	28,386	218,934	227,102
Fold Change ^b^	−1.06	1.07	1.08	1.37
FDR ^c^	NS	NS	NS	NS

^a^ Average from 4 replicate infections expressed as TPM. ^b^ Fold change HAdV14p1 vs. HAdV14. ^c^ Benjamini–Hochberg test for false discovery rate.

**Table 3:** Number of differentially ^a^ expressed miRNA during infection.

	6 hpi	12 hpi	24 hpi	36 hpi	48 hpi
HAdV14 vs. Control					
Up	76	59	81	90	85
Down	69	63	71	85	88
Tota	145	122	152	175	173
HAdV14p1 vs. Control					
Up	74	52	98	94	179
Down	71	59	78	78	136
Total	145	111	176	172	315
HAdV14 vs. HAdV14p1					
Up	13	5	3	5	30
Down	10	3	9	10	68
Total	23	8	12	15	98

^a^ FDR adjusted *p* < 0.05, fold change > 1.2, filtered to remove low-expressing miRNAs.

**Table 4:** miRNAs enriched in Ad14 CPE corpses.

miRNA	Max Group Mean ^a^	Fold Increase ^b^	FDR *p*-Value ^c^
hsa-miR-27a-3p	71,095.25	1.348751	0.012899
hsa-miR-21-5p	67,880.5	1.31362	0.025758
hsa-let-7a-5p	27,083.75	1.353496	0.01777
hsa-miR-22-3p	19,373.75	1.558081	0.000147
hsa-miR-181a-5p	18,607.75	1.547176	0.000477
hsa-miR-25-3p	17,419.5	1.38644	0.010636
hsa-miR-31-5p	2736.25	1.450643	0.003482
hsa-miR-28-3p	2501	1.445523	0.005461
hsa-miR-151a-5p	1980.75	1.345793	0.025758
hsa-miR-221-3p	1947.5	1.414809	0.005475

^a^ Mean read counts from 4 different infections. ^b^ Fold increase in miRNA expression caused by Ad14 vs. Ad14p1 infection. ^c^ Benjamini–Hochberg test for false discovery rate.

## Data Availability

All RNA-seq data have been deposited to the Sequence Read Archive (SRA). Both the small RNA-seq and RNA-seq data from A549 infections are available from the bioproject PRJNA752359.

## Supplementary Materials

The following supporting information can be downloaded at: https://www.mdpi.com/article/10.3390/v17060838/s1, Figure S1: Mapping of total reads to Ad14 genome over time. Figure S2: Heatmaps of KEGG and GO analysis of Ad14 miRNAs. Figure S3: Dot plots of KEGG and Reactome functional enrichment of Ad14 miRNAs. Figure S4: IPA network analysis of Ad14 miRNA and predicted targets. Figure S5: Effect of Ad14 miRNAs on ERK signaling pathway. Figure S6: Effect of Ad14 miRNAs on the p38 MAPK signaling pathway. Figure S7: Effect of Ad14 miRNAs on the JNK signaling pathway. Table S1: Differentially expressed miRNA during Ad14 and Ad14p1 infection. Table S2: MirPath predicted targets for Ad14 miRNAs. Table S3: IPA predicted targets for Ad14 miRNAs.
