# Supplementary material for: Differential Expression of Host miRNAs During Ad14 and Ad14p1 Infection
Source: Viruses. 2025 Jun 11;17(6):838. doi: 10.3390/v17060838 (PMC12197781; doi:10.3390/v17060838)
Supplement: Supplementary file 1 [file viruses-17-00838-s001.zip › SI Table 1.pdf]

Ad14 v C 6HPI   Ad14 v C 12H

Identifier	Max group mean	Fold change	FDR p-value	Identifier	Max group mean	Fold change
hsa-miR-4490	7.75	21.29360261	0.002705967	hsa-miR-684	7.5	61.3765228
hsa-miR-3148	5.75	15.89349692	0.008746508	hsa-miR-105	6.5	53.3198831
hsa-miR-6799-5p	6.5	10.61360467	0.003258432	hsa-miR-375	7.25	20.5183667
hsa-miR-3125	7	8.107721216	0.002094532	hsa-miR-466	6.75	19.126241
hsa-miR-155-5p	3.75	6.222963807	0.039794513	hsa-miR-155	9.75	16.6090772
hsa-miR-493-3p	9.5	5.851009	0.000368338	hsa-miR-314	5	14.2587652
hsa-miR-5187-3p	23.5	4.31476471	7.48531E-08	hsa-miR-557	3.75	10.7832213
hsa-miR-369-3p	8.25	3.890615923	0.005193682	hsa-miR-427	10	9.58821453
hsa-miR-191-3p	6.25	3.879746152	0.013662149	hsa-miR-679	5	8.64474076
hsa-miR-493-5p	18	3.4719067	1.83169E-05	hsa-miR-520	12	7.90049093
hsa-miR-409-3p	96.25	3.179489594	1.6878E-19	hsa-miR-119	11.5	7.5711708
hsa-miR-152-5p	8.75	3.039654065	0.012599283	hsa-miR-888	15	7.54256363
hsa-miR-411-5p	141.5	3.03281733	6.12141E-22	hsa-miR-472	4	6.93765675
hsa-miR-1321	8.75	3.028666524	0.013774002	hsa-miR-312	5	6.1763477
hsa-miR-369-5p	6.25	2.963453556	0.037776995	hsa-miR-443	11.75	4.79623688
hsa-miR-889-3p	30.75	2.91809768	3.41365E-07	hsa-miR-125	11.25	4.19775511
hsa-miR-136-3p	51.5	2.896757557	2.9294E-08	hsa-miR-431	7.75	3.92820929
hsa-miR-4286	61.25	2.776624931	8.88844E-12	hsa-miR-493	5.75	3.83746357
hsa-miR-6131	23.5	2.764115943	2.67008E-05	hsa-miR-471	27.75	3.53560345
hsa-miR-1468-5p	8.5	2.718384147	0.017664112	hsa-miR-299	6	3.45994983
hsa-miR-375-3p	200.5	2.714217555	1.68418E-21	hsa-miR-681	5	3.33785533
hsa-miR-432-5p	7	2.677806016	0.035065231	hsa-miR-889	31	3.17982768
hsa-miR-92a-1-5p	22.25	2.621802171	6.29183E-05	hsa-miR-191	6.25	3.16891751
hsa-miR-300	199.75	2.603313446	7.44937E-21	hsa-miR-409	87.25	3.13616617
hsa-miR-487b-3p	9.25	2.545489719	0.018078389	hsa-miR-493	13.75	2.87346553
hsa-miR-410-3p	92.5	2.507672452	9.35178E-14	hsa-miR-410	96.25	2.83319402
hsa-miR-4451	16.5	2.461544585	0.002535639	hsa-miR-375	179.5	2.64757502
hsa-miR-485-3p	8.25	2.443592948	0.031811354	hsa-miR-411	113	2.63232618
hsa-miR-127-3p	636	2.421938742	2.88248E-33	hsa-miR-518	13	2.59192234
hsa-miR-143-3p	315.5	2.380045836	1.72869E-16	hsa-miR-9-5	93.5	2.57577452
hsa-miR-1269a	53.5	2.197482974	3.02699E-06	hsa-miR-711	13.5	2.57201761
hsa-miR-132-3p	96.5	2.186181066	3.18626E-09	hsa-miR-126	7.25	2.50333582
hsa-miR-3615	70.75	2.092940668	1.80019E-06	hsa-miR-445	15.5	2.47623496
hsa-miR-3184-5p	1213.25	2.065687	2.61892E-17	hsa-miR-432	10.5	2.45673931
hsa-miR-1299	33	1.973929192	0.001195274	hsa-miR-300	169.5	2.40199484
hsa-miR-671-3p	58.25	1.961748049	2.85195E-06	hsa-miR-428	46.5	2.29099851
hsa-miR-193a-5p	86.75	1.950688586	3.53654E-07	hsa-miR-127	525	2.21150546
hsa-miR-7706	92	1.931055942	8.42023E-07	hsa-miR-130	46.25	2.17975391
hsa-miR-532-5p	223.5	1.926116927	4.44536E-09	hsa-miR-548	17.25	2.15957845
hsa-miR-128-1-5p	39.5	1.892038537	0.001730097	hsa-miR-143	248	2.06501552
hsa-miR-100-3p	40.75	1.883549656	0.001105691	hsa-miR-312	23.5	1.95246413

hsa-miR-106b-3p	112.5	1.85173073	1.20901E-06	hsa-miR-770	84	1.92989073
hsa-miR-25-3p	27555.25	1.787278253	5.28415E-20	hsa-miR-132	76.5	1.90327849
hsa-miR-654-3p	95.75	1.733490638	2.50861E-06	hsa-miR-121	719	1.88903601
hsa-miR-192-5p	61738.25	1.706559858	4.00527E-17	hsa-miR-500	77.25	1.86303637
hsa-miR-9-5p	64.5	1.637345201	0.00080847	hsa-miR-25-	25360	1.83091898
hsa-miR-548q	24.25	1.632846092	0.048903728	hsa-miR-106	100.5	1.80984009
hsa-miR-1304-3p	37.25	1.617382112	0.007244354	hsa-miR-378	1447.75	1.79042718
hsa-miR-5009-5p	72.25	1.59727078	0.000368338	hsa-miR-193	72.5	1.78400698
hsa-miR-4499	24	1.589123898	0.048311271	hsa-miR-671	47.75	1.75201068
hsa-miR-181a-5p	24514	1.580481845	1.24625E-12	hsa-miR-318	927.5	1.75069192
hsa-miR-3927-3p	64.75	1.562260815	0.001236931	hsa-miR-654	84.25	1.66974451
hsa-miR-944	72.75	1.548236799	0.00265734	hsa-miR-361	50	1.62070306
hsa-miR-101-3p	502.5	1.54727767	3.06091E-06	hsa-miR-532	164	1.55134056
hsa-miR-10396a-5p	169	1.538163336	0.000989495	hsa-miR-148	836.5	1.46622488
hsa-miR-28-3p	5317.75	1.533747571	6.66035E-06	hsa-miR-797	133.25	1.42100513
hsa-miR-378a-3p	1358	1.518447654	2.03884E-09	hsa-miR-192	44690.25	1.38272713
hsa-miR-12135	625.25	1.492895713	9.21468E-08	hsa-miR-103	136.75	1.37853362
hsa-miR-151a-3p	5954	1.488367646	2.03884E-09	hsa-miR-151	4939.5	1.37669
hsa-miR-421	184.75	1.479462281	0.002042086	hsa-miR-183	440	-1.3450415
hsa-miR-182-5p	19159	1.476702473	2.94109E-09	hsa-miR-181	209.25	-1.3591246
hsa-miR-31-5p	6341.5	1.460748601	1.17504E-08	hsa-miR-210	270	-1.3773038
hsa-miR-221-3p	3722.75	1.428738976	1.32586E-07	hsa-miR-345	235.25	-1.3892262
hsa-miR-423-3p	1086.25	1.426122021	3.35868E-05	hsa-miR-130	509	-1.4004877
hsa-miR-30a-3p	656	1.405641409	6.66035E-06	hsa-miR-568	99.75	-1.4290227
hsa-miR-1180-3p	104	1.368430725	0.027792309	hsa-miR-27b	343.75	-1.4582561
hsa-miR-148a-3p	854.5	1.367934566	2.45375E-05	hsa-miR-151	2481.25	-1.559592
hsa-miR-128-3p	458	1.366291875	0.000105952	hsa-miR-100	3983	-1.5832476
hsa-miR-339-3p	76	1.352452022	0.046548104	hsa-miR-574	61.25	-1.6512258
hsa-miR-769-5p	515.75	1.351929526	0.000885321	hsa-miR-22-	31.75	-1.7027277
hsa-miR-126-5p	254.5	1.333697734	0.01452405	hsa-miR-125	43.75	-1.7109354
hsa-miR-27a-3p	99179.25	1.327761257	2.87395E-05	hsa-miR-29a	1045.75	-1.7114582
hsa-miR-34a-5p	451.5	1.30309192	0.001372993	hsa-miR-185	23.5	-1.7256726
hsa-miR-5579-3p	372	1.249778485	0.012583637	hsa-miR-326	27.5	-1.7541113
hsa-miR-320a-3p	767.25	1.242296311	0.047396446	hsa-miR-342	55.5	-1.7667971
hsa-miR-21-5p	140882	1.227083384	0.00481232	hsa-miR-374	111.75	-1.7884745
hsa-miR-103a-3p	2301.5	1.188810679	0.02897099	hsa-miR-24-	729.75	-1.7894499
hsa-miR-1297	8526.5	1.181463616	0.031108124	hsa-miR-450	67.25	-1.7955982
hsa-miR-183-5p	440	-1.250792956	0.009012233	hsa-miR-222	19.75	-1.8850796
hsa-miR-15a-5p	3259.5	-1.288993348	0.000368338	hsa-miR-15b	51.25	-1.898002
hsa-miR-29a-3p	1045.75	-1.293485218	0.000628184	hsa-miR-365	238.75	-1.9513444
hsa-miR-138-5p	657.5	-1.298573064	0.000885321	hsa-miR-103	16.25	-1.9565968
hsa-miR-210-3p	270	-1.309339562	0.026385908	hsa-miR-125	5732	-1.9699202
hsa-miR-1307-5p	509	-1.370227826	5.98506E-05	hsa-miR-23a	1803.5	-1.9812254
hsa-miR-151a-5p	2481.25	-1.377516901	3.57627E-06	hsa-miR-315	369	-2.0112734
hsa-miR-365a-3p	238.75	-1.390318785	0.008746508	hsa-miR-513	19	-2.0268608

hsa-miR-100-5p	3983	-1.39964092	7.5445E-07	hsa-let-7a-3	84.75	-2.0963686
hsa-let-7a-3p	84.75	-1.410727125	0.012599283	hsa-miR-33b	11.75	-2.181481
hsa-miR-4282	54.25	-1.467740483	0.011058595	hsa-miR-10a	13	-2.1957384
hsa-miR-652-3p	48.5	-1.467776135	0.030565873	hsa-miR-542	23.5	-2.2495162
hsa-miR-23a-3p	1803.5	-1.481230527	1.01948E-08	hsa-miR-365	12.25	-2.2729575
hsa-miR-450a-5p	26.25	-1.547024988	0.042193785	hsa-miR-452	42.25	-2.2931326
hsa-miR-2392	67	-1.547654599	0.037250532	hsa-miR-193	1137.5	-2.3159845
hsa-miR-452-5p	42.25	-1.550674063	0.008777735	hsa-let-7e-3	17.5	-2.4019492
hsa-miR-125a-5p	5732	-1.564632353	8.05385E-12	hsa-miR-193	25.75	-2.4030429
hsa-miR-345-5p	235.25	-1.574087773	9.55507E-07	hsa-miR-31-	94.5	-2.4462731
hsa-miR-22-5p	31.75	-1.600383413	0.024123207	hsa-miR-450	26.25	-2.462525
hsa-miR-15b-3p	51.25	-1.632061228	0.002042086	hsa-miR-331	319.25	-2.4841164
hsa-miR-24-3p	729.75	-1.634641919	9.08398E-10	hsa-miR-582	142.75	-2.5044941
hsa-miR-326	27.5	-1.654716355	0.037776995	hsa-miR-615	9.75	-2.5639462
hsa-miR-450b-5p	67.25	-1.735340524	0.000111875	hsa-miR-121	47.5	-2.6518458
hsa-miR-3155a	369	-1.781436892	3.1056E-13	hsa-miR-27a	59.75	-2.843397
hsa-miR-23a-5p	29.75	-1.781993372	0.006755219	hsa-miR-429	14	-3.0397877
hsa-miR-130b-5p	13.25	-1.847598379	0.046548104	hsa-miR-508	7.25	-3.2669327
hsa-miR-513a-5p	19	-1.877960391	0.018817553	hsa-miR-124	7.25	-3.2689853
hsa-miR-4650-3p	22.25	-1.880024287	0.005949352	hsa-miR-23a	29.75	-3.3568341
hsa-miR-193a-3p	1137.5	-1.959415616	1.06991E-14	hsa-let-7i-3p	17.5	-3.4008877
hsa-miR-141-3p	18	-1.961074044	0.023265012	hsa-miR-141	5.75	-3.4037493
hsa-miR-3978	20.75	-1.985347336	0.030939532	hsa-miR-887	8.5	-3.467956
hsa-miR-12133	47.5	-1.995823911	8.92639E-06	hsa-miR-324	9.75	-3.5403978
hsa-miR-342-3p	55.5	-2.028239726	3.86737E-05	hsa-miR-674	59	-3.7187136
hsa-miR-31-3p	94.5	-2.0315241	2.20116E-07	hsa-miR-990	149	-4.5840408
hsa-miR-27a-5p	59.75	-2.10724928	4.7134E-06	hsa-miR-578	10.5	-4.7034336
hsa-miR-331-3p	319.25	-2.143885835	7.07298E-11	hsa-miR-442	35.75	-4.7602787
hsa-miR-7-5p	20.75	-2.212172667	0.001312309	hsa-miR-342	7	-5.9616441
hsa-miR-193b-5p	25.75	-2.222033995	0.000441332	hsa-miR-124	6.25	-6.3608202
hsa-miR-4425	35.75	-2.243761671	0.006459381	hsa-miR-673	115.5	-10.116086
hsa-miR-18a-5p	24	-2.33183721	0.00050715	hsa-miR-445	4	-10.349613
hsa-miR-503-5p	11.25	-2.37390864	0.012599283	hsa-miR-675	4.5	-11.603304
hsa-miR-5089-5p	7.25	-2.391673864	0.048172566	hsa-miR-677	10.75	-11.694156
hsa-miR-3613-5p	8.5	-2.414682594	0.028536782	hsa-miR-394	22	-33.121885
hsa-miR-505-3p	8	-2.441713801	0.034670523	hsa-miR-607	7.5	-57.203548
hsa-miR-1296-5p	18.5	-2.489923644	0.000444053			
hsa-miR-6734-5p	11.5	-2.553245405	0.006248326			
hsa-miR-6808-5p	6.5	-2.55462373	0.048573728			
hsa-miR-324-3p	9.75	-2.586659617	0.017664112			
hsa-miR-33b-3p	11.75	-2.61139621	0.0047073			
hsa-miR-582-5p	142.75	-2.766641133	1.45449E-15			
hsa-miR-875-3p	9.25	-2.860994578	0.017903543			
hsa-miR-6728-5p	12.75	-2.990014611	0.000989495			
hsa-miR-6741-5p	59	-3.487797235	1.36775E-05			

hsa-let-7i-3p	17.5	-3.497254545	1.65808E-05
hsa-miR-6840-3p	4.75	-3.561358697	0.041186421
hsa-miR-890	5	-3.741905655	0.032218103
hsa-miR-342-5p	7	-3.833847407	0.009012233
hsa-miR-9900	149	-3.843798339	0.000155283
hsa-miR-6892-5p	5.25	-4.031068032	0.031108124
hsa-miR-4718	6.75	-4.260369825	0.006347337
hsa-miR-578	10.5	-4.523987514	0.000345771
hsa-miR-1246	6.25	-5.064507095	0.035591555
hsa-miR-6752-5p	4.5	-5.262785062	0.027033868
hsa-miR-34a-3p	4.75	-5.547756403	0.017664112
hsa-miR-6070	7.5	-6.790298146	0.001277869
hsa-miR-6774-3p	10.75	-6.868021193	0.000160203
hsa-miR-6737-5p	115.5	-9.070698429	3.24044E-35
hsa-miR-584-3p	3.5	-9.325112396	0.038500193
hsa-miR-3940-5p	22	-12.15629605	1.53367E-08

PI Ad14 v C 24HPI Ad14 v (

FDR p-value	Identifier	Max group mean	Fold change	FDR p-value	Identifier	Max group mean
0.02193832	hsa-miR-127	471.5	2.68519268	8.3477E-33	hsa-miR-518	34.75
0.0282903	hsa-miR-990	149	-4.7441554	4.9698E-31	hsa-miR-105	8.75
0.00312531	hsa-miR-25-	20627.5	1.98453754	1.092E-27	hsa-miR-684	5.75
0.00408364	hsa-miR-673	115.5	-16.537883	1.6771E-27	hsa-miR-312	51.25
0.00024718	hsa-miR-300	150.5	2.84743567	2.5809E-27	hsa-miR-466	3.75
0.01332408	hsa-miR-193	1137.5	-2.0636122	2.1617E-25	hsa-miR-155	6
0.03253087	hsa-miR-27b	343.75	-2.46075	2.1617E-25	hsa-miR-466	3.5
0.00036927	hsa-miR-375	139	2.74120474	9.4741E-25	hsa-miR-449	3
0.01226722	hsa-miR-411	96.5	3.0081375	1.8804E-22	hsa-miR-312	6
2.8359E-05	hsa-miR-378	1138.5	1.89089378	2.0398E-21	hsa-miR-548	12.75
2.8615E-05	hsa-miR-143	249.25	2.81123466	2.4728E-21	hsa-miR-428	144.25
1.9777E-06	hsa-miR-23a	1803.5	-1.8808798	3.7085E-21	hsa-miR-119	9.75
0.0282903	hsa-miR-428	75.5	5.01768765	3.5617E-19	hsa-miR-557	2.25
0.01855109	hsa-miR-132	95	3.13342666	7.3968E-18	hsa-miR-649	8.75
0.00010347	hsa-miR-410	72.75	2.86576492	2.613E-17	hsa-miR-472	3.25
0.00028925	hsa-miR-315	369	-2.0078678	4.5325E-17	hsa-miR-427	5.5
0.00873903	hsa-miR-9-5	74.75	2.75334893	9.8727E-17	hsa-miR-888	10.5
0.02832155	hsa-miR-331	319.25	-2.305022	9.8727E-17	hsa-miR-677	3
4.2753E-08	hsa-miR-106	105.25	2.53822638	1.6255E-16	hsa-miR-128	4
0.02267168	hsa-miR-27a	59.75	-7.4686987	8.6459E-15	hsa-miR-568	21.25
0.04511882	hsa-miR-674	59	-7.6231274	1.8044E-14	hsa-miR-127	16.5
7.2353E-06	hsa-miR-471	29.25	4.97320705	1.5504E-13	hsa-miR-449	7.75
0.04248943	hsa-miR-121	539.25	1.90016957	1.5504E-13	hsa-miR-124	20.5
2.9929E-14	hsa-miR-148	709.5	1.67279146	2.0712E-13	hsa-miR-125	11.75
0.00091123	hsa-miR-532	155	1.95680404	2.1819E-13	hsa-miR-668	20
7.292E-12	hsa-miR-318	728.75	1.84884124	4.4666E-13	hsa-miR-493	6.25
1.3149E-14	hsa-miR-125	5732	-1.9206437	2.5222E-12	hsa-miR-443	9.5
1.7555E-11	hsa-miR-582	142.75	-2.3360371	5.8167E-12	hsa-miR-672	4
0.00434081	hsa-miR-365	238.75	-1.8311022	4.7384E-11	hsa-miR-520	5.75
3.7739E-11	hsa-miR-889	29	3.98232137	7.2047E-11	hsa-miR-551	3.75
0.0028125	hsa-miR-500	68.75	2.20542802	7.2047E-11	hsa-miR-429	7
0.04729698	hsa-miR-31-	94.5	-2.3523397	7.2047E-11	hsa-miR-425	4.25
0.00878388	hsa-let-7a-3p	84.75	-2.4799905	7.2047E-11	hsa-miR-432	4.5
0.02645525	hsa-miR-151	4017.25	1.48922224	1.3904E-09	hsa-miR-889	27.75
6.9577E-09	hsa-miR-101	348.25	1.58501891	2.5936E-09	hsa-miR-120	8
1.6056E-05	hsa-miR-409	49.75	2.38852319	8.496E-09	hsa-miR-431	5.25
1.0218E-09	hsa-miR-119	15.25	13.3534772	9.0093E-09	hsa-miR-676	6.25
7.4213E-05	hsa-miR-181	17184.75	1.63388193	1.0265E-08	hsa-miR-106	130
0.0212453	hsa-miR-24-	729.75	-1.5222461	1.5302E-08	hsa-miR-410	74.75
2.3243E-05	hsa-miR-125	43.75	-3.1326036	4.3014E-08	hsa-miR-806	68.75
0.01113146	hsa-miR-442	35.75	-3.9751653	6.3813E-08	hsa-miR-447	4.25

2.4945E-05	hsa-miR-312	14.5	8.68625261	7.1767E-08	hsa-miR-369	4.25
0.00016257	hsa-miR-193	65.75	2.1922762	7.1767E-08	hsa-miR-9-5	76
6.3294E-06	hsa-miR-770	63	1.94239561	9.6117E-08	hsa-miR-126	5.75
0.00011091	hsa-miR-29a	1045.75	-1.4630058	1.2261E-07	hsa-miR-127	472.5
9.4469E-06	hsa-miR-181	209.25	-1.6543437	1.2495E-07	hsa-miR-300	137.25
5.8189E-05	hsa-miR-449	12.5	9.50833686	1.4852E-07	hsa-miR-268	10.75
2.5323E-06	hsa-miR-182	12365.5	1.41128583	1.6259E-07	hsa-miR-33a	5.75
0.00051715	hsa-miR-125	15	7.4574853	2.0868E-07	hsa-miR-143	224
0.01308371	hsa-miR-23a	29.75	-4.3317987	5.4095E-07	hsa-miR-315	16
2.1711E-05	hsa-miR-192	38121.75	1.59488073	1.3586E-06	hsa-miR-375	114.25
0.00118262	hsa-miR-126	196.25	1.51952294	1.6762E-06	hsa-miR-518	8.25
0.01642955	hsa-miR-315	20	2.81773851	1.8861E-06	hsa-miR-432	7.25
0.01225814	hsa-miR-128	39	2.16717259	3.4465E-06	hsa-miR-471	12.75
0.00738567	hsa-miR-601	29.25	-3.1304262	4.3454E-06	hsa-miR-7-5	31
0.00960823	hsa-miR-27a	71444.75	1.35342478	5.7987E-06	hsa-miR-689	15
0.03870302	hsa-miR-443	10.25	5.58786915	9.2315E-06	hsa-miR-569	6
0.01123233	hsa-miR-568	13	4.01832651	9.387E-06	hsa-miR-500	64.75
0.03870302	hsa-miR-394	22	-25.537202	9.9402E-06	hsa-miR-130	33
0.01650313	hsa-miR-28-	3228.25	1.3501866	1.1361E-05	hsa-miR-128	36.75
0.0423163	hsa-miR-130	34	2.13926752	1.8408E-05	hsa-miR-411	65
0.01428901	hsa-miR-797	100.75	-1.696463	1.8408E-05	hsa-miR-146	7.25
0.01543801	hsa-miR-427	8.5	10.7868522	2.3876E-05	hsa-miR-493	7.25
0.00392512	hsa-miR-222	19.75	-5.3199835	2.7209E-05	hsa-miR-770	65.75
0.02178237	hsa-miR-361	43.75	1.92164325	7.7359E-05	hsa-miR-409	39.75
0.00344382	hsa-miR-574	61.25	-1.9858122	8.0701E-05	hsa-miR-808	28
0.00093223	hsa-miR-548	16.25	2.69069953	0.00012589	hsa-miR-378	1133.25
0.00107339	hsa-miR-465	22.25	-3.0837119	0.00013111	hsa-miR-548	10.5
0.01440197	hsa-miR-493	11.5	3.21070924	0.00014031	hsa-miR-193	53.25
0.01788754	hsa-miR-124	10.75	3.51709756	0.00014189	hsa-miR-181	19021.5
0.01088839	hsa-miR-126	31.75	1.89333131	0.00016701	hsa-miR-25-	18406.25
1.6827E-05	hsa-miR-151	2481.25	-1.4229041	0.00018193	hsa-miR-126	27.75
0.02167141	hsa-miR-181	418.5	-1.3647202	0.00021333	hsa-miR-132	48
0.02660609	hsa-miR-7-5	29	1.97939437	0.00022083	hsa-miR-192	38400.5
0.0028125	hsa-miR-155	7	15.9460388	0.00025075	hsa-miR-449	15.75
0.00055591	hsa-miR-450	67.25	-1.829962	0.00027629	hsa-miR-361	34
5.1624E-06	hsa-miR-888	8	5.46026095	0.0002908	hsa-miR-532	116
0.00149706	hsa-miR-268	12.25	2.861235	0.00032789	hsa-miR-182	13029.25
0.03177486	hsa-miR-689	16.75	2.36179315	0.00033963	hsa-miR-148	612.25
0.001821	hsa-let-7i-3p	17.5	-3.2174871	0.00043147	hsa-miR-543	54.75
1.3935E-07	hsa-miR-33b	11.75	-7.5537075	0.00043966	hsa-miR-318	582
0.02233453	hsa-miR-34a	335.75	1.33246007	0.00055878	hsa-miR-769	362.75
2.6501E-07	hsa-miR-342	55.5	-1.7708359	0.0005743	hsa-miR-151	3849.5
3.6933E-08	hsa-miR-520	6.5	5.74671095	0.00057789	hsa-miR-500	33.75
2.7776E-09	hsa-miR-466	7.25	27.1050011	0.00069666	hsa-miR-126	183.25
0.01224399	hsa-miR-711	11	2.80066973	0.00069666	hsa-miR-27a	71444.75

2.7787E-06	hsa-miR-128	327.25	1.32536521	0.00078473	hsa-miR-101	311
0.02733844	hsa-let-7e-3p	17.5	-2.8486322	0.00110197	hsa-miR-340	235.75
0.01855109	hsa-miR-472	5.5	12.5790243	0.00111118	hsa-miR-186	792.25
0.00091123	hsa-miR-340	235.75	1.32876859	0.00152671	hsa-miR-103	1881
0.01638408	hsa-miR-186	792.25	1.27527134	0.00155986	hsa-miR-129	6967.25
4.3285E-05	hsa-miR-769	362.75	1.29815728	0.00187153	hsa-miR-15a	3259.5
2.3573E-11	hsa-miR-684	13.75	148.627998	0.00232929	hsa-miR-28-	241.25
0.00678019	hsa-miR-26a	9.25	-5.9616666	0.00321161	hsa-miR-130	509
0.0001227	hsa-miR-429	14	-2.964567	0.00339392	hsa-miR-22-	28627.5
4.3143E-08	hsa-miR-654	56.25	1.48094706	0.00368014	hsa-miR-181	418.5
0.00093223	hsa-miR-679	4.5	10.3359724	0.00373521	hsa-miR-345	235.25
2.0329E-12	hsa-miR-672	12.75	-3.1733819	0.00377322	hsa-miR-138	657.5
4.0256E-11	hsa-miR-578	10.5	-4.0285838	0.00385864	hsa-miR-568	99.75
0.01852622	hsa-miR-312	4.5	7.41036282	0.00418129	hsa-miR-15b	51.25
6.1316E-06	hsa-miR-100	3983	-1.3900736	0.00418129	hsa-miR-374	111.75
1.4959E-06	hsa-miR-105	10	107.793847	0.00489891	hsa-miR-151	2481.25
0.00118402	hsa-miR-365	12.25	-3.0513148	0.00515769	hsa-miR-125	80.75
0.01888326	hsa-miR-518	9.5	2.53494172	0.00516848	hsa-miR-335	56.75
0.01985943	hsa-miR-887	8.5	-5.4871954	0.00524647	hsa-miR-224	111
6.2044E-07	hsa-miR-685	8.75	-5.685826	0.00524647	hsa-miR-424	32
5.9824E-05	hsa-miR-15a	3259.5	-1.2294541	0.00543767	hsa-miR-24-	729.75
0.03739501	hsa-miR-450	26.25	-2.1971072	0.00543767	hsa-miR-181	209.25
0.03083442	hsa-miR-449	19.25	1.84753864	0.00612982	hsa-miR-318	42.25
0.00325623	hsa-miR-374	111.75	-1.3934326	0.00696708	hsa-miR-30c	47.25
0.00065725	hsa-miR-607	7.5	-15.017167	0.01022453	hsa-miR-29a	1045.75
0.00038088	hsa-miR-493	4.75	4.23717654	0.01263867	hsa-miR-797	100.75
0.00065724	hsa-miR-471	6.75	-7.9717754	0.01273355	hsa-miR-315	369
6.9577E-09	hsa-miR-185	23.5	-1.8777877	0.01405266	hsa-miR-365	238.75
0.00481579	hsa-miR-193	25.75	-1.934458	0.01416865	hsa-miR-190	28.25
0.03778346	hsa-miR-557	3.5	13.3735047	0.01467412	hsa-miR-23a	1803.5
1.9433E-20	hsa-miR-649	4.5	4.00742701	0.01470728	hsa-miR-450	29.75
0.03838287	hsa-miR-221	20	-2.3547426	0.01507472	hsa-miR-22-	31.75
0.02719952	hsa-miR-548	4.75	3.66308638	0.01570749	hsa-miR-125	5732
0.00025742	hsa-miR-423	740.5	1.31551843	0.01606412	hsa-miR-140	33.75
2.6174E-06	hsa-miR-513	19	-2.1100138	0.01617447	hsa-miR-360	63.75
0.02953091	hsa-miR-429	5	3.40001843	0.01656725	hsa-miR-574	61.25
	hsa-miR-106	1451.5	1.20527002	0.01784404	hsa-miR-193	25.75
	hsa-miR-335	176.25	-1.2928748	0.01877473	hsa-let-7a-3p	84.75
	hsa-miR-568	99.75	-1.371823	0.01877473	hsa-miR-193	1137.5
	hsa-miR-125	20.75	-1.8713685	0.01877473	hsa-miR-450	26.25
	hsa-miR-360	63.75	-1.4777521	0.02078328	hsa-miR-31-	94.5
	hsa-miR-130	94.25	1.31789964	0.02171496	hsa-miR-182	52.5
	hsa-miR-673	6.25	-5.2389297	0.02266008	hsa-miR-428	54.25
	hsa-miR-369	4.75	3.23481319	0.02459502	hsa-miR-331	319.25
	hsa-miR-454	15	1.80550277	0.02467714	hsa-miR-312	12.75

hsa-miR-316	25.75	-1.7183278	0.02624287	hsa-miR-465	44
hsa-miR-677	3	6.97138642	0.0271769	hsa-miR-487	12.25
hsa-miR-677	10.75	-71.017019	0.0287718	hsa-miR-452	42.25
hsa-miR-447	4.75	3.23427718	0.02902086	hsa-miR-582	142.75
hsa-miR-224	111	-1.3716802	0.0291866	hsa-let-7i-3p	17.5
hsa-miR-561	78	1.32421329	0.02920105	hsa-miR-715	22
hsa-miR-210	13.75	-2.1213476	0.02950606	hsa-miR-664	12
hsa-miR-15b	51.25	-1.5846487	0.03002462	hsa-miR-615	9.75
hsa-miR-100	21	-1.9574526	0.03002462	hsa-miR-542	23.5
hsa-miR-314	2.75	10.5919415	0.03131551	hsa-miR-10a	13
hsa-miR-29b	9	-2.7278176	0.03198622	hsa-miR-27b	343.75
hsa-miR-132	5.5	2.76203781	0.03332851	hsa-miR-26a	9.25
hsa-miR-465	44	-1.507867	0.03336528	hsa-miR-214	88.75
hsa-miR-673	11.5	-2.4365773	0.03456163	hsa-miR-125	43.75
hsa-miR-124	7.25	-3.2348344	0.03558611	hsa-miR-210	13.75
hsa-miR-183	440	-1.2829992	0.03684848	hsa-let-7e-3	17.5
hsa-miR-134	5	-10.100308	0.0370233	hsa-miR-222	19.75
hsa-miR-471	8.25	-2.8006826	0.03993485	hsa-miR-685	8.75
hsa-miR-889	5	-5.9448202	0.04173024	hsa-miR-315	8
hsa-miR-678	6.5	-3.4390856	0.04272732	hsa-miR-397	20.75
hsa-miR-34a	4.75	-9.6075645	0.04284866	hsa-miR-316	25.75
				hsa-miR-429	14
				hsa-miR-990	149
				hsa-miR-192	10.5
				hsa-miR-33b	11.75
				hsa-miR-442	35.75
				hsa-miR-601	29.25
				hsa-miR-465	22.25
				hsa-miR-27a	59.75
				hsa-miR-715	5.25
				hsa-miR-673	11.5
				hsa-miR-29b	9
				hsa-miR-342	7
				hsa-miR-607	7.5
				hsa-miR-677	10.75
				hsa-miR-674	59
				hsa-miR-34a	4.75
				hsa-miR-471	8.25
				hsa-miR-394	22
				hsa-miR-430	5.25
				hsa-miR-23a	29.75
				hsa-miR-471	6.75
				hsa-miR-672	12.75
				hsa-miR-673	115.5

C 36HPI Ad14 v C 48HPI Ad1

Fold change	FDR p-value	Identifier	Max group mean	Fold change	FDR p-value	Identifier
144.955825	1.467E-08	hsa-miR-518	93	251.482132	3.8804E-10	hsa-miR-127-3p
100.547201	0.00310806	hsa-miR-312	97	44.6022435	3.1356E-23	hsa-miR-192-5p
66.4768095	0.00834291	hsa-miR-684	5.25	39.8543634	0.03117988	hsa-miR-6737-5p
34.7878185	7.2106E-24	hsa-miR-807	5.25	39.1873894	0.03032373	hsa-miR-300
15.9226355	0.00506871	hsa-miR-681	21.5	21.9737687	0.00055992	hsa-miR-181a-5p
15.4362985	0.00025681	hsa-miR-520	32.75	21.698749	3.5467E-11	hsa-miR-3155a
15.0631664	0.00870042	hsa-miR-472	12	20.7112883	6.7885E-05	hsa-miR-143-3p
12.8338933	0.01173054	hsa-miR-428	396.75	20.0218463	5.0603E-79	hsa-miR-410-3p
11.1113501	0.00020104	hsa-miR-155	10	17.1972412	0.0002385	hsa-miR-182-5p
11.0299734	2.0152E-08	hsa-miR-124	5.75	16.0118773	0.00626485	hsa-miR-411-5p
10.6290386	3.1634E-55	hsa-miR-646	24	15.9688698	1.0041E-08	hsa-miR-5187-3p
9.7396477	1.1466E-06	hsa-miR-785	5	13.9561887	0.00932211	hsa-miR-409-3p
9.7335371	0.03406263	hsa-miR-312	11	13.4619261	6.7885E-05	hsa-miR-582-5p
8.75228409	4.2842E-06	hsa-miR-557	4.75	13.2777963	0.01118698	hsa-miR-4286
8.47655736	0.00847554	hsa-miR-126	22.75	13.0829622	2.0317E-08	hsa-miR-125a-5p
7.96368971	0.0004546	hsa-miR-429	23.5	11.9622904	1.4305E-09	hsa-miR-1307-5p
7.9244676	9.0981E-06	hsa-miR-127	41	11.4194897	8.0103E-15	hsa-miR-7706
7.84606117	0.01202846	hsa-miR-596	11.75	11.2380055	2.4853E-05	hsa-miR-27a-3p
7.4703974	0.00411339	hsa-miR-125	29.5	11.1207072	2.9912E-10	hsa-miR-25-3p
7.45476076	3.2755E-13	hsa-miR-432	16.25	10.7993579	1.6024E-06	hsa-miR-23a-3p
6.93684108	6.3406E-09	hsa-miR-888	19.75	10.0973519	4.8974E-08	hsa-miR-24-3p
6.73758493	3.1381E-05	hsa-miR-607	6.5	7.95342707	0.03117988	hsa-miR-889-3p
6.66090956	1.2827E-08	hsa-miR-121	24.5	7.40641653	2.0559E-08	hsa-miR-375-3p
6.63421445	8.2812E-07	hsa-miR-200	7.5	7.21584573	0.00117432	hsa-miR-193a-3p
6.34146669	1.2942E-11	hsa-miR-121	7.5	7.21485354	0.00164585	hsa-miR-345-5p
6.27975172	0.00025681	hsa-miR-124	29	7.18423189	5.3279E-10	hsa-miR-151a-5p
5.90170515	5.5823E-06	hsa-miR-472	16	7.02183105	0.0104206	hsa-miR-138-5p
5.82858566	0.0049853	hsa-miR-551	7.25	6.97927054	0.00199598	hsa-miR-151a-3p
5.78770387	0.00068868	hsa-miR-425	8.25	6.51131036	0.00094163	hsa-miR-532-5p
5.47995948	0.00718581	hsa-miR-368	3.75	6.48619648	0.03163791	hsa-miR-136-3p
5.38171264	0.00017279	hsa-miR-33a	23.25	6.42123528	2.5311E-07	hsa-miR-15a-5p
4.83269465	0.01525482	hsa-miR-427	6.5	6.2707947	0.00268433	hsa-miR-29a-3p
4.54952655	0.00605645	hsa-miR-302	5	6.195241	0.01043621	hsa-miR-365a-3p
4.34818403	1.2923E-13	hsa-miR-446	3.5	6.08096762	0.04697129	hsa-miR-100-3p
4.19205097	0.00025356	hsa-miR-492	14.5	5.51168277	1.5672E-05	hsa-miR-378a-3p
4.05765543	0.00517889	hsa-miR-426	6.75	5.33984333	0.00505255	hsa-miR-106b-3p
3.90228393	0.00153903	hsa-miR-483	18.5	5.22283559	4.8155E-06	hsa-miR-193a-5p
3.54114155	6.413E-25	hsa-miR-520	10	5.16969651	0.00050961	hsa-miR-769-5p
3.3495601	4.2437E-18	hsa-miR-568	21.5	5.06108964	2.495E-07	hsa-miR-671-3p
3.33938819	1.8112E-14	hsa-miR-366	8.5	5.02412015	0.01537905	hsa-miR-3615
3.30020474	0.01841553	hsa-miR-364	12.75	4.83090015	0.00015544	hsa-miR-7-5p

3.27602226	0.0418568	hsa-miR-548	8	4.65534784	0.0024787	hsa-miR-30a-3p
3.15139587	1.6103E-15	hsa-miR-569	4.75	4.6488103	0.04802981	hsa-miR-3940-5p
3.02584624	0.00997081	hsa-miR-478	13	4.5326925	0.00019029	hsa-miR-1269a
2.96789236	2.2207E-32	hsa-miR-450	14.75	4.43785691	2.642E-05	hsa-miR-148a-3p
2.96764623	3.6121E-19	hsa-miR-121	5.5	4.36873232	0.02032988	hsa-miR-9-5p
2.86923745	0.00095783	hsa-miR-7-5	79.25	4.12252341	2.756E-05	hsa-miR-5009-5p
2.67447199	0.03312876	hsa-miR-315	13.5	4.05983497	0.00022607	hsa-miR-101-3p
2.59697165	3.6725E-13	hsa-miR-569	15.25	4.03762414	0.00015544	hsa-miR-944
2.57799238	3.4675E-05	hsa-miR-268	22.25	3.95876921	2.1335E-06	hsa-miR-31-3p
2.57652276	1.0436E-18	hsa-miR-674	9.5	3.95346085	0.00326769	hsa-miR-331-3p
2.51640643	0.00578003	hsa-miR-119	5.75	3.87500964	0.01954272	hsa-miR-649
2.50456749	0.02053733	hsa-miR-130	7.5	3.82247447	0.01472019	hsa-let-7a-3p
2.48812577	0.00059966	hsa-miR-362	14	3.81196168	0.0008284	hsa-miR-6741-5p
2.42267517	1.743E-07	hsa-miR-315	34	3.67835196	1.1103E-06	hsa-miR-450b-5p
2.39549512	0.00086639	hsa-miR-443	8.75	3.64659806	0.0056062	hsa-miR-12135
2.39298209	0.02824865	hsa-miR-203	6	3.50774154	0.02569168	hsa-miR-128-3p
2.36997911	1.4708E-11	hsa-miR-518	11.5	3.46654504	0.00093578	hsa-miR-3181
2.3689883	1.5705E-07	hsa-miR-668	16	3.4080598	0.00015964	hsa-miR-676-3p
2.33775388	5.4163E-08	hsa-miR-106	178	3.36803097	8.0103E-15	hsa-miR-2392
2.32477115	7.2334E-09	hsa-miR-196	7.25	3.34242613	0.01648809	hsa-miR-21-5p
2.3217821	0.01789156	hsa-miR-126	9.5	3.34154468	0.00760677	hsa-miR-3927-3p
2.32129724	0.01944682	hsa-miR-451	13.75	3.27283056	0.00287964	hsa-miR-574-3p
2.30804408	1.0053E-09	hsa-miR-448	7	3.23014884	0.02721769	hsa-miR-22-5p
2.16698379	6.0981E-07	hsa-miR-361	25.25	3.19256012	4.8857E-06	hsa-miR-342-3p
2.16014257	9.9027E-05	hsa-miR-716	7.5	3.13036605	0.01400245	hsa-miR-132-3p
2.076147	1.3017E-19	hsa-miR-477	6	3.0983316	0.03077097	hsa-miR-6774-3p
2.00538865	0.03089478	hsa-miR-132	8	3.04304715	0.02457075	hsa-miR-15b-3p
1.98558104	7.314E-07	hsa-miR-548	8.25	2.89259165	0.01497872	hsa-miR-543
1.96465528	5.4803E-19	hsa-miR-673	15.75	2.72803098	0.00541852	hsa-miR-214-5p
1.9021763	2.8274E-16	hsa-miR-548	21.5	2.71787008	0.00135777	hsa-miR-654-3p
1.88819217	0.00027328	hsa-miR-711	13.5	2.62538752	0.00287964	hsa-miR-128-1-5p
1.80724998	2.5422E-05	hsa-miR-121	14.25	2.51499099	0.00718222	hsa-miR-424-3p
1.72940232	1.2961E-08	hsa-miR-808	12.25	2.49425849	0.01043621	hsa-miR-27a-5p
1.72536229	0.01847763	hsa-miR-132	96.75	2.46929432	7.251E-08	hsa-miR-10396a-5p
1.6830711	0.00109455	hsa-miR-808	42	2.2393916	0.00219648	hsa-miR-335-5p
1.66233207	2.8705E-05	hsa-miR-17-	23	2.10112833	0.0126492	hsa-miR-23a-5p
1.603921	5.4163E-08	hsa-miR-806	60.5	2.00403012	0.00360206	hsa-miR-1296-5p
1.58682887	2.641E-06	hsa-miR-9-5	65.75	1.85361519	0.00122212	hsa-miR-146b-3p
1.58092808	0.00062076	hsa-miR-450	106.25	1.72627988	0.00360206	hsa-miR-3978
1.4790424	0.00181171	hsa-miR-239	90.5	1.47014396	0.02286603	hsa-let-7e-3p
1.44961714	0.00102812	hsa-miR-186	1040.25	1.42495503	0.00292377	hsa-miR-452-5p
1.44604152	0.00010218	hsa-miR-148	766.5	1.37991254	0.00688031	hsa-miR-126-5p
1.39123832	0.04746683	hsa-miR-181	18607.75	1.35104133	0.01662154	hsa-miR-3148
1.38569489	0.01304828	hsa-miR-25-	17419.5	1.29082859	0.04939511	hsa-miR-4435
1.36318464	0.00086639	hsa-miR-23a	1803.5	-1.3343118	0.01539949	hsa-miR-6070

1.35288293	0.02112289	hsa-miR-22-	28627.5	-1.352864	0.0110381	hsa-miR-1910-5p
1.33865172	0.01482144	hsa-miR-31-	4277.25	-1.4061964	0.00505255	hsa-miR-5579-3p
1.29956953	0.00517731	hsa-miR-191	4219.5	-1.4281851	0.00287964	hsa-miR-4282
1.21262907	0.01048572	hsa-miR-941	184.25	-1.4314798	0.01574579	hsa-miR-146a-5p
-1.2227397	0.00517731	hsa-miR-21-	109325	-1.4922972	0.00015964	hsa-miR-450a-5p
-1.2361751	0.04746683	hsa-miR-360	63.75	-1.5142683	0.04939662	hsa-miR-30a-5p
-1.2800863	0.04687873	hsa-miR-561	78	-1.5583473	0.02231887	hsa-miR-7158-3p
-1.3044366	0.02132098	hsa-miR-122	9213	-1.5786022	6.8122E-05	hsa-miR-3613-5p
-1.3111023	0.00128283	hsa-miR-193	1137.5	-1.5788346	2.4853E-05	hsa-miR-28-5p
-1.3217516	0.01304828	hsa-miR-500	33.75	-1.5869535	0.03678329	hsa-miR-3125
-1.3669272	0.01138405	hsa-miR-143	125.5	-1.6059013	0.01925312	hsa-miR-6131
-1.3830731	0.00106192	hsa-miR-125	5732	-1.6085768	8.4893E-06	hsa-miR-129-5p
-1.4451281	0.02699224	hsa-miR-103	105.25	-1.6287843	0.00482583	hsa-miR-3691-3p
-1.4797463	0.02569176	hsa-miR-428	54.25	-1.6318674	0.03938519	hsa-miR-493-3p
-1.4804027	0.01961769	hsa-miR-99a	96	-1.6684781	0.00631401	hsa-miR-10a-5p
-1.4853525	0.00023216	hsa-miR-30a	25622.5	-1.7892492	1.0041E-08	hsa-miR-1307-3p
-1.4982633	0.00476292	hsa-miR-121	409	-1.8314564	6.1711E-06	hsa-miR-493-5p
-1.569815	0.00870042	hsa-miR-374	214	-1.8422983	6.8122E-05	hsa-miR-193b-5p
-1.6273369	0.00091951	hsa-miR-224	111	-1.8583637	0.00057394	hsa-miR-875-3p
-1.6364319	0.0190902	hsa-miR-557	288	-1.8664083	2.4731E-06	hsa-miR-130b-5p
-1.660586	1.1466E-06	hsa-miR-331	319.25	-1.8966138	1.9318E-05	hsa-miR-6728-5p
-1.7012723	0.00023096	hsa-miR-30a	450	-1.9867436	7.2032E-08	hsa-miR-4490
-1.7245086	0.01304828	hsa-miR-210	13.75	-2.0043828	0.0490135	hsa-let-7i-3p
-1.7305935	0.00517731	hsa-miR-134	33.75	-2.0262118	0.00135777	hsa-miR-660-5p
-1.7445449	1.153E-09	hsa-let-7a-3p	84.75	-2.0496708	0.00089325	hsa-miR-578
-1.7566834	1.4993E-05	hsa-miR-181	12.75	-2.1088626	0.04437787	hsa-miR-342-5p
-1.8010571	0.00012518	hsa-miR-452	42.25	-2.1480577	0.00326769	hsa-miR-486-5p
-1.8626245	1.5161E-05	hsa-miR-409	29.5	-2.1606378	0.00157386	hsa-miR-103a-3p
-1.8707468	0.00815986	hsa-miR-424	32	-2.2105802	0.00025738	hsa-miR-191-5p
-1.9083491	1.309E-12	hsa-miR-374	10	-2.2640415	0.03046791	hsa-miR-1304-3p
-1.9175481	0.0049853	hsa-miR-181	418.5	-2.3883464	4.5594E-09	hsa-miR-6799-5p
-1.9195194	0.00412943	hsa-miR-453	21	-2.3928826	0.00505255	hsa-miR-1257
-1.9355077	1.0934E-10	hsa-miR-443	10	-2.4093485	0.02404454	hsa-miR-9900
-2.0144054	0.00134816	hsa-miR-673	11.5	-2.4480242	0.0350585	hsa-miR-1827
-2.0308761	1.06E-05	hsa-miR-671	29	-2.4619128	0.00027177	hsa-miR-197-3p
-2.0312456	0.00039749	hsa-miR-194	12.75	-2.5652198	0.00528058	hsa-miR-421
-2.0959051	0.00351835	hsa-miR-30c	47.25	-2.6388605	0.00022607	hsa-miR-3184-5p
-2.1085603	2.4051E-07	hsa-miR-582	573.25	-2.6695341	8.5366E-16	hsa-miR-210-5p
-2.1313407	1.944E-13	hsa-miR-366	43.25	-2.8474188	1.5672E-05	hsa-miR-369-3p
-2.133774	0.00184656	hsa-miR-397	20.75	-2.8608991	0.00446305	hsa-miR-7846-3p
-2.1471463	3.1632E-07	hsa-miR-444	55	-3.053342	2.3858E-06	hsa-miR-155-5p
-2.1792092	4.9337E-05	hsa-let-7e-3p	17.5	-3.166803	0.00060424	hsa-miR-4425
-2.1813587	2.6452E-06	hsa-miR-193	25.75	-3.2280112	1.5672E-05	hsa-miR-5089-5p
-2.1851025	2.3711E-10	hsa-miR-125	80.75	-3.2519268	1.5928E-07	hsa-miR-4326
-2.2015646	0.03680409	hsa-miR-181	209.25	-3.2557729	5.8495E-17	hsa-miR-374a-5p

-2.2477213	3.268E-05	hsa-miR-680	6.5	-3.2709134	0.02992893	hsa-miR-106a-5p
-2.273509	0.03797896	hsa-miR-342	7	-3.5155756	0.02461962	hsa-miR-335-3p
-2.2988821	3.1906E-05	hsa-miR-125	20.75	-3.7515807	2.642E-05	hsa-miR-1299
-2.3162157	1.1415E-08	hsa-miR-125	43.75	-3.8887033	3.5055E-09	hsa-miR-589-3p
-2.3591578	0.00718581	hsa-miR-127	46.75	-4.0224347	5.592E-10	hsa-miR-4654
-2.5468153	0.00117075	hsa-miR-316	25.75	-4.0547672	4.6185E-06	hsa-miR-4718
-2.5771145	0.04198701	hsa-miR-128	20.25	-4.0563477	2.1525E-05	hsa-miR-5682
-2.5865731	0.03738485	hsa-miR-887	8.5	-4.2533738	0.00718222	hsa-miR-2355-3p
-2.5918396	0.00152572	hsa-miR-607	7.5	-4.3382924	0.01492882	hsa-miR-126-3p
-2.6043033	0.01304828	hsa-miR-365	12.25	-4.3506032	0.00023754	hsa-miR-6734-5p
-2.7068196	1.9917E-16	hsa-miR-430	5.25	-4.436326	0.02437539	hsa-miR-5197-3p
-2.7458661	0.04188479	hsa-miR-25-	13.75	-4.4476729	0.00015964	hsa-miR-136-5p
-2.7896585	1.5901E-09	hsa-miR-394	22	-4.6302375	7.9338E-05	hsa-miR-1249-3p
-2.8909038	1.474E-07	hsa-miR-469	4.25	-4.6546132	0.04891084	hsa-miR-542-3p
-2.9947433	0.0049853	hsa-miR-100	21	-4.7200132	5.3106E-06	hsa-miR-548u
-3.0136169	0.00153892	hsa-miR-674	59	-4.7260548	2.392E-10	hsa-miR-625-5p
-3.0293606	0.0016771	hsa-miR-465	22.25	-4.7781274	0.00013695	
-3.1643624	0.04594969	hsa-miR-612	9.25	-4.895971	0.0111364	
-3.1649071	0.0418568	hsa-miR-688	7.5	-5.1384022	0.00717162	
-3.2681179	0.00186285	hsa-miR-158	4.75	-5.1854137	0.02739551	
-3.6707281	3.1381E-05	hsa-miR-605	7.5	-5.1931986	0.01364133	
-3.7079009	0.00175211	hsa-miR-312	12.75	-5.5938283	0.00011774	
-3.7819707	5.6364E-16	hsa-miR-471	6.75	-5.6685894	0.00717162	
-4.0535315	0.00870042	hsa-miR-450	29.75	-5.9485678	1.9343E-08	
-4.5599465	0.00220602	hsa-miR-797	100.75	-6.2204826	1.9873E-14	
-4.9748645	3.8877E-08	hsa-miR-990	149	-6.4068338	1.5377E-25	
-5.0173402	1.5705E-07	hsa-miR-27b	343.75	-6.5116381	4.2925E-31	
-5.2888588	5.0661E-06	hsa-miR-214	88.75	-7.0670065	5.4654E-12	
-5.3824861	1.0042E-12	hsa-miR-23a	29.75	-9.5784605	1.319E-09	
-5.4761501	0.04434172	hsa-miR-29b	9	-9.6839981	0.00094727	
-6.4803468	0.00127007	hsa-miR-684	9.5	-10.211462	0.00064189	
-6.5759334	0.0049853	hsa-miR-601	29.25	-11.405132	1.0923E-09	
-7.2555598	0.01375883	hsa-miR-677	10.75	-11.53691	0.00025202	
-7.7633529	0.01087258	hsa-miR-33b	11.75	-12.595556	0.0001205	
-7.8812243	0.00233986	hsa-miR-442	35.75	-12.603224	3.5467E-11	
-8.3518806	5.4586E-15	hsa-miR-222	19.75	-13.151717	1.4569E-06	
-8.4562195	0.04928831	hsa-miR-715	5.25	-13.472712	0.0148829	
-8.5472576	0.00760846	hsa-miR-465	44	-14.138813	5.9209E-14	
-8.6306948	1.0464E-05	hsa-miR-672	12.75	-19.171276	0.00017983	
-9.3171749	0.0374062	hsa-miR-471	8.25	-21.282368	0.00403715	
-11.46161	4.4301E-08	hsa-miR-673	115.5	-21.598701	1.0754E-23	
-11.911404	0.01677273	hsa-miR-27a	59.75	-34.001932	8.0103E-15	
-13.092161	0.00080726					
-17.323387	7.7745E-26					

.4p1 v C 6HPI Ad14p1 v C 12HP

Max group mean	Fold change	FDR p-value	Identifier	Max group mean	Fold change
588.25	3.054929409	1.18459E-52	hsa-miR-10524-5p	5.25	48.18088227
56169.75	2.099939664	1.44066E-32	hsa-miR-6841-5p	4.75	43.69201414
115.5	-8.75822958	1.5653E-28	hsa-miR-375-5p	7.5	23.6377344
157.75	2.771765589	1.1418E-22	hsa-miR-4724-5p	5.5	10.53291573
21568.25	1.836453145	7.36195E-22	hsa-miR-155-5p	5.25	10.06436869
369	-2.104854288	5.14021E-19	hsa-miR-6799-5p	4	7.750974502
249.5	2.550402213	1.29897E-18	hsa-miR-7846-3p	3.5	6.786497169
79.75	2.928905485	5.67447E-18	hsa-miR-520f-3p	8.5	6.267494634
16352.75	1.701601349	8.2441E-17	hsa-miR-888-5p	10.5	5.901395702
93.75	2.715419171	9.09511E-17	hsa-miR-493-3p	7.75	5.714225717
28.25	6.987413203	8.72755E-14	hsa-miR-1197	7.5	5.535452904
61.25	2.736039686	1.87535E-13	hsa-miR-649	6.5	5.201515674
142.75	-2.690101075	2.70941E-13	hsa-miR-4431	10.5	4.778946472
48.5	2.968638337	1.52202E-12	hsa-miR-200a-5p	4	4.311523854
5732	-1.568961286	7.60003E-12	hsa-miR-1292-5p	5.75	4.261202841
509	-1.695906949	1.26997E-11	hsa-miR-889-3p	32.5	3.809385563
82.5	2.336591878	1.36153E-10	hsa-miR-5187-3p	16.75	3.714663167
84268.25	1.517333352	1.36153E-10	hsa-miR-152-5p	8.5	3.538366921
17346.75	1.509703989	2.78364E-10	hsa-miR-323b-3p	5.25	3.515156882
1803.5	-1.543134529	2.80411E-10	hsa-miR-676-3p	7.25	3.340330724
729.75	-1.643676083	1.25547E-09	hsa-miR-409-3p	74.25	2.976843356
27.25	3.489861598	2.71634E-09	hsa-miR-668-5p	13.25	2.964177489
107.75	1.972132727	3.0299E-09	hsa-miR-1253	7	2.930499354
1137.5	-1.687505979	5.84466E-09	hsa-miR-411-5p	99.75	2.645894723
235.25	-1.746235954	8.14767E-09	hsa-miR-127-3p	545.25	2.61641719
2481.25	-1.480353686	9.10858E-09	hsa-miR-9-5p	82.5	2.541351517
657.5	-1.531830525	1.89475E-08	hsa-miR-410-3p	77.5	2.526715314
4310.5	1.452584931	2.27473E-08	hsa-miR-5683	9.75	2.525168382
166	1.892402554	2.70057E-08	hsa-miR-493-5p	10.25	2.394865607
37.75	2.866776825	1.14031E-07	hsa-miR-4286	43	2.36248534
3259.5	-1.43474329	1.14031E-07	hsa-miR-375-3p	134	2.206118119
1045.75	-1.469103663	1.14031E-07	hsa-miR-5009-5p	80.25	2.195025458
238.75	-1.870641557	1.47379E-07	hsa-miR-128-1-5p	37.5	2.183078867
40.5	2.568408109	2.19412E-07	hsa-miR-7706	80.5	2.083535991
964	1.446297937	2.19412E-07	hsa-miR-106b-3p	97.5	2.000450434
86.75	1.943049065	2.83496E-07	hsa-miR-136-3p	26.75	1.859073197
65.75	2.000726613	2.98548E-07	hsa-miR-4714-5p	13	1.855238323
435.75	1.535186429	6.95813E-07	hsa-miR-671-3p	45	1.836003645
45.75	2.080184698	7.44258E-07	hsa-miR-300	121	1.833451672
54	2.180987488	8.44694E-07	hsa-miR-192-5p	50585.75	1.794831144
20.75	-6.03582443	1.64355E-06	hsa-miR-1285-3p	37.75	1.749551554

496.75	1.440238129	1.7107E-06	hsa-miR-378a-3p	1236.5	1.711691901
22	-19.23528517	2.14588E-06	hsa-miR-1304-3p	31.5	1.655151706
41.5	2.270425632	2.33955E-06	hsa-miR-193a-5p	58.5	1.606996015
655.75	1.411544156	3.39786E-06	hsa-miR-12135	531	1.586779549
56.25	1.928686862	4.27254E-06	hsa-miR-654-3p	69.5	1.529412904
60.75	1.813070181	4.62705E-06	hsa-miR-143-3p	171	1.503249496
371	1.540245881	5.50223E-06	hsa-miR-181a-5p	17741	1.421533842
64.25	1.854318407	9.15249E-06	hsa-miR-182-5p	14467	1.411548043
94.5	-1.902606136	9.69868E-06	hsa-miR-151a-3p	4446	1.396606483
319.25	-1.70611757	1.32569E-05	hsa-miR-3184-5p	671	1.382368804
9.5	7.881583668	2.25164E-05	hsa-miR-7974	116	1.368007819
84.75	-1.773864984	4.74478E-05	hsa-miR-138-5p	657.5	-1.375758231
59	-3.336407084	6.7033E-05	hsa-miR-196a-5p	128	-1.418778586
67.25	-1.827758958	0.000104586	hsa-miR-151a-5p	2481.25	-1.442020793
422	1.363129771	0.000108543	hsa-miR-28-5p	241.25	-1.484718457
327.25	-1.390200542	0.000198605	hsa-miR-5682	99.75	-1.500378318
42.25	-2.198980991	0.000198605	hsa-miR-125a-5p	5732	-1.524561848
9	4.609942673	0.000208007	hsa-miR-22-3p	28627.5	-1.578717343
67	-2.143179493	0.000212321	hsa-miR-210-3p	270	-1.586107041
110775	1.290856687	0.000230607	hsa-miR-574-3p	61.25	-1.688464273
50	1.629450175	0.000720767	hsa-miR-3155a	369	-1.702468175
61.25	-1.922283235	0.000829117	hsa-miR-374a-5p	111.75	-1.75047078
31.75	-2.126611047	0.000914985	hsa-miR-224-5p	111	-1.799233523
55.5	-1.865404774	0.000951298	hsa-miR-365a-3p	238.75	-1.803143383
54	1.656957664	0.00102281	hsa-miR-29a-3p	1045.75	-1.822781522
10.75	-13.46127055	0.001046694	hsa-miR-23a-3p	1803.5	-1.833418116
51.25	-1.758124373	0.001222624	hsa-let-7a-3p	84.75	-1.835025369
64.5	1.551148756	0.001449729	hsa-miR-140-5p	33.75	-1.841263031
88.75	-1.668474411	0.001552424	hsa-miR-1307-5p	509	-1.844188772
62.25	1.522131806	0.00158729	hsa-miR-345-5p	235.25	-1.873886957
30.5	1.952646206	0.001626843	hsa-miR-450a-5p	26.25	-1.962383749
32	-1.969239262	0.001663863	hsa-miR-24-3p	729.75	-1.962492228
59.75	-1.749809869	0.001726218	hsa-miR-193a-3p	1137.5	-1.971025347
127.5	1.525733982	0.001904849	hsa-let-7e-3p	17.5	-2.017029441
56.75	-1.633596495	0.002030203	hsa-let-7d-3p	26.75	-2.088460447
29.75	-2.036334229	0.002675513	hsa-miR-331-3p	319.25	-2.089539324
18.5	-2.402434335	0.002993996	hsa-miR-22-5p	31.75	-2.092955454
10.25	2.679966721	0.00326778	hsa-miR-200b-3p	13	-2.16420509
20.75	-2.759403283	0.003423449	hsa-miR-33b-3p	11.75	-2.171651994
17.5	-2.372672046	0.005088075	hsa-miR-31-3p	94.5	-2.197238955
42.25	-1.676418361	0.005208055	hsa-miR-513a-5p	19	-2.265469261
195.75	1.389378667	0.005415414	hsa-miR-450b-5p	67.25	-2.273445239
5	18.63472459	0.005670558	hsa-miR-452-5p	42.25	-2.288597715
10	-3.616244916	0.005804252	hsa-miR-324-3p	9.75	-2.303248031
7.5	-9.350584387	0.006177602	hsa-miR-326	27.5	-2.314767863

6	3.825983329	0.006417431	hsa-miR-578	10.5	-2.31852434
288	1.279280034	0.006417431	hsa-miR-2355-5p	13.5	-2.362922714
54.25	-1.561852653	0.006601283	hsa-miR-125a-3p	43.75	-2.371308598
58.5	1.451582923	0.007310918	hsa-miR-487a-5p	12.25	-2.393468921
26.25	-1.878005963	0.007774057	hsa-miR-27a-5p	59.75	-2.39610817
25622.5	1.216963154	0.008017374	hsa-miR-23a-5p	29.75	-2.432377448
22	-1.999970386	0.008017374	hsa-miR-582-5p	142.75	-2.472884561
8.5	-4.033674136	0.008726532	hsa-miR-4446-3p	55	-2.533770474
241.25	-1.313972414	0.008951781	hsa-miR-193b-5p	25.75	-2.862037676
4.25	6.689729818	0.00948781	hsa-miR-4425	35.75	-2.951290464
13.25	2.108418065	0.009513116	hsa-let-7i-3p	17.5	-3.053714967
4.75	4.717306197	0.009905534	hsa-miR-5089-5p	7.25	-3.33457132
12	-2.725383898	0.010109989	hsa-miR-6741-5p	59	-3.525322758
5	4.192200384	0.010737139	hsa-miR-192-3p	10.5	-4.234113822
20541.75	1.208786472	0.010924701	hsa-miR-4718	6.75	-4.243825593
96.5	1.346902424	0.01131455	hsa-miR-342-5p	7	-4.400613668
9.25	2.419874001	0.011377144	hsa-miR-9900	149	-4.816584447
25.75	-1.88876832	0.011573122	hsa-miR-134-3p	5	-4.99197571
9.25	-3.864934748	0.011573122	hsa-miR-2110	6.5	-5.008201314
13.25	-2.459087576	0.011779686	hsa-miR-141-5p	5.75	-5.718248061
12.75	-2.517820106	0.011822604	hsa-miR-6070	7.5	-7.423616315
4	14.98888442	0.012076181	hsa-miR-6784-5p	6.5	-9.059387782
17.5	-2.094163757	0.012687572	hsa-miR-6737-5p	115.5	-9.494417894
56.5	1.448802954	0.013651338	hsa-miR-6774-3p	10.75	-10.5871185
10.5	-2.800582168	0.014503957	hsa-miR-3940-5p	22	-16.81738267
7	-4.821712438	0.01453124			
175	1.353028042	0.016322977			
1881	1.204763125	0.016815549			
4219.5	1.19927746	0.017181979			
27	1.585287751	0.017676901			
3.5	7.800047988	0.017823692			
80.75	-1.389868119	0.018921852			
149	-2.479379695	0.018921852			
52.5	-1.524702603	0.019187432			
84.5	-1.418611493	0.019272586			
128	1.380167899	0.019337886			
566.5	1.268632115	0.021939358			
13.75	-2.154982023	0.021939358			
5.25	3.358382989	0.022186742			
3.25	7.260437806	0.023680936			
3.25	7.257327375	0.02404583			
35.75	-2.043021753	0.024051002			
7.25	-3.446670857	0.024162883			
17	-1.95775673	0.024247597			
111.75	-1.397277477	0.024262906			

1451.5	-1.195647349	0.02593104
176.25	-1.373216113	0.02593104
21	1.693107526	0.028997656
12.25	-2.31641731	0.030767705
5.5	-4.899300342	0.031106567
6.75	-3.212284992	0.039203905
99.75	-1.353381289	0.042820926
4.5	-9.535056514	0.045907802
32	-1.533134065	0.047570521
11.5	-2.134403945	0.047570521
2.75	6.18102734	0.047854391
4.25	3.09775598	0.047854391
7.25	-3.019291789	0.047854391
23.5	-1.642125027	0.048366114
12.25	-2.027163044	0.048366114
7.5	-2.721101328	0.048366114

PI Ad14p1 v C 24HPI Ad14

FDR p-value	Identifier	Max group mean	Fold change	FDR p-value	Identifier
0.041380785	hsa-miR-6841-5p	17.5	146.6999358	0.002256142	hsa-miR-6841-5p
0.04904175	hsa-miR-10524-5p	14.75	124.4069907	0.003214475	hsa-miR-10524-5p
0.002087178	hsa-miR-4661-3p	12	35.31082274	0.000173434	hsa-miR-518a-5p
0.005232353	hsa-miR-518a-5p	10.25	30.16048955	0.000312876	hsa-miR-4661-3p
0.006542093	hsa-miR-4668-3p	6.5	19.26972423	0.002749831	hsa-miR-3128
0.023551115	hsa-miR-155-5p	10.75	19.13164636	6.07305E-05	hsa-miR-1197
0.041121933	hsa-miR-1197	25.5	17.46608391	6.18522E-11	hsa-miR-4668-3p
0.000495066	hsa-miR-5571-5p	4.75	14.18721837	0.009591098	hsa-miR-4490
8.15796E-05	hsa-miR-375-5p	4.75	14.18648751	0.009364267	hsa-miR-4279
0.002178744	hsa-miR-668-5p	58	12.67561642	1.45871E-21	hsa-miR-155-5p
0.001295484	hsa-miR-4279	12.5	12.40892696	4.3678E-06	hsa-miR-4724-5p
0.011244053	hsa-miR-4490	4	12.00907028	0.017495062	hsa-miR-668-5p
0.000168304	hsa-miR-4714-5p	85.75	11.40432052	9.93041E-35	hsa-miR-5571-5p
0.044404514	hsa-miR-4724-5p	5	9.023828617	0.006140873	hsa-miR-888-5p
0.012564097	hsa-miR-6799-5p	4.5	8.145576632	0.010081031	hsa-miR-520f-3p
1.71614E-07	hsa-miR-4431	18	7.665048309	1.77381E-08	hsa-miR-4714-5p
1.73267E-05	hsa-miR-888-5p	14.25	7.532660828	3.45652E-06	hsa-miR-6799-5p
0.006505105	hsa-miR-3128	15.5	7.269181339	9.31326E-07	hsa-miR-4462
0.041121933	hsa-miR-520f-3p	9.25	6.396144743	0.000135823	hsa-miR-3125
0.017805593	hsa-miR-6870-3p	3.25	5.959980403	0.044996778	hsa-miR-4759
2.01288E-12	hsa-miR-369-3p	11.25	5.941089308	2.40958E-05	hsa-miR-4286
0.003987982	hsa-miR-1253	15	5.895000333	7.28844E-06	hsa-miR-1253
0.021478913	hsa-miR-4496	9.25	5.545261848	0.00018734	hsa-miR-548c-3p
3.81278E-11	hsa-miR-885-3p	3.75	4.89416878	0.033499608	hsa-miR-4299
1.0487E-13	hsa-miR-493-3p	7	4.863988171	0.002999772	hsa-miR-215-3p
2.83841E-10	hsa-miR-4722-5p	4.25	4.306561423	0.027730732	hsa-miR-1245b-3p
5.03204E-09	hsa-miR-6792-5p	4.25	4.305742337	0.027730732	hsa-miR-369-3p
0.018455906	hsa-miR-431-5p	8	4.244035403	0.001580028	hsa-miR-33a-3p
0.01603807	hsa-miR-4286	81.25	4.149316235	3.61666E-15	hsa-miR-1261
1.17977E-05	hsa-miR-1321	10.25	4.007745297	0.000512282	hsa-miR-518a-3p
2.26937E-09	hsa-miR-889-3p	36.75	3.953129941	3.38194E-11	hsa-miR-5683
6.07653E-07	hsa-miR-1245b-3p	14.75	3.77844637	2.18854E-05	hsa-miR-646
0.000103444	hsa-miR-129-5p	4.5	3.728211717	0.046302955	hsa-miR-3156-3p
2.74147E-06	hsa-miR-649	5.25	3.669213489	0.019238167	hsa-miR-889-3p
2.318E-06	hsa-miR-5187-3p	16.75	3.49006048	1.20257E-05	hsa-miR-4431
0.012329875	hsa-miR-5683	13.75	3.334620395	0.000164261	hsa-miR-431-5p
0.049905117	hsa-miR-556-5p	10.5	3.257557529	0.000954223	hsa-miR-3649
0.00776634	hsa-miR-548n	8.5	3.066738886	0.005319228	hsa-miR-520g-5p
0.00025867	hsa-miR-410-3p	98.75	3.047260195	1.32413E-20	hsa-miR-1301-5p
2.2772E-05	hsa-miR-136-5p	5	3.0340709	0.048927873	hsa-miR-12120
0.007468368	hsa-miR-9-5p	104	3.001222388	6.24795E-21	hsa-miR-9-5p

2.14773E-05	hsa-miR-300	202	2.999296831	1.20287E-31	hsa-miR-492
0.035604584	hsa-miR-3156-3p	26.25	2.897202745	3.05926E-07	hsa-miR-410-3p
0.010941786	hsa-miR-127-3p	654.25	2.896955364	3.77608E-39	hsa-miR-8087
0.002681342	hsa-miR-152-5p	7.25	2.850131987	0.017495062	hsa-miR-4474-3p
0.01603807	hsa-miR-485-3p	8.25	2.758440267	0.010081031	hsa-miR-1266-5p
0.04904175	hsa-miR-143-3p	295	2.594451698	2.10397E-18	hsa-miR-493-5p
0.017127771	hsa-miR-1266-5p	7	2.534670195	0.032510208	hsa-miR-4511
0.014151984	hsa-miR-3620-3p	9.25	2.526504153	0.014057157	hsa-miR-106b-3p
0.034868963	hsa-miR-146b-3p	11.25	2.468587536	0.007004278	hsa-miR-300
0.038093335	hsa-miR-106b-3p	128.75	2.4319943	1.30004E-15	hsa-miR-8066
0.032110946	hsa-miR-487b-3p	7.75	2.415119424	0.027850742	hsa-miR-1276
0.032110946	hsa-miR-411-5p	97.75	2.387299286	2.61085E-14	hsa-miR-127-3p
0.02207209	hsa-miR-375-3p	150.5	2.323101417	9.98662E-18	hsa-miR-1321
0.012018506	hsa-miR-3654	8.75	2.256215316	0.032024731	hsa-miR-375-3p
0.006429775	hsa-miR-6131	17	2.251105128	0.005530072	hsa-miR-7-5p
0.01143671	hsa-miR-5009-5p	88	2.211063472	1.62861E-11	hsa-miR-1285-3p
0.004134013	hsa-miR-493-5p	10	2.196505398	0.020522929	hsa-miR-3620-3p
0.000513279	hsa-miR-132-3p	84.5	2.195212032	1.41582E-08	hsa-miR-5187-3p
0.000202706	hsa-miR-409-3p	56.25	2.117282355	7.14421E-07	hsa-miR-7706
0.014523315	hsa-miR-193a-5p	80	2.069680708	4.82126E-07	hsa-miR-1304-3p
1.58946E-05	hsa-miR-136-3p	31.25	2.037495789	0.000164261	hsa-miR-5009-5p
0.001485711	hsa-miR-7706	82.25	1.98769674	8.73246E-09	hsa-miR-411-5p
0.000324401	hsa-miR-1285-3p	45.5	1.981716469	3.80249E-05	hsa-miR-2682-5p
7.82898E-06	hsa-miR-378a-3p	1522.25	1.980477048	8.7415E-25	hsa-miR-589-3p
1.65824E-06	hsa-miR-7-5p	37	1.979920368	0.000114968	hsa-miR-4677-3p
2.0997E-06	hsa-miR-2682-5p	10.75	1.974940757	0.040240253	hsa-miR-548a-5p
0.000244086	hsa-miR-6890-3p	17.5	1.936167521	0.008379084	hsa-miR-143-3p
0.018455906	hsa-miR-7158-3p	36.75	1.855449828	0.000250412	hsa-miR-7113-3p
2.1584E-08	hsa-miR-3184-5p	932.75	1.840476363	5.78808E-13	hsa-miR-378a-3p
6.07653E-07	hsa-miR-548a-5p	14	1.822228924	0.042296983	hsa-miR-409-3p
0.021478913	hsa-miR-148a-3p	987.25	1.821215893	2.50666E-18	hsa-miR-148a-3p
1.39599E-07	hsa-miR-101-3p	506.75	1.810346156	9.39113E-16	hsa-miR-3613-5p
1.91602E-07	hsa-miR-8087	34.25	1.803678914	0.013659231	hsa-miR-25-3p
0.043056107	hsa-miR-12135	650	1.790881685	2.44199E-11	hsa-miR-4499
0.014831213	hsa-miR-532-5p	178.75	1.770488641	4.51471E-10	hsa-miR-7158-3p
3.00264E-08	hsa-miR-671-3p	45.25	1.733891468	0.00057178	hsa-miR-532-5p
0.001485711	hsa-miR-181a-5p	23304.25	1.727513375	1.19346E-10	hsa-miR-193a-5p
0.032110946	hsa-miR-25-3p	22837.75	1.724822252	6.85668E-18	hsa-miR-3176
0.043056107	hsa-miR-8066	50.5	1.711005143	0.002104529	hsa-miR-4492
3.26167E-06	hsa-miR-3615	49.25	1.680177694	0.002158321	hsa-miR-1269a
0.006559594	hsa-miR-192-5p	49640.75	1.614386073	6.95434E-07	hsa-miR-181a-5p
1.51158E-05	hsa-miR-1269a	33	1.543605337	0.016829866	hsa-miR-3184-5p
0.000104005	hsa-miR-1304-3p	31	1.531627195	0.036517335	hsa-miR-192-5p
0.049905117	hsa-miR-340-5p	321.5	1.518095902	2.67957E-07	hsa-miR-132-3p
0.001485711	hsa-miR-769-5p	471	1.442357508	1.95135E-06	hsa-miR-101-3p

0.042117758	hsa-miR-34a-5p	428.5	1.426575862	5.91449E-06	hsa-miR-186-5p
0.017805593	hsa-miR-543	67.5	1.371700476	0.019238167	hsa-miR-340-5p
5.6427E-05	hsa-miR-1827	63.25	1.340754088	0.044996778	hsa-miR-671-3p
0.031425168	hsa-miR-182-5p	14762.5	1.33320864	2.18854E-05	hsa-miR-182-5p
9.58343E-05	hsa-miR-1307-3p	111.75	1.318928431	0.014740977	hsa-miR-769-5p
0.000206753	hsa-miR-27a-3p	84171.25	1.318431754	4.61125E-05	hsa-miR-12135
3.75739E-10	hsa-miR-186-5p	937	1.317888533	0.00018734	hsa-miR-151a-3p
0.001485711	hsa-miR-561-5p	92.5	1.317142395	0.02375061	hsa-miR-126-5p
2.78572E-05	hsa-miR-423-3p	861	1.311988298	0.015366841	hsa-miR-194-5p
2.30311E-05	hsa-miR-151a-3p	4498	1.307721479	0.000114927	hsa-miR-28-5p
0.000406054	hsa-miR-126-5p	215.25	1.307377427	0.004177148	hsa-miR-7974
0.029126038	hsa-miR-28-3p	3670	1.263935424	0.001033302	hsa-miR-151a-5p
0.001680462	hsa-miR-22-3p	28627.5	-1.27779398	0.000396665	hsa-miR-1297
0.002792285	hsa-miR-1297	6967.25	-1.288975787	0.000259209	hsa-miR-181a-2-3p
0.019130172	hsa-miR-28-5p	241.25	-1.289974109	0.007084033	hsa-miR-345-5p
0.016301813	hsa-miR-210-3p	270	-1.295996532	0.004648724	hsa-miR-22-3p
0.00031005	hsa-miR-5682	99.75	-1.298478382	0.044076664	hsa-miR-4446-3p
0.045411897	hsa-miR-345-5p	235.25	-1.325765094	0.009907573	hsa-miR-4659a-5p
0.019130172	hsa-miR-151a-5p	2481.25	-1.361898482	0.001306185	hsa-miR-3155a
0.021805961	hsa-miR-181a-2-3p	418.5	-1.384808561	6.00575E-05	hsa-miR-4502
0.005671851	hsa-miR-196a-5p	128	-1.392669187	0.019957831	hsa-miR-30c-1-3p
0.010941786	hsa-miR-335-3p	176.25	-1.424016983	0.000304488	hsa-miR-134-5p
1.44039E-18	hsa-miR-214-5p	88.75	-1.445275543	0.042296983	hsa-miR-210-3p
0.000661193	hsa-miR-342-3p	55.5	-1.464159302	0.016351721	hsa-miR-3606-3p
1.04069E-06	hsa-miR-224-5p	111	-1.469042548	0.003518159	hsa-miR-374a-5p
	hsa-miR-197-3p	84.5	-1.472807419	0.002158321	hsa-miR-29a-3p
	hsa-miR-29a-3p	1045.75	-1.529585236	1.63457E-09	hsa-miR-224-5p
	hsa-miR-3155a	369	-1.572403617	1.8587E-08	hsa-miR-24-3p
	hsa-miR-181a-3p	209.25	-1.600251101	2.76551E-07	hsa-miR-181a-3p
	hsa-miR-374a-5p	111.75	-1.612960293	1.84329E-05	hsa-miR-15b-3p
	hsa-miR-3165	25.75	-1.617165385	0.028931576	hsa-miR-125b-1-3p
	hsa-miR-125a-5p	5732	-1.618661399	5.72803E-07	hsa-miR-574-3p
	hsa-miR-24-3p	729.75	-1.621130966	2.40094E-11	hsa-let-7i-3p
	hsa-miR-193b-5p	25.75	-1.635440065	0.048519654	hsa-miR-125a-5p
	hsa-miR-320e	38.75	-1.652276373	0.006464854	hsa-miR-23a-3p
	hsa-miR-450b-5p	67.25	-1.66129945	0.001279053	hsa-miR-365a-3p
	hsa-miR-3606-3p	63.75	-1.673975099	0.000682994	hsa-miR-450a-5p
	hsa-miR-365a-3p	238.75	-1.682838958	3.517E-09	hsa-let-7d-3p
	hsa-miR-574-3p	61.25	-1.683641297	0.001522066	hsa-miR-31-3p
	hsa-miR-15b-3p	51.25	-1.733374782	0.004573892	hsa-miR-214-5p
	hsa-let-7a-3p	84.75	-1.747452056	6.57125E-06	hsa-miR-3165
	hsa-miR-542-3p	23.5	-1.812777531	0.032929171	hsa-miR-331-3p
	hsa-miR-23a-3p	1803.5	-1.867717201	5.56887E-21	hsa-miR-193a-3p
	hsa-miR-424-3p	32	-1.876472569	0.005192507	hsa-let-7a-3p
	hsa-let-7i-3p	17.5	-1.889235025	0.018521346	hsa-miR-4282

hsa-miR-31-3p	94.5	-1.91747758	4.5737E-08	hsa-miR-4650-3p
hsa-miR-4282	54.25	-1.924402721	2.32159E-05	hsa-miR-601
hsa-miR-4650-3p	22.25	-1.935446277	0.007172993	hsa-miR-222-5p
hsa-miR-193a-3p	1137.5	-2.011570739	1.86171E-24	hsa-miR-424-3p
hsa-miR-452-5p	42.25	-2.013413179	0.000122497	hsa-miR-582-5p
hsa-let-7d-3p	26.75	-2.054013503	0.0042833	hsa-miR-452-5p
hsa-miR-125b-1-3p	20.75	-2.054604677	0.002696089	hsa-miR-200b-3p
hsa-miR-331-3p	319.25	-2.152959373	2.70724E-15	hsa-miR-9900
hsa-miR-3691-3p	12	-2.232261316	0.016351721	hsa-miR-542-3p
hsa-miR-601	29.25	-2.269060734	8.62899E-05	hsa-miR-513a-5p
hsa-miR-9900	149	-2.301348367	7.65011E-16	hsa-miR-26a-1-3p
hsa-miR-450a-5p	26.25	-2.306350225	0.001445636	hsa-let-7e-3p
hsa-miR-582-5p	142.75	-2.30780123	8.42442E-13	hsa-miR-6741-5p
hsa-miR-4435	10	-2.343861186	0.023461004	hsa-miR-125a-3p
hsa-miR-6850-3p	8.75	-2.375124892	0.04715106	hsa-miR-4425
hsa-miR-27b-5p	343.75	-2.41153794	1.75154E-26	hsa-miR-10a-3p
hsa-miR-4425	35.75	-2.503415087	1.13757E-05	hsa-miR-365a-5p
hsa-miR-6728-5p	12.75	-2.639897628	0.004132465	hsa-miR-27b-5p
hsa-miR-125a-3p	43.75	-2.648108543	1.07066E-07	hsa-miR-193b-5p
hsa-miR-513a-5p	19	-2.68040177	0.000614751	hsa-miR-4292
hsa-miR-6734-5p	11.5	-2.685348892	0.009670706	hsa-miR-5089-5p
hsa-miR-6741-5p	59	-2.737279465	7.51588E-08	hsa-miR-6847-3p
hsa-miR-6784-5p	6.5	-2.79279604	0.042296983	hsa-miR-3978
hsa-miR-6125	9.25	-2.806902202	0.042296983	hsa-miR-141-5p
hsa-let-7e-3p	17.5	-2.942091611	0.000194996	hsa-miR-134-3p
hsa-miR-6808-5p	6.5	-3.163659155	0.026876936	hsa-miR-3691-3p
hsa-miR-192-3p	10.5	-3.314311545	0.004177148	hsa-miR-6850-3p
hsa-miR-5089-5p	7.25	-3.520816927	0.012080857	hsa-miR-6125
hsa-miR-365a-5p	12.25	-3.550434332	0.000593525	hsa-miR-647
hsa-miR-29b-1-5p	9	-3.845865458	0.002999772	hsa-miR-27a-5p
hsa-miR-4666b	4.75	-3.868622858	0.043115816	hsa-miR-6774-3p
hsa-miR-875-3p	9.25	-3.964296648	0.005319228	hsa-miR-6070
hsa-miR-222-5p	19.75	-4.254883042	1.68835E-05	hsa-miR-4718
hsa-miR-4718	6.75	-4.468495741	0.00853684	hsa-miR-6850-5p
hsa-miR-33b-3p	11.75	-4.47949024	0.000249122	hsa-miR-4303
hsa-miR-342-5p	7	-4.631447527	0.007004278	hsa-miR-33b-3p
hsa-miR-23a-5p	29.75	-4.987753929	8.62253E-09	hsa-miR-23a-5p
hsa-miR-4303	5.25	-5.471866968	0.016752189	hsa-miR-6728-5p
hsa-miR-27a-5p	59.75	-6.209496648	2.47828E-16	hsa-miR-342-5p
hsa-miR-5587-3p	4.5	-6.567815593	0.02490893	hsa-miR-6737-5p
hsa-miR-6774-3p	10.75	-7.066744292	0.000173398	hsa-miR-3940-5p
hsa-miR-6737-5p	115.5	-9.230006921	1.34179E-32	
hsa-miR-6070	7.5	-10.8179573	0.002154838	
hsa-miR-6752-5p	4.5	-10.84212361	0.02606927	
hsa-miR-3940-5p	22	-17.47132824	1.02368E-07	

4p1 v C 36HPI   Ad14p1 v C 48HP

Max group mean	Fold change	FDR p-value	Identifier	Max group mean	Fold change
13	111.8774827	0.002585727	hsa-miR-8077	18	111.6841957
10.25	88.43632392	0.004598096	hsa-miR-518a-5p	46.5	107.7627164
15.75	49.34835163	2.00696E-05	hsa-miR-499b-5p	15.5	96.24142476
6.5	20.55117935	0.001654959	hsa-miR-4724-5p	37.5	53.47115708
37.25	19.03923794	1.95098E-16	hsa-miR-5008-5p	7	42.15384151
21.75	16.22846911	2.55803E-10	hsa-miR-4440	6.5	40.74714374
5	15.53063934	0.007538347	hsa-miR-646	65.5	36.65519694
4.5	14.35031197	0.007474469	hsa-miR-6802-3p	5.75	36.12139902
12.75	13.73329374	1.33323E-06	hsa-miR-6841-5p	5.75	35.97531384
6.75	13.04103779	0.000754786	hsa-miR-4286	845.25	35.53372348
6.5	12.56587769	0.000953076	hsa-miR-10524-5p	5	31.49837834
45.75	10.86842901	3.41648E-21	hsa-miR-7850-5p	13.25	30.76733686
2.75	8.922585641	0.046167916	hsa-miR-520f-3p	53.25	29.76077057
14.5	8.889362469	1.11504E-06	hsa-miR-4462	20.75	29.51806441
11.75	8.821435048	3.64052E-06	hsa-miR-1253	81.25	25.79937814
55.75	8.116550084	1.19419E-24	hsa-miR-551a	31.5	25.29185058
4	7.824024343	0.011328433	hsa-miR-596	28.75	23.08476657
3.75	7.349689134	0.017783988	hsa-miR-3168	86	22.96925854
5	6.999890517	0.00450588	hsa-miR-3125	21.25	21.84425674
3.5	6.877112045	0.021292681	hsa-miR-1261	45.25	21.81313517
119.5	6.725080448	1.03219E-35	hsa-miR-124-3p	9.25	21.55649267
15	6.451742865	7.24191E-07	hsa-miR-6888-5p	8.5	19.82472699
9.75	6.362316554	4.3912E-05	hsa-miR-4668-3p	8.5	19.66115233
10.75	6.19045729	1.74108E-05	hsa-miR-155-5p	13.5	19.33518306
3	5.928406949	0.040959118	hsa-miR-3128	50	19.19268147
19.25	5.159997085	9.28819E-07	hsa-miR-12120	74.75	18.96236181
8.5	5.120142801	0.000987569	hsa-miR-412-3p	7.5	17.52604534
15.5	5.071048763	1.63843E-05	hsa-miR-6877-5p	12	17.14935488
7.5	4.913894705	0.000892917	hsa-miR-4759	11.5	16.44310312
4.25	4.668086234	0.017300492	hsa-miR-4661-3p	7	16.37260148
17.5	4.625861787	1.13909E-07	hsa-miR-4696	20.25	16.28819986
6	4.608094317	0.008263164	hsa-miR-4260	24	15.83488102
36.25	4.372678936	2.39138E-14	hsa-miR-1296-3p	11	15.72905154
36	4.240496437	5.27349E-14	hsa-miR-12113	19.5	15.68886951
9	4.212135935	0.000474499	hsa-miR-6762-5p	10.5	15.025418
7.25	4.197856617	0.002888642	hsa-miR-4754	10.5	15.02241803
9.25	3.953663663	0.00052624	hsa-miR-4481	6.25	14.64497721
6.75	3.912413164	0.003324203	hsa-miR-520g-5p	32.75	14.01667933
6	3.485330154	0.009233216	hsa-miR-200a-5p	17	13.68833619
10.25	3.475270004	0.000696289	hsa-miR-296-3p	9.25	13.25346041
106.75	3.348619593	1.30459E-18	hsa-miR-4299	27.5	11.80978755

7.5	3.250816517	0.032287576	hsa-miR-3154	11.25	11.61222061
92.5	3.121417855	3.00681E-17	hsa-miR-888-3p	4.75	11.18952287
53.5	3.105158936	4.56337E-11	hsa-miR-6763-3p	4.75	11.16904398
5.25	3.059063776	0.027117276	hsa-miR-492	35	11.14047159
7.75	3.056923156	0.006470966	hsa-miR-4323	7.75	11.12905382
12.25	2.933614531	0.000696289	hsa-miR-302f	10.25	10.59014501
11	2.923704274	0.001132918	hsa-miR-888-5p	23.25	10.00230251
140.5	2.89971719	1.30459E-18	hsa-miR-3136-3p	6.75	9.930891972
170	2.733036183	1.53365E-17	hsa-miR-6079	9.5	9.862232306
74.25	2.71596924	1.52716E-10	hsa-miR-4431	28.25	9.843559433
8.5	2.700603482	0.01231764	hsa-miR-33a-3p	40.75	9.632645146
560	2.676352299	1.43227E-27	hsa-miR-3928-3p	22	9.441600955
6.25	2.673836554	0.047371504	hsa-miR-6866-5p	4	9.31408261
155.25	2.623030176	3.41648E-21	hsa-miR-665	33.5	9.102687613
44.25	2.589374335	1.60124E-09	hsa-miR-3911	8.75	9.058037092
54	2.573607234	6.63455E-11	hsa-miR-5100	21	8.990252931
8.5	2.539725899	0.011552442	hsa-miR-196a-1-3p	23.25	8.952265622
11	2.514237904	0.003919977	hsa-miR-759	6.25	8.908041039
88.25	2.329189585	7.34693E-11	hsa-miR-299-5p	6	8.650395565
42.75	2.306995148	1.04068E-07	hsa-miR-3193	8.25	8.545706127
83	2.283668835	1.74114E-11	hsa-miR-3610	8.25	8.530025701
86.25	2.267253555	4.10505E-09	hsa-miR-4508	33	8.34209565
10.75	2.1568401	0.02403568	hsa-miR-218-1-3p	24	8.319683728
21.25	2.100358585	0.00121319	hsa-miR-3649	25.75	8.200978161
13.5	2.091959841	0.025762042	hsa-miR-518d-5p	32	8.088832102
14.5	2.053409172	0.017466813	hsa-miR-4762-3p	25.25	8.048971522
217.5	2.037214073	9.07395E-08	hsa-miR-1301-5p	18.25	7.828110149
9.25	2.026407273	0.038662288	hsa-miR-6085	7.5	7.779046166
1423	1.989703849	5.56636E-18	hsa-miR-134-3p	43	7.686738826
46.5	1.911034126	2.30556E-05	hsa-miR-219b-3p	7	7.370702961
952.75	1.875910315	1.90688E-11	hsa-miR-4781-3p	25	7.331129563
13	1.852018453	0.030840823	hsa-miR-6753-5p	7	7.267491471
22915.25	1.848823336	6.50375E-15	hsa-miR-3678-3p	5	7.234041669
22.25	1.832476379	0.004192479	hsa-miR-7-5p	169.75	7.228518455
32	1.769101123	0.001189616	hsa-miR-6819-3p	9	7.122236935
162	1.744275281	1.09013E-06	hsa-miR-4279	8.75	7.087394898
60.75	1.704478868	0.000124549	hsa-miR-483-5p	29.5	7.084072218
19	1.677410648	0.020550286	hsa-miR-6749-3p	19.75	6.89241036
18.75	1.655530831	0.032011401	hsa-miR-5692b	8.5	6.887671275
32.25	1.650411921	0.004430824	hsa-miR-2114-3p	4.75	6.879378137
20364.25	1.63080805	2.81508E-10	hsa-miR-4489	17.75	6.841994157
732.25	1.563205122	0.000268192	hsa-miR-1305	6.5	6.756823956
44262.75	1.515466999	3.2449E-05	hsa-miR-6081	6.5	6.756731953
52.75	1.494083398	0.00580898	hsa-miR-3150b-3p	26.5	6.708683688
389.75	1.469318677	0.001794921	hsa-miR-330-3p	27.25	6.560148379

967.5	1.461252282	1.33088E-05	hsa-miR-203b-3p	15.25	6.558139075
282.5	1.435465907	0.001197074	hsa-miR-6873-5p	4.5	6.536839707
34	1.427626504	0.047371504	hsa-miR-3685	4.5	6.525929307
14831	1.425219674	9.55755E-05	hsa-miR-626	4.5	6.525538319
434	1.423329372	0.00161221	hsa-miR-8080	38	6.487622685
463	1.373109186	0.001654959	hsa-miR-5089-5p	52	6.474332156
4347.75	1.338434489	0.003266249	hsa-miR-4654	38.75	6.337934271
205.25	1.327546878	0.034129631	hsa-miR-556-3p	6	6.245423284
1295.5	-1.232797558	0.049433699	hsa-miR-7851-3p	4.25	6.171725793
241.25	-1.322400202	0.01589475	hsa-miR-6510-5p	4.25	6.171109856
100.75	-1.404118682	0.00523035	hsa-miR-1288-3p	4.25	6.165947535
2481.25	-1.411287757	0.001654959	hsa-miR-6165	12.5	6.102088863
6967.25	-1.412679468	2.16846E-07	hsa-miR-580-3p	7.5	6.086242801
418.5	-1.414548784	0.000989502	hsa-miR-12136	40.75	6.080542085
235.25	-1.423452571	0.002218114	hsa-miR-1469	15.5	6.057119697
28627.5	-1.4773713	1.11539E-06	hsa-miR-1236-5p	5.75	6.048206265
55	-1.480778492	0.025232118	hsa-miR-5692a	7.25	5.828430614
44	-1.48696331	0.022167143	hsa-miR-4686	4	5.818825913
369	-1.509661161	0.008456732	hsa-miR-12114	8.75	5.818695496
29.75	-1.546263768	0.039254867	hsa-miR-6799-5p	4	5.817906861
47.25	-1.630756096	0.006467387	hsa-miR-1825	4	5.817260595
33.75	-1.646680112	0.006826131	hsa-miR-4433b-3p	5.5	5.733672176
270	-1.648278412	3.47872E-05	hsa-miR-4773	7	5.686904028
63.75	-1.656579006	0.000481085	hsa-miR-3620-3p	25	5.579889932
111.75	-1.668375227	0.000956423	hsa-miR-3156-3p	61.5	5.577729188
1045.75	-1.676340662	6.48067E-09	hsa-miR-8083	6.75	5.566932272
111	-1.681754271	0.000127021	hsa-miR-6757-5p	12.75	5.500150368
729.75	-1.693741237	1.87066E-07	hsa-miR-4443	5.25	5.478520849
209.25	-1.713454017	8.41641E-05	hsa-miR-6768-5p	3.75	5.46391814
51.25	-1.729441929	0.000587988	hsa-miR-4424	9.5	5.356015159
20.75	-1.736445073	0.036329211	hsa-miR-6833-5p	6.5	5.287142231
61.25	-1.777388095	0.001862657	hsa-miR-5699-5p	9.25	5.216160889
17.5	-1.780666903	0.039155471	hsa-miR-7113-3p	31.75	5.185189305
5732	-1.876664336	7.11474E-10	hsa-miR-6783-5p	20.25	5.1434762
1803.5	-1.921424806	3.09029E-13	hsa-miR-1197	9	5.080017862
238.75	-1.935295017	1.22068E-06	hsa-miR-642a-3p	11.75	5.077332027
26.25	-1.943912808	0.00196597	hsa-miR-4691-3p	4.75	4.966981744
26.75	-1.958671096	0.004598096	hsa-miR-12128	4.75	4.966556285
94.5	-1.961253108	7.24191E-07	hsa-miR-6776-3p	6	4.88662012
88.75	-1.961387124	5.83768E-06	hsa-miR-1276	20.5	4.864994153
25.75	-1.987923471	0.005420812	hsa-miR-4433a-3p	9.75	4.841176597
319.25	-1.990733614	4.02046E-09	hsa-miR-5584-5p	9.75	4.771392048
1137.5	-2.060214476	6.70961E-13	hsa-miR-4776-5p	10.5	4.538339046
84.75	-2.060539568	3.09382E-08	hsa-miR-1268a	8	4.492164562
54.25	-2.063869836	8.32824E-07	hsa-miR-4699-5p	4.25	4.455456163

22.25	-2.067320622	0.002218114	hsa-miR-432-3p	7.75	4.378961991
29.25	-2.119862784	0.000268192	hsa-miR-1245b-3p	20.75	4.358583489
19.75	-2.141971779	0.011297456	hsa-miR-4254	6.5	4.341466521
32	-2.175742911	7.78463E-05	hsa-miR-6736-3p	5.25	4.331319732
142.75	-2.313162638	6.86679E-10	hsa-miR-5691	19.25	4.293469263
42.25	-2.459747621	7.95975E-07	hsa-miR-6784-5p	30.5	4.231984609
13	-2.464931891	0.007659698	hsa-miR-1199-5p	6.25	4.177267222
149	-2.525590898	1.3192E-10	hsa-miR-3917	8.5	4.167112685
23.5	-2.54142776	0.000558906	hsa-miR-6793-5p	5	4.086362703
19	-2.670717631	0.000979198	hsa-miR-4475	7	3.959077665
9.25	-2.677589015	0.025762042	hsa-miR-10392-3p	8	3.936759061
17.5	-2.692448042	0.000989502	hsa-miR-668-5p	21.5	3.858639412
59	-2.70881196	4.13034E-10	hsa-miR-3169	5.75	3.856253618
43.75	-2.742556425	1.01451E-08	hsa-miR-6823-5p	5.5	3.684477248
35.75	-2.770577934	3.16572E-06	hsa-miR-6816-3p	8	3.468054236
13	-2.972937493	0.002181299	hsa-miR-4648	8.25	3.460008201
12.25	-3.011544874	0.002797744	hsa-miR-5683	16.75	3.333143834
343.75	-3.047230814	3.59797E-22	hsa-miR-1266-5p	11.25	3.332232724
25.75	-3.08734618	5.49591E-06	hsa-miR-495-3p	9.25	3.247402086
14	-3.211639857	0.000989502	hsa-miR-4511	16	3.210796615
7.25	-3.231848552	0.020729573	hsa-miR-8087	70.5	3.09193126
9.5	-3.329544023	0.006412804	hsa-miR-4318	13	3.090419521
20.75	-3.347415714	0.000525248	hsa-miR-1238-5p	8.75	3.073287751
5.75	-3.513796156	0.036329211	hsa-miR-324-5p	43	3.064253038
5	-3.747668127	0.047742975	hsa-miR-3613-5p	28.5	3.041487669
12	-3.79361216	0.000953076	hsa-miR-889-5p	16.75	3.010460361
8.75	-4.052866557	0.010071719	hsa-miR-4522	7.75	2.986774444
9.25	-4.561950425	0.006467387	hsa-miR-4436a	10	2.950908053
5	-4.751581821	0.047429664	hsa-miR-548a-5p	27.25	2.888637295
59.75	-4.995871987	4.1175E-15	hsa-miR-1908-3p	15.25	2.881862448
10.75	-5.530125422	0.000989502	hsa-miR-548u	38.75	2.880817446
7.5	-5.568879922	0.004898444	hsa-miR-4714-5p	25.75	2.851220032
6.75	-6.455274597	0.005988459	hsa-miR-517-5p	12.5	2.793677255
5.25	-7.068128445	0.017466813	hsa-miR-3960	9	2.658646097
5.25	-7.068969836	0.017300492	hsa-miR-548n	9	2.658317412
11.75	-7.092654744	0.000169609	hsa-miR-132-3p	122	2.604698753
29.75	-7.264672779	3.52892E-10	hsa-miR-2392	177	2.436560488
12.75	-7.685491185	9.55755E-05	hsa-miR-2682-5p	15.25	2.296177555
7	-9.363727766	0.004482853	hsa-miR-5587-3p	10.5	2.09701497
115.5	-10.98380767	3.65399E-35	hsa-miR-345-3p	10.25	2.048232263
22	-13.27430672	4.08947E-07	hsa-miR-10400-5p	20	1.966032711
			hsa-miR-629-5p	22.75	1.965147176
			hsa-miR-410-3p	74.75	1.904860223
			hsa-miR-106b-3p	120.25	1.899130205
			hsa-miR-877-5p	62.75	1.876190509

hsa-miR-9-5p	69	1.649482716
hsa-miR-8066	59.25	1.64002017
hsa-miR-148a-3p	862.5	1.310381842
hsa-miR-182-5p	12365.5	-1.327007068
hsa-miR-130a-3p	726.25	-1.328818165
hsa-miR-29a-3p	1045.75	-1.346100479
hsa-miR-192-5p	34179.25	-1.353066897
hsa-miR-3155a	369	-1.373899941
hsa-miR-769-5p	362.75	-1.379516883
hsa-miR-589-5p	140.75	-1.389220535
hsa-miR-34a-5p	335.75	-1.395513524
hsa-miR-103a-3p	1881	-1.440104023
hsa-miR-300	75	-1.454629284
hsa-miR-128-3p	327.25	-1.470034005
hsa-miR-196a-5p	128	-1.517905524
hsa-miR-151a-5p	2481.25	-1.544890875
hsa-miR-331-3p	319.25	-1.548807819
hsa-let-7a-5p	34460.75	-1.574642609
hsa-miR-191-5p	4219.5	-1.576126093
hsa-miR-3184-5p	566.5	-1.591665565
hsa-miR-561-5p	78	-1.616011874
hsa-miR-1297	6967.25	-1.629281297
hsa-miR-31-3p	94.5	-1.630793527
hsa-miR-193a-3p	1137.5	-1.633705436
hsa-miR-190a-5p	28.25	-1.660561423
hsa-miR-99a-3p	96	-1.685729808
hsa-miR-221-3p	2559.5	-1.689672214
hsa-miR-365a-3p	238.75	-1.696477917
hsa-miR-941	184.25	-1.707178068
hsa-miR-28-3p	3228.25	-1.724482471
hsa-miR-452-5p	42.25	-1.725566946
hsa-miR-320a-3p	597.25	-1.747892258
hsa-miR-12135	409	-1.767612403
hsa-miR-28-5p	241.25	-1.779384408
hsa-miR-944	46	-1.791958058
hsa-miR-143-3p	125.5	-1.805323236
hsa-miR-122b-3p	9213	-1.806804551
hsa-miR-148a-5p	26.5	-1.826875814
hsa-miR-409-3p	29.5	-1.858406481
hsa-miR-455-3p	22.5	-1.877809806
hsa-miR-125a-5p	5732	-1.879402414
hsa-miR-574-5p	50.25	-1.881567781
hsa-miR-1296-5p	18.5	-1.912584921
hsa-miR-210-3p	270	-1.933925824
hsa-miR-3927-3p	40.5	-1.954695029

hsa-miR-21-5p	109325	-1.960311034
hsa-miR-513a-5p	19	-1.962171689
hsa-miR-128-1-5p	20.25	-1.996662345
hsa-miR-328-3p	9.25	-2.001596785
hsa-miR-3667-3p	77.75	-2.025869749
hsa-miR-320e	38.75	-2.027622307
hsa-miR-193b-5p	25.75	-2.031607287
hsa-miR-1180-3p	73.5	-2.031894006
hsa-miR-31-5p	4277.25	-2.039888477
hsa-miR-22-3p	28627.5	-2.107871049
hsa-miR-30a-5p	25622.5	-2.122476471
hsa-miR-3145-3p	14.75	-2.12585818
hsa-miR-30a-3p	450	-2.179418022
hsa-miR-10396a-5p	105.25	-2.180056037
hsa-miR-423-3p	740.5	-2.200908026
hsa-miR-4536-5p	21	-2.220098319
hsa-miR-374a-3p	214	-2.225178794
hsa-miR-224-5p	111	-2.251200314
hsa-miR-500a-3p	33.75	-2.253021345
hsa-miR-5579-3p	288	-2.330844707
hsa-miR-671-5p	9.75	-2.336559084
hsa-miR-134-5p	33.75	-2.360783525
hsa-let-7f-2-3p	12.25	-2.405040757
hsa-miR-10a-3p	13	-2.441494106
hsa-miR-6734-5p	11.5	-2.480284689
hsa-miR-221-5p	20	-2.504449184
hsa-miR-10396b-5p	16.25	-2.600996806
hsa-miR-26a-1-3p	9.25	-2.646617195
hsa-let-7a-3p	84.75	-2.649634701
hsa-miR-6499-3p	6.25	-2.67009498
hsa-miR-6847-3p	9.5	-2.72085572
hsa-miR-6774-3p	10.75	-2.720975419
hsa-miR-141-5p	5.75	-2.721440732
hsa-miR-744-3p	5.75	-2.721442084
hsa-miR-6850-3p	8.75	-2.724818531
hsa-miR-3122	12.75	-2.745708652
hsa-miR-887-5p	8.5	-2.802121404
hsa-miR-374b-3p	10	-2.861438907
hsa-miR-3909	6.25	-2.952035382
hsa-miR-6741-5p	59	-2.985050735
hsa-miR-582-3p	573.25	-2.985479123
hsa-miR-605-5p	7.5	-2.994762595
hsa-miR-3978	20.75	-3.000026599
hsa-miR-3165	25.75	-3.011498759
hsa-miR-424-3p	32	-3.013263805

hsa-miR-1257	80.75	-3.076535996
hsa-miR-6752-5p	4.5	-3.145294913
hsa-miR-181a-2-3p	418.5	-3.226645718
hsa-miR-4446-3p	55	-3.242970085
hsa-miR-1587	4.75	-3.315167681
hsa-miR-6844	5.75	-3.453557472
hsa-miR-4280	5.75	-3.454916646
hsa-miR-4502	29.75	-3.47655896
hsa-miR-3662	43.25	-3.492804486
hsa-miR-1273h-5p	8.25	-3.501938522
hsa-miR-1272	46.75	-3.593042878
hsa-miR-210-5p	13.75	-3.679272052
hsa-miR-125b-1-3p	20.75	-3.722756519
hsa-let-7e-3p	17.5	-3.758369957
hsa-miR-4716-3p	8.25	-3.890023959
hsa-miR-30c-1-3p	47.25	-4.067681857
hsa-miR-181a-3p	209.25	-4.270415582
hsa-miR-6850-5p	5.25	-4.324649297
hsa-miR-4303	5.25	-4.32515007
hsa-miR-671-3p	29	-4.45933078
hsa-miR-194-3p	12.75	-4.513186171
hsa-miR-584-3p	3.5	-4.626785585
hsa-miR-4697-3p	3.5	-4.627120172
hsa-miR-34a-3p	4.75	-4.810853417
hsa-miR-6747-5p	6	-4.925532593
hsa-miR-6881-5p	7.5	-5.175835324
hsa-miR-3940-5p	22	-5.5118804
hsa-miR-365a-5p	12.25	-5.721322478
hsa-miR-25-5p	13.75	-5.794629661
hsa-miR-23a-5p	29.75	-5.800309829
hsa-miR-9900	149	-5.867455772
hsa-miR-125a-3p	43.75	-6.07404552
hsa-miR-7974	100.75	-6.836231045
hsa-miR-6758-3p	3.75	-6.979265175
hsa-miR-100-3p	21	-8.052426202
hsa-miR-6125	9.25	-8.760242985
hsa-miR-29b-1-5p	9	-8.984354109
hsa-miR-27b-5p	343.75	-9.17095664
hsa-miR-214-5p	88.75	-9.201974603
hsa-miR-4659a-5p	44	-9.400441614
hsa-miR-601	29.25	-9.522141634
hsa-miR-7158-5p	5.25	-9.661980925
hsa-miR-4425	35.75	-10.83146896
hsa-miR-4718	6.75	-12.3458122
hsa-miR-6737-5p	115.5	-12.61499195

hsa-miR-33b-3p	11.75	-15.09187341
hsa-miR-6728-5p	12.75	-23.08080419
hsa-miR-222-5p	19.75	-24.92786296
hsa-miR-27a-5p	59.75	-30.74480434

PI Ad14 v Ad14p1 6HPI Ad14

FDR p-value	Identifier	Max group mean	Fold change	FDR p-value	Identifier
0.002140831	hsa-miR-3664-5p	5	-5.964865829	0.033493107	hsa-miR-12133
1.27972E-07	hsa-miR-649	9.5	-2.653126764	0.01990159	hsa-miR-100-5p
0.00281383	hsa-miR-33b-3p	9	-2.651364662	0.020683305	hsa-miR-183-5p
2.15497E-08	hsa-miR-8066	34.75	-1.630505591	0.020220309	hsa-miR-128-3p
0.027731349	hsa-miR-5187-3p	28.25	-1.619419298	0.033493107	hsa-miR-214-5p
0.020512503	hsa-miR-183-5p	394.75	-1.48873377	1.88204E-06	hsa-miR-134-5p
6.30103E-15	hsa-miR-100-5p	3152.5	-1.430918301	8.26962E-07	hsa-miR-4714-5p
0.026631636	hsa-miR-127-3p	636	-1.261357008	0.018712706	hsa-miR-2355-5p
0.029711624	hsa-miR-192-5p	61738.25	-1.230510406	0.018712706	
3.574E-114	hsa-miR-10a-5p	21353.25	-1.224502342	0.020220309	
0.034386439	hsa-miR-31-5p	6341.5	1.257203009	0.009012972	
0.000174149	hsa-miR-28-5p	248.75	1.324011722	0.020683305	
7.37042E-14	hsa-miR-221-3p	3722.75	1.336694513	0.000194933	
2.25581E-06	hsa-miR-28-3p	5317.75	1.354301694	0.015353497	
3.04849E-19	hsa-miR-375-3p	200.5	1.376285439	0.01990159	
2.66143E-09	hsa-miR-423-3p	1086.25	1.482479239	2.62102E-05	
3.82103E-09	hsa-miR-3184-5p	1213.25	1.628278972	4.37638E-07	
1.4968E-05	hsa-miR-1827	57.75	1.636968991	0.018712706	
4.72312E-07	hsa-miR-214-5p	102.25	1.864449176	0.00022777	
2.60905E-12	hsa-miR-128-3p	458	1.899419705	5.47723E-15	
0.001361002	hsa-miR-424-3p	32.25	1.940666901	0.011407212	
0.002271266	hsa-miR-3181	38.5	2.003010296	0.009012972	
0.002613402	hsa-miR-4435	9.25	3.287985095	0.039013719	
7.43855E-05					
5.74043E-14					
9.54856E-19					
0.002889					
0.000147254					
0.000182953					
0.004913994					
2.12932E-07					
5.00138E-08					
0.000262478					
8.56487E-07					
0.000415272					
0.000397101					
0.007149017					
1.44669E-10					
1.7655E-06					
0.000861594					
9.45367E-10					

0.000198995
0.016695345
0.022072437
1.81884E-11
0.001712197
0.000175463
3.10808E-08
0.006229992
0.001078427
3.74333E-09
3.49326E-11
4.37408E-07
0.042060548
1.27711E-09
0.000928848
3.92702E-07
6.63196E-08
0.008250439
0.006669719
0.001712197
0.002506338
2.96489E-11
1.54826E-07
2.37982E-08
5.29974E-11
1.96307E-07
8.66107E-06
0.002525334
6.41377E-13
0.006419167
5.79891E-08
0.003733281
0.017789269
4.87853E-10
0.042260725
0.000693757
7.48556E-09
2.18294E-06
0.002366702
0.016496021
8.6897E-06
0.005248282
0.005371948
2.6017E-08
2.53441E-07

2.59105E-05
0.030274785
0.022270148
0.022599648
4.39865E-11
6.30103E-15
4.15744E-11
0.006700858
0.030425483
0.035782302
0.042940205
9.30943E-05
0.003929794
1.01291E-10
0.000163772
0.021446904
0.010949734
0.035789896
0.002045368
0.035789896
0.033989199
0.011735418
0.004495674
2.03873E-06
4.82962E-12
0.012326588
5.89543E-05
0.019516736
0.042720252
0.001643786
0.006586379
0.000973636
9.45367E-10
2.94962E-05
0.001719905
0.000805586
0.022599648
0.03200933
0.016117814
2.10967E-06
0.00455405
0.002366702
0.000722317
0.007463098
0.047300498

0.006972933
7.43163E-06
0.012510678
0.036354522
3.27811E-05
5.95651E-08
0.012357715
0.004075768
0.042720252
0.033950956
0.034386439
8.75001E-06
0.047300498
0.029931395
0.018791776
0.036127699
0.000244383
0.005239892
0.006239066
0.002358064
1.8017E-06
0.001296398
0.022072437
0.001629931
6.37672E-06
0.00281383
0.047211757
0.004980501
0.000321012
0.002140831
5.60532E-05
0.000229079
0.008697675
0.016668714
0.02057595
4.02013E-09
1.11483E-10
0.009388309
0.035789896
0.044286186
0.013677756
0.012357715
0.001842895
0.000127624
0.007463098

0.009012233
0.045549159
0.022755879
0.027267948
0.012354374
0.00765963
0.013499172
0.007536917
0.01385976
0.030425483
0.009736445
0.001376012
0.047879888
0.004254398
0.013058147
0.00011828
0.004320653
3.09731E-05
4.58109E-05
0.000776606
0.007463098
6.05261E-06
0.014905808
2.62627E-06
0.029949595
0.003302219
1.19203E-06
4.83346E-05
6.13024E-05
1.99767E-06
0.030477454
2.25581E-06
1.31308E-05
7.93463E-05
0.015333471
0.001572763
5.73348E-08
0.011949783
0.006306097
0.022123613
6.12555E-10
0.004913994
0.025623894
1.09349E-06
0.004948749

1.00001E-11
0.016496021
0.011575326
0.045549159
0.00267913
0.002610845
0.00281383
0.001039747
4.15744E-11
4.74654E-13
2.7665E-14
0.04594627
3.31484E-10
8.60579E-07
5.00138E-08
0.005464114
4.12746E-08
1.63871E-06
4.78042E-05
2.96489E-11
0.044246754
3.2836E-05
0.007526684
0.009115041
0.017968027
0.00455405
0.005239892
0.02057595
1.63871E-06
0.04704758
0.013710034
0.008706378
0.04594627
0.038788936
0.035789896
0.002409609
0.021398053
0.00302575
0.031699447
5.75088E-07
9.74034E-20
0.046073395
0.001532472
3.68715E-05
2.19972E-07

1.79034E-07
0.045764551
7.42508E-16
1.79034E-07
0.034791215
0.023088116
0.02534038
1.56016E-06
8.45399E-08
0.00624954
5.09602E-10
0.000174149
5.60043E-06
3.68569E-05
0.010085611
5.39108E-08
3.70828E-25
0.016378425
0.011575326
1.03031E-08
2.86936E-05
0.04078839
0.04078839
0.013570908
0.006402449
0.00281383
4.86478E-06
1.37658E-05
8.0377E-06
1.94127E-09
1.11882E-25
1.06721E-13
2.13561E-16
0.024980803
2.46572E-08
0.001186036
0.000246112
2.69738E-42
4.64993E-15
1.91652E-15
5.29974E-11
0.004948749
1.04816E-12
0.001629931
1.71166E-23

2.29882E-05
4.58109E-05
4.22271E-07
1.49202E-17

v Ad14p1 12HPI

Max group mean	Fold change	FDR p-value
41.75	-2.757765809	2.2951E-05
3794.75	-1.720162935	0.000309963
429.75	-1.557962488	0.000309963
389.25	1.479834716	0.009429037
114.25	1.497393678	0.045811723
42.75	1.865288605	0.00995941
27.75	1.905740845	0.010901471
13.5	2.510544794	0.026976219

Ad14 v Ad14p1 24HPI

Identifier	Max group mean	Fold change	FDR p-value
hsa-miR-668-5p	58	-7.801974142	3.17337E-15
hsa-miR-6741-5p	19	-2.784928446	0.003802427
hsa-miR-7158-3p	36.75	-2.387925819	3.45142E-05
hsa-miR-4714-5p	85.75	-2.29315216	1.15776E-09
hsa-miR-9900	58	-2.061467729	1.06965E-05
hsa-miR-12133	50.75	-1.705700608	0.009700209
hsa-miR-7974	85	-1.602602726	0.000904
hsa-miR-183-5p	406.25	-1.33086895	0.035649421
hsa-miR-3155a	210.25	-1.276941705	0.042450099
hsa-miR-1297	4804	1.22072751	0.022948938
hsa-miR-128-3p	308.25	1.250976584	0.034935898
hsa-miR-132-3p	95	1.427391348	0.034935898

Ad14 v Ad14p1 36HPI

Identifier	Max group mean	Fold change	FDR p-value
hsa-miR-7158-3p	32	-4.505574034	8.93838E-08
hsa-miR-589-3p	21.25	-3.482885422	0.00037624
hsa-miR-487a-5p	15	-3.385319589	0.005580633
hsa-miR-4714-5p	55.75	-3.262114064	8.9574E-10
hsa-miR-6741-5p	17.75	-3.083226431	0.002747215
hsa-miR-1827	56.75	-2.883077547	8.93838E-08
hsa-miR-548u	14.75	-2.424916241	0.043280537
hsa-miR-140-5p	28	-2.041263118	0.010262679
hsa-miR-668-5p	45.75	-1.713866766	0.012409825
hsa-miR-3156-3p	36.25	-1.696156657	0.025539276
hsa-miR-4286	144.25	1.580506861	0.002351945
hsa-miR-3128	51.25	1.827164438	0.000151355
hsa-miR-1276	16.5	2.568626294	0.005580633
hsa-miR-649	8.75	2.857521473	0.019607293
hsa-miR-518a-5p	34.75	2.937399531	4.81607E-06

Ad14 v Ad1

Identifier	Max group mean
hsa-miR-499b-5p	15.5
hsa-miR-4762-3p	25.25
hsa-miR-3168	86
hsa-miR-134-3p	43
hsa-miR-4696	20.25
hsa-miR-4754	10.5
hsa-miR-5089-5p	52
hsa-miR-6877-5p	12
hsa-miR-3154	11.25
hsa-miR-1268a	8
hsa-miR-665	33.5
hsa-miR-412-3p	7.5
hsa-miR-6736-3p	5.25
hsa-miR-6079	9.5
hsa-miR-4654	38.75
hsa-miR-5100	21
hsa-miR-4424	9.5
hsa-miR-6085	7.5
hsa-miR-6762-5p	10.5
hsa-miR-219b-3p	7
hsa-miR-5584-5p	9.75
hsa-miR-6833-5p	6.5
hsa-miR-4462	20.75
hsa-miR-330-3p	27.25
hsa-miR-1238-5p	8.75
hsa-miR-218-1-3p	24
hsa-miR-6753-5p	7
hsa-miR-4433b-3p	5.5
hsa-miR-6892-5p	9
hsa-miR-6165	12.5
hsa-miR-6783-5p	20.25
hsa-miR-4318	13
hsa-miR-1908-3p	15.25
hsa-miR-4759	11.5
hsa-miR-551a	31.5
hsa-miR-1296-3p	11
hsa-miR-3928-3p	22
hsa-miR-4440	6.5
hsa-miR-3917	8.5
hsa-miR-642a-3p	11.75
hsa-miR-4650-3p	16.75

hsa-miR-3911	8.75
hsa-miR-4260	24
hsa-miR-5587-3p	10.5
hsa-miR-8077	18
hsa-miR-6757-5p	12.75
hsa-miR-345-3p	10.25
hsa-miR-520g-5p	32.75
hsa-miR-4431	28.25
hsa-miR-196a-1-3p	23.25
hsa-miR-8080	38
hsa-miR-4724-5p	37.5
hsa-miR-12120	74.75
hsa-miR-12136	40.75
hsa-miR-4436a	10
hsa-miR-518d-5p	32
hsa-miR-1253	81.25
hsa-miR-646	65.5
hsa-miR-7850-5p	13.25
hsa-miR-12113	19.5
hsa-miR-6784-5p	30.5
hsa-miR-596	28.75
hsa-miR-492	35
hsa-miR-7113-3p	31.75
hsa-miR-4508	33
hsa-miR-3606-3p	81.25
hsa-miR-4286	845.25
hsa-miR-2392	177
hsa-miR-21-5p	67880.5
hsa-miR-378a-3p	867.5
hsa-miR-151a-5p	1980.75
hsa-miR-27a-3p	71095.25
hsa-miR-320a-3p	421.75
hsa-let-7a-5p	27083.75
hsa-miR-34a-5p	300.5
hsa-miR-25-3p	17419.5
hsa-miR-221-3p	1947.5
hsa-miR-1307-5p	529
hsa-miR-345-5p	260.25
hsa-miR-28-3p	2501
hsa-miR-31-5p	2736.25
hsa-miR-181a-5p	18607.75
hsa-miR-22-3p	19373.75
hsa-miR-450b-5p	106.25
hsa-miR-28-5p	197.5
hsa-miR-128-3p	325.5

hsa-miR-210-3p	214.25
hsa-miR-421	131.5
hsa-miR-339-3p	61.5
hsa-miR-3184-5p	557.75
hsa-miR-106b-3p	178
hsa-miR-3667-3p	62.25
hsa-miR-423-3p	561
hsa-miR-3128	97
hsa-miR-518a-5p	93
hsa-miR-1276	41
hsa-miR-432-3p	16.25
hsa-miR-1303	5

4p1 48HPI

Fold change	FDR p-value
-21.32369586	0.000116902
-19.22662547	2.22568E-06
-14.44730214	0.000667838
-13.8857482	5.34888E-12
-10.62499985	2.744E-06
-10.31778036	0.001456994
-9.526474287	1.82511E-14
-9.147474116	0.000669297
-8.578034286	0.001092371
-7.900058802	0.007124941
-7.848442803	7.7885E-08
-7.40012597	0.006631442
-7.229678621	0.040615588
-7.177316788	0.005044215
-7.101235101	4.50371E-10
-6.779978959	2.03047E-05
-5.930504428	0.004096229
-5.746126869	0.010636312
-5.544228664	0.003977253
-5.335696009	0.026234875
-5.143208926	0.005475076
-4.990490964	0.01907494
-4.854172275	8.01663E-05
-4.70262946	4.97165E-05
-4.622906492	0.012130687
-4.393451515	0.00010477
-4.387781628	0.022821092
-4.235831012	0.046092102
-4.117410098	0.010381572
-4.066006593	0.002040164
-3.927738862	0.000959114
-3.860509174	0.001201102
-3.85033058	0.001064569
-3.744753777	0.006856418
-3.623853015	4.97165E-05
-3.58257512	0.010707379
-3.46330796	0.001092371
-3.449046635	0.040615588
-3.43276113	0.020600071
-3.405231484	0.012941455
-3.313746103	0.010039264

-3.159163681	0.030214328
-2.965420525	0.001171517
-2.872841916	0.01303362
-2.850003511	0.00470403
-2.807808966	0.007581805
-2.804967705	0.01633974
-2.71131562	0.000569968
-2.699381526	0.001214578
-2.678373554	0.002739051
-2.60102259	0.000158282
-2.5817398	0.002096908
-2.560261333	0.000116902
-2.417719227	0.000669297
-2.359339631	0.041702997
-2.33339882	0.000669297
-2.319940433	0.000569968
-2.295415863	0.001862114
-2.204565838	0.023390315
-2.17452363	0.033891128
-2.132303541	0.003970428
-2.054169362	0.00803314
-2.021246876	0.006996308
-1.975018647	0.004785212
-1.879757629	0.010039264
-1.775938046	0.004096229
-1.774747562	4.84052E-05
-1.657361829	0.001064569
1.313619695	0.02575761
1.341250887	0.022648599
1.345792782	0.02575761
1.348751499	0.012899168
1.352687866	0.040615588
1.353496054	0.017770006
1.369579788	0.030184929
1.386439525	0.010636312
1.414808675	0.005475076
1.418195801	0.013595954
1.439457092	0.041430516
1.445522529	0.005461007
1.45064262	0.003481963
1.547176433	0.000477427
1.558080552	0.000146972
1.600776649	0.020164164
1.609576925	0.004096229
1.626684968	0.000569968

1.662748695	0.000959114
1.663520118	0.014157646
1.692773276	0.014157646
1.735558987	0.000146972
1.773459747	0.000790428
1.820630858	0.025888489
1.90020314	8.43759E-05
2.323919321	0.000146972
2.333665516	0.000116902
2.347277167	0.000136251
2.466191275	0.010707379
4.464356826	0.030360483
