# Supplementary material for: Differential Expression of Host miRNAs During Ad14 and Ad14p1 Infection
Source: Viruses. 2025 Jun 11;17(6):838. doi: 10.3390/v17060838 (PMC12197781; doi:10.3390/v17060838)
Supplement: Supplementary file 1 [file viruses-17-00838-s001.zip › SI Table 2.pdf]

miR27a-3p	miR-21-5p	let-7a-5p	miR22-3p
TERF2	FAM102B	TERF2	AHI1
CALU	RTN4	CALU	FAM102B
BMI1	SLK	ARHGAP1	RTN4
TMSB10	RPRD2	RNF41	C17orf85
ZKSCAN5	WWC2	VPS4A	ARPC5
RNF41	TMEM16C(hsa)	ERLIN1	RPRD2
CCNT2	YTHDC2	OSBP2	COPS7A
POLR2B	CELF2	SLK	B2M
GOLPH3L	ESR1	RPRD2	ESR1
RTN4	PELI1	ESPL1	PLXNA2
VAPB	DDI2	NEK3	ATF6
SLK	PLXNA2	YTHDC2	SRRT
RPRD2	VPS13C	SEMA6A	SCLT1
ESPL1	XKR3	NDUFS2	RBM14
YTHDC2	PTPDC1	PPP1CA	DENND3
SEMA6A	KLHL28	KIAA0754	HSPA13
NDUFS2	RNF11	TRIB3	HGSNAT
CELF2	HGSNAT	RBM11	POU2F1
PPP1CA	TNFRSF11B	SLC44A2	FASN
E2F7	ADAM9	PLXNA2	CDC6
DDI2	ACTB	C5orf28	HIST1H1A
ABCB10	CCNG1	VPS13C	EIF3L
PLXNA2	TANC1	RGS17	SLC38A1
BRK1	EIF3L	MFSD12	TTC7B
PTPDC1	PRLR	PFN1	NACA
TMPRSS2	BLOC1S1	AK2	DUSP4
C12orf73	NEK7	IRS2	PRLR
KLHL28	PAQR3	TMPRSS2	IQCJ-SCHIP1
CAMK2D	MSI2	CAMK2D	XXYLT1
BRAF	IGFBP5	BRAF	TENM3
FHL2	PPP1R37	POU2F1	LIG1
HGSNAT	EGR1	ANP32E	DCP1A
ANP32E	ZMAT3	SLC41A2	IGFBP5
LAMB2	NETO2	UBASH3B	EGR1
FASN	HIPK2	CDH26	LRP10
ADAM9	CNOT6	ADAM9	ZEB2
CCNG1	SCYL3	ACTB	MLK4
TANC1	TNS1	CCNG1	PICALM
PHLPP2	FBXO11	HDX	DPP3
PRLR	KYNU	SNAP23	CNOT6
TMEM50A	URGCP	DUSP4	BICD2
NRCAM	MARCHF1	RAB4A	GSK3B
NEK7	ALS2	PRLR	RNF44

BSCL2	SLMAP	MAGI2	BRWD3
MSI2	TSC1	NEK7	MOV10
SERTAD3	PTPN3	PAQR3	ASB7
SEC16A	RBM27	SEC16A	FAM120A
DCP1A	TXNL1	PCNXL4	RBM25
IGFBP5	VWA9	EGR1	SOX4
EGR1	BRMS1L	FOS	PDPR
LRP10	ENC1	SFSWAP	ARAP2
PDLIM5	UGCG	PICALM	TRA2B
PICALM	SAR1A	CASP8AP2	SIPA1
ZMAT3	STAT3	FAM195A	WAPAL
PDK3	B3GNT5	NFE2L3	TMED9
HIPK2	CCAR2	HIPK2	CHST12
CNOT6	EPRS	CNOT6	ESYT1
TMED8	TNPO1	RPL31	RIC8A
EDRF1	GALNT7	BICD2	ZBED5
WIPF2	GLG1	MEN1	FARP1
RPL31	FERMT2	RNF44	UBXN7
CRTC3	ARFGEF1	BRWD3	NDUFB10
GSK3B	G3BP1	FAM120A	SCAMP3
BRWD3	RAB3GAP2	C5orf30	SDF2
CXADR	PDCD4	GPR63	FAM96B
ASB7	CDS1	SOX4	TRIM11
FAM120A	TGFBR1	PDPR	VWA9
RBM25	MTERF	CDIP1	POLR3D
HID1	PHRF1	SLC15A4	TBCD
PDPR	CAPN2	PAGR1	FKBP3
SLC15A4	VASH2	CCNB2	SPARC
FBXL3	DSC2	FBXO11	UGCG
FBXO11	FBXO46	RNF43	DNAJC22
HBEGF	CEP152	RBL2	GUCD1
UBFD1	SAFB2	C9orf40	STAT3
KYNU	TIPIN	LRRC47	MYO9B
IL1RAP	IRF4	DUSP22	RREB1
OAS3	S100A16	SCAMP3	THY1
PYGL	IPO11	TSC1	SIGMAR1
URGCP	SMC1A	PHF19	TNPO1
ZBED5	RPS7	ARG2	PTPRK
UBXN7	C8orf57(hsa)	RBM27	DIP2A
USMG5	UBXN4	ANKRD13B	FERMT2
DAZAP2	PTGFR	VWA9	ARFGEF1
SMG5	SPRYD4	SIN3B	SWAP70
SCAMP3	MEGF9	BRMS1L	EEF2K
FST	VDAC1	POLR3D	TOB2
TSC1	PHF20L1	ENC1	NBAS

**CLU**	**TM4SF18**	**TUBGCP3**	**TM4SF1**
**APMAP**	**TTL**	**FKBP3**	**PRR22**
**RPA3OS**	**SOCS4**	**PCNA**	**EMP1**
**PEAK1**	**HIF-1Ã□Å½Ã□Â±(hsa)**	**UGCG**	**APEX1**
**ARL6IP5**	**WNK3**	**STAT3**	**EPHB2**
**PSD4**	**FBXL5**	**B3GNT5**	**SAFB2**
**SPARC**	**RPN1**	**MYO9B**	**FEM1B**
**UGCG**	**CEP97**	**RREB1**	**TWF2**
**EPRS**	**POLR1C**	**SIGMAR1**	**PTPRU**
**MAN2A2**	**TACC1**	**NAGA**	**S100A16**
**RREB1**	**EZR**	**UTP15**	**MET**
**SFXN4**	**YAP1**	**PIEZO2**	**MAP2**
**NAGA**	**HMGB3**	**ARFGEF1**	**SENP3**
**CLPP**	**PBK**	**ELL2**	**PHF8**
**TNPO1**	**ZBTB44**	**TOB2**	**C14orf1**
**GALNT7**	**ANKRD26**	**G3BP1**	**BLCAP**
**UBE2Z**	**KAT6A**	**MCAT**	**MEGF9**
**ARFGEF1**	**CDS2**	**RAB3GAP2**	**TTYH3**
**EEF2K**	**RNF38**	**PDCD4**	**ANO6**
**TOB2**	**ZBTB38**	**TGFBR1**	**RPL17**
**E2F1**	**GCLC**	**TM4SF1**	**OPTN**
**TMEM204**	**SOS2**	**MACC1**	**ATP2C1**
**RCC1**	**LTA4H**	**RTTN**	**ZBTB39**
**MASTL**	**MAP4K2**	**ADAM10**	**PTMA**
**ASB1**	**LSM3**	**ADAMTS5**	**EPCAM**
**TGFBR1**	**WDR47**	**PDE4DIP**	**RNF2**
**MSI1**	**FUS**	**NAT6**	**GPN1**
**PDE4DIP**	**BDH2**	**EMP1**	**SOCS4**
**CXCL16**	**PLEC**	**COX8A**	**BCL9**
**WDR1**	**ITGB8**	**CHST3**	**USP39**
**TCEB3**	**PCSK6**	**ANKRD13A**	**SNX19**
**APEX1**	**FAM126B**	**TSSC4**	**CIC**
**GGCT**	**RHOBTB3**	**LEPROTL1**	**UBE3B**
**DSC2**	**WDR7**	**IRF4**	**FAM109A**
**EPHB2**	**UBE2T**	**PDHX**	**CDR2L**
**FBXO46**	**CCDC39**	**ARID3A**	**TACC1**
**NFKB1**	**LAMB1**	**PTPRU**	**YAP1**
**FAM189B**	**PTPLB**	**FAM129B**	**LSM7**
**IRF4**	**ATF2**	**SMC1A**	**TOP3B**
**UNKL**	**DDX50**	**MET**	**SSR1**
**PDHX**	**RAPGEF1**	**UTP14A**	**ELF2**
**EXT1**	**GPR137B**	**TROVE2**	**SPIRE1**
**FEM1B**	**TPM1**	**GP5**	**DDT**
**RAP2B**	**ZNF12**	**LRBA**	**C1orf43**

EIF3F	OSR1	PHF8	GATM
PTPRU	ZNF791	YWHAH	KAT6A
SNX11	ZNF70	SRSF9	RPL32
SMC1A	EIF2AK3	SPRYD4	FPGS
TOP3A	ALMS1	SSH2	ANGEL2
MET	ZGRF1	UBP1	RNF38
UTP14A	C11orf95	TOM1L2	FUS
MFSD1	RNASEL	C20orf24	PLEC
TROVE2	TDRD1	CSK	NELFCD
UBXN4	SCRN1	GPR85	PCSK6
SRSF9	DICER1	CDT1	PGR
SIPA1L3	DOCK3	TTYH3	RHOBTB3
CBFA2T3	DNM2	PPP4C	TM9SF1
BLCAP	RCN1	TOR1A	TOR2A
CNOT11	AMFR	ATP2C1	DDX50
MEGF9	NRAS	ZBTB39	FLNC
SSH2	UGGT1	ZNF691	RAPGEF1
RRAGD	LTN1	ARL8A	NUCKS1
ID2	PRKAA2	NUP62	WWP2
CSK	ALG11	GPN1	FGD5
VDAC1	IP6K1	UBR1	RAPGEFL1
TTYH3	CRKL	MED13L	HIST1H1D
ITGB1	ATXN2L	MAGEB1	RFX5
PPP4C	MAP1S	PLCXD1	PREX1
RPL17	WHSC1L1	BCL9	ATN1
TOR1A	CHCHD3	SNX19	FAM83G
TTL	TET1	RPF1	HDGFRP3
ZBTB39	LPIN1	FGD6	RAD23B
GPNMB	SLC7A6	UBE3B	GNG11
WARS2	PRRC1	STOX2	SH3PXD2A
NUP62	TSC22D3	FBXL5	PKP1
UBR1	CD38	TACC2	KRT8
MED13L	PIGX	EZR	CAMSAP1
RAB35	BID	YAP1	ABCA2
SRR	FILIP1L	ZBED4	GEMIN4
SNX19	BTRC	ADRBK1	CBL
RPF1	AP3S1	WASL	USP36
FGD6	DOCK2	DDHD1	C11orf95
CIC	HIST1H2AE	EIF2AK1	INTS10
STOX2	SKIDA1	ZBTB44	SCRN1
RPN1	TGFBRAP1	AREL1	DICER1
GNA12	SPPL2A	C1orf43	ARHGAP11A
PET112	CYB561D1	PACS1	DNM2
TACC2	NT5C2	ZDHHC23	RCN1
EFS	ZNF532	GATM	FBXO21

ZFAND5	EXOC5	KAT6A	PLEKHM2
TTC39C	DCAF8	MRS2	EMC10
YAP1	RFFL	LTA4H	AMFR
ADRBK1	TCF4	RB1CC1	PTBP2
NDFIP2	HNRNPA3	DNAJC6	BCAT2
FOXN4	NBPF1	RIPK2	CENPT
ZNF621	KLHL8	PKN3	NRAS
ZBTB44	ZNFX1	PEX19	IGF2BP2
SSR1	HIP1	THEM6	LPHN2
ELF2	YOD1	PLEC	SIK3
AREL1	WARS	PCSK6	ADORA2B
C1orf43	ZNF10	TCERG1	NDRG3
GATM	APC	PWWP2A	PANK4
ZIC2	RRN3	RHOBTB3	PTPN23
KAT6A	BRD8	CDK4	ATXN2L
ANGEL2	KIAA1598	JMJD4	ARHGAP19
RNF38	GATA3	GPHN	CD59
ZBTB38	CFL1	ZCCHC9	CNOT4
LTA4H	COL12A1	PTPLB	DGKE
ZNF827	UBR5	ATF2	RASA2
RB1CC1	UHRF1BP1L	LDB2	PPP2R3A
DNAJC6	TRIAP1	INHBC	SLC7A6
IL17RA	AFTPH	BLM	YWHAE
ACSL3	ARMC8	IARS	TRIM52
ALPK3	THBS1	KCTD12	CRIM1
KSR1	HSPA4	PREX1	INPPL1
LSM3	IL1B	ZNF12	ELK4
MCM9	TOR1AIP2	WIPI1	COTL1
TRAPPC11	GTF3C4	KDM5C	CLIC4
PLEC	ZNF780B	POU3F2	PEX26
FAM126B	PHACTR2	ARHGAP27	FILIP1L
LAMB1	ZMYND11	PLEKHO1	BTRC
SAR1B	IL12A	ZNF585B	CYR61
TOR2A	CMBL	KCNN4	SEPT8
ATF2	LGALS14	CAMSAP1	CCNL2
LDB2	SEPT10	ABCA2	CCNA2
NUCKS1	PRRG4	GEMIN4	SEL1L3
BLM	TTLL12	GARS	KIAA0195
HIST1H1D	CUL2	CBL	CCT7
KCTD12	PAK2	SCRN1	RMI1
PREX1	ATMIN	DICER1	AGAP3
ZNF12	RPSA	FRMD4A	BTF3
CCDC92	C11orf30	ACOX1	SCARA3
CTNND1	EDEM2	C19orf55	GPATCH8
CHCHD1	FAT3	CBX2	DCAF8

SH3PXD2A	MAP2K7	PHB2	FBXO30
BNIP3	SAMD8	LZTS3	ZNF394
MRPS35	REST	TRIM27	CALM3
PKP1	PGLS	PLEKHM2	CALM1
KRT8	PITHD1	SMUG1	ZMYM4
ZNF70	CEBPZ	ZBTB10	RGL3
ALMS1	LRRC8B	MAST4	TCF4
USP25	B4GALT5	C15orf41	ESRRG
GARS	GAPDH	SLC37A3	HNRNPA3
BRD2	MPDZ	HCN2	NUP153
CBL	MCM6	NRAS	TMEM30B
C11orf95	MED21	IGF2BP2	LGALS3BP
SH2B3	FKTN	H2AFX	MAP3K3
BCL3	PLAGL2	DIEXF	ZNFX1
HNRNPA1	NF2	SCAF4	INHBB
DICER1	ZYG11B	DUSP2	NME4
CTR9	CA5B	CRKL	GNAS
ARHGAP11A	USP1	TMEM194A	WARS
CBX2	GSTO2	NDRG3	ZNF10
CAMK2G	ANTXR1	PTPN23	PPAT
DRG1	WNT5A	ATXN2L	ALDH16A1
DNM2	RFXDC2(hsa)	C6orf136	VPS13D
CSRP2BP	DLG1	HN1	SMTN
EN2	RSF1	MTHFD2	PSMA6
TRIM27	PURA	WHSC1L1	MAP1LC3A
RBM12	HSH2D	ATXN1L	CC2D1A
ZBTB10	ZNF275	CNOT4	SIRT1
PTBP2	SEPT11	STK4	PTPN2
CBFA2T2	XRCC6	LPIN1	MED14
LAMB3	PAFAH1B1	ZNF124	THBS1
MMGT1	DENND1B	SLC7A6	IPO9
NRAS	HIST1H1E	YWHAE	HMGA2
PDK4	ARHGAP24	HDAC3	TOMM7
SMIM21	NAP1L1	TSC22D3	CENPM
UGGT1	KDM4B	CD80	TMEM101
LTN1	SOWAHC	INPPL1	GTF3C4
CTB-50L17.10	ARL2BP	REXO1	PHACTR2
ADORA2B	NUBPL	TM7SF2	POLD2
HIST1H1C	PWP1	ELK4	CDC25B
ARMCX1	ARHGEF12	COTL1	HDAC7
ATXN2L	FLI1	BID	CYC1
HN1	ZNF286A	CLIC4	SKAP1
MAP1S	FAM46C	BTRC	AGO3
WHSC1L1	SERPINB2	MMS22L	DBN1
ATXN1L	CD83	PRR3	P4HA2

STK4	CPS1	CHD1	KIAA1522
DGKE	CCND2	ABT1	DHCR24
MAP2K2	PSMD2	8-Sep	NINJ1
VPS18	ANXA1	CCNA2	IMPDH1
TPCN1	NUDT21	GNS	NPLOC4
PPP2R3A	CORO2A	AGAP3	LENG8
ZNF124	FOXM1	NT5C2	RPSA
YWHAE	SMARCA2	ZNF532	C11orf30
TSC22D3	DOCK6	MBTPS1	ANKRD52
CRIM1	RABGAP1	SCARA3	PRRC2B
FEN1	RAB6A	GPATCH8	SPATA2
ELK4	HOXA9	SIMC1	MTUS1
TMEM59	LSG1	ALDH7A1	REST
MORC2	TTC33	ATP11C	GBA2
BID	RC3H2	ZNF710	LMNB2
CLIC4	AUTS2	HOXA1	LRRC8B
HOXB4	SNX30	ERO1L	B4GALT5
TIMELESS	SEMA5A	ECE2	SEL1L
PRR3	LMBR1	CALM3	SLC35B1
KBTBD2	U2SURP	CALM1	ZNF431
CHD1	EIF5A	NBPF1	RBMXL1
CCR3	ROCK2	NUP153	NPAT
FAM195B	TESK2	MAP3K3	USP31
KLHDC2	ZNF148	KDM2B	COPS7B
SPPL2A	AKAP9	NME4	MCM6
TMED7-TICAM2	NKTR	YOD1	PLAGL2
TMEM39A	PITX2	SLC25A3	TUBA1B
RCAN1	SKA2	SOX11	TMED3
CCNL2	OTUD6B	CRK	PIK3R4
CCNA2	MYH10	SMTN	SSSCA1
GNS	KLF9	ORC2	FUCA2
SEL1L3	DPP8	NSRP1	AP2B1
FAM78A	PTPN14	SIK1	CHD8
BTBD1	SYPL1	MED14	GPAA1
ZNHIT2	ZFC3H1	THBS1	COL4A5
PPM1K	BACH1	MEF2BNB	GCA
EXOC5	SAMSN1	TMEM167A	ODF2
LPCAT1	GNG12	JAM3	ZNF275
ANKRD30B	HUWE1	MTMR6	MARVELD2
MAGEF1	FRS2	HMGA2	SEPT11
ZBTB17	CBX4	CPSF7	NRG2
FHL3	TRIM7	TOR1AIP2	PAFAH1B1
ATP11C	SNRK	TMEM101	ACSF3
ERO1L	STXBP5	ATF6B	OAF
CALM3	TPCN2	SUPT4H1	PITPNA

DCTN5	BUB1	MED12	RRP9
LRRC61	FICD	LGR4	STXBP4
ZMYM4	ANKRD50	ZNF267	MAZ
RBM48	LCOR	CYC1	BLOC1S1
NUP153	RALA	SOX18	KLF2
MIER2	MCM4	AGO3	PVR
HIP1	HRK	P4HA2	HTT
KDM2B	ACLY	KIAA1522	ARHGEF12
GNAS	REV1	ETV3	FLI1
PCLO	KIAA1549	TBC1D9	SGPL1
IP6K2	TMEM167B	EIF3D	TP53I13
FAM115A	IQGAP1	NPLOC4	TRAF7
GOSR1	GAN	CUL2	YTHDF1
MKL1	ARHGAP32	FBXW5	YWHAG
WARS	WNK1	RPSA	SEC23A
TMED7	RUFY3	EDEM2	MCL1
IWS1	GPATCH2	TPR	DUSP6
SMAD9	PHF20	FAT3	VWDE
LAMA5	PBX1	ANKRD52	TBL3
BRPF3	NBR1	PRRC2B	RAB7A
PPME1	SETD1B	MOB3A	PSMD2
BRD8	PREPL	PIK3IP1	MRPL34
HMGCR	HSPA5	THBS2	RNF14
SOX11	SLC17A5	WDR41	ACTG1
CRK	MGA	SPATA2	PPIL1
BEND5	SNRNP48	MTUS1	TSPAN3
CUZD1	SOD1	REST	FTSJ3
VPS13D	SMEK1	ELK3	NHP2
FBXW7	TPRG1L	UAP1	SATB2
DHX29	LSS	PPP2CA	PXN
SSH1	CCDC93	TPP2	FKBP14
GATA3	SMC5	XK	ASCL1
TRIM37	TMEM64	CAV1	RC3H2
COL12A1	LIMCH1	INTS1	MERTK
UBR5	VPS36	IL18	STK38
TRIM24	KIFAP3	SMARCA4	EHD4
ARF3	NUP93	B4GALT5	MTMR4
WSCD1	CCT5	SEL1L	INPP5E
SHC1	ZNF664	RBBP9	HNRNPF
NDUFAF6	ARL14EP	CPEB4	TACC3
AFTPH	BCL11A	NPAT	ARPP19
ARMC8	PTBP1	UBIAD1	ZNF148
ARRDC4	NT5E	EXTL3	AKAP9
NR2F6	APPBP2	TIMP4	NKTR
SIRT1	OSER1	SPTLC2	OTUD6B

**SIK1**	**SRGAP1**	**MCM6**	**PTTG1IP**
**HOXB8**	**MAP2K3**	**MED21**	**HM13**
**MED14**	**FOXK1**	**FKTN**	**DYNC1H1**
**THBS1**	**UQCC2**	**TCF7L2**	**RNF31**
**STOM**	**SPG11**	**PLAGL2**	**RPS6KC1**
**NLN**	**MYH9**	**ZBTB7B**	**ARHGAP17**
**RUNX1**	**HBP1**	**DIDO1**	**BACH1**
**TMEM167A**	**MED13**	**MTX3**	**GUK1**
**IPO9**	**BIRC5**	**NF2**	**PKM**
**JAM3**	**TUBB**	**NIRF(hsa)**	**HUWE1**
**ZNF521**	**C12orf66**	**NDOR1**	**NTRK3**
**RBM18**	**SLC31A1**	**USP1**	**MAP4**
**USP6NL**	**MACF1**	**ACVR1B**	**FRS2**
**VANGL1**	**PTPN21**	**RBM28**	**SNRK**
**VPS8**	**NADK2**	**TSC22D1**	**ITCH**
**CENPM**	**SNTB2**	**LRRC8A**	**BCL9L**
**NUP62CL**	**PAPPA**	**UBQLN1**	**STX3**
**GTF3C4**	**ATP11B**	**NR6A1**	**SERINC5**
**ZNF780B**	**SLC16A10**	**XRCC1**	**RBFOX2**
**PHACTR2**	**ERG**	**CSNK1G3**	**MKRN2**
**PIK3CB**	**FAM83F**	**PURA**	**MEX3A**
**CDC25B**	**PER3**	**MYO5B**	**LTBP2**
**TRABD**	**HIPK3**	**IST1**	**ATXN7L1**
**HDAC7**	**CCR6**	**11-Sep**	**ANKRD50**
**ZMYND11**	**APH1A**	**PAFAH1B1**	**ANGPT2**
**SRSF5**	**PAPOLA**	**OGT**	**CDCA8**
**PYCR2**	**PPP6C**	**RPS15A**	**MCM4**
**AGO3**	**CCDC14**	**IFITM10**	**SETD3**
**DBN1**	**PLD1**	**LANCL1**	**RNF150**
**PHF14**	**ATP6V1D**	**MAZ**	**ACLY**
**SASH3**	**CAND1**	**NAP1L1**	**C7orf50**
**P4HA2**	**RAVER2**	**BLOC1S1**	**SLC6A8**
**NCOA7**	**AGO2**	**PVR**	**ACAN**
**TBC1D9**	**TMX1**	**HTT**	**TOB1**
**PAXIP1**	**AFF4**	**ARL2BP**	**KIAA1549**
**IMPDH1**	**ANKRD17**	**ZNF200**	**STARD8**
**GLRB**	**STAG2**	**SZRD1**	**UBTD1**
**AMPD3**	**MBTPS2**	**YTHDF1**	**TRIP11**
**SMIM8**	**CSDE1**	**LMBR1L**	**ASXL1**
**ATAD2**	**DHX30**	**NEDD4L**	**WNK1**
**LYAR**	**AXIN1**	**YWHAG**	**RUFY3**
**TMEM248**	**RBM22**	**PDZD2**	**PARK7**
**PRKCI**	**FBXL2**	**P2RX5**	**PBX1**
**C11orf30**	**USP13**	**PTPN4**	**C16orf46**
**EDEM2**	**MIB1**	**DUSP6**	**NBR1**

HLTF	PRDM11	CD83	KDM3A
C10orf54	LMNB1	CPS1	ARID1B
KIAA1211L	MTMR9	LRRC20	ZNF562
AKIRIN2	HMGB2	POMT2	SRCAP
TPR	SP3	CDK2	ITGA5
ANKRD52	TOMM70A	CCND2	RAF1
PRRC2B	ATP1B1	ADAT1	FNBP1L
NSUN5	TECPR2	TYK2	DCAF5
SAMD8	ARID4A	FAM35A	SMAD3
PVRL2	NA	BMP5	PALD1
TNXB	ZNF292	DNAJB11	SNX10
THBS2	TNFAIP3	COL27A1	BPTF
SPATA2	CCSER2	ACTG1	TMEM43
MTUS1	ZNF587	TSPAN3	CIT
REST	ERF	CD74	SMARCAD1
ELK3	WEE1	FOXM1	SF3B1
UAP1	AIM1	FBXL14	CCKBR
TRAPPC2P1	ZSCAN30	ETS2	BCL11A
SETD7	SFT2D2	RALGAPA2	PTDSS1
PITHD1	RCOR1	ZNF260	NT5E
LMNB2	MSL2	ATP6V1F	ERLIN2
TPP2	IGF1R	EEA1	GFOD1
CAV1	CD164	PXN	AFAP1
CEP128	OSBPL3	MARS	LDB1
LRRC8B	CENPQ	HOXA9	SULF1
GTF3C3	RPS27A	TOMM20	ZNHIT6
B4GALT5	ACBD5	RC3H2	TMCO4
SEL1L	FMR1	ORC1	TMED5
ZNF431	DDX17	GLI2	CENPW
KCNN3	DOCK11	SLC25A32	SRGAP1
CPEB4	DSE	SNX30	FOXK1
GAPDH	BTBD3	PSMB7	CD70
ADAR	SRPK2	IFNLR1	NAP1L4
SUGP2	TSHZ3	EHD4	MYH9
USP31	CSTB	MAPKAP1	GOLGA2
DPF1	KPNA2	HNRNPF	STYX
SPTLC2	AGO4	TACC3	ZC3H7A
AFF1	KLF7	TRIM35	BIRC5
MCM6	ZFHX3	HIF3A	TUBB
PLAGL2	TPX2	GMFB	CUL1
ZDBF2	TMOD3	U2SURP	MACF1
DIDO1	LRRIQ2(hsa)	MYOF	CCNF
ZNF346	CLTC	ARPP19	ADNP2
WFS1	KPNA4	BDKRB2	PHACTR4
MTX3	SPCS3	ZNF551	SQLE

PIK3R4	SERAC1	GDF11	ANKIB1
TSC22D1	GLS	TALDO1	KIF13A
NR6A1	VCL	ANPEP	PAPOLA
C10orf10	USP47	NKTR	AAGAB
HHIP	IMPDH2	CMAS	ELTD1
DLG1	CTSC	STIM2	CAND1
PHGDH	VAPA	PTPN14	PXYLP1
RSF1	ZNF75D	EIF2AK4	ALK7(hsa)
TAF2	SNN	DYNC1H1	MCM5
E2F8	MAP3K4	HS2ST1	CORO7
CHD8	MAP3K1	LSM8	AFF4
PURA	TLR4	SYPL1	ANKRD17
GCA	EIF3B	BACH1	STAG2
ODF2	HSBP1	TMEM63A	UBE4A
ZNF275	NA	EFTUD2	TIMM8A
TMEM110	HN1L	HUWE1	MBTPS2
IST1	HADH	MAP4	ZHX1-C8orf76
ELN	NGFRAP1	SF3B4	MRPS16
SEPT11	VPS26A	FRS2	HNRNPA0
MBOAT7	PGM3	GLMN	OLFM4
MRPL51	APPL1	NRBF2	CSDE1
PAFAH1B1	SPG20	PCTP	DHX30
OGT	TMEM164	NOP10	AXIN1
IDS	CD2AP	BCL9L	MARCHF9
RPS15A	RAB1A	PHF1	MCFD2
IFITM10	PDCD6	STX3	MYO1C
PRPF40A	HIC2	HAND1	QSOX1
ZNF597	EIF1AX	SERINC5	HMGB2
NDUFS4	QSER1	ALG8	CLN8
NAP1L1	HNRNPU	RAD54B	TNKS2
SOWAHC	CXorf21	RBFOX2	SP3
HTT	ALDH18A1	KIF2A	TOMM70A
CGN	TSPAN31	GTF3C1	PRPF19
SGPL1	VLDLR	ZNF566	MOB1B
SZRD1	OLR1	MESDC1	ATP1B1
TRAF7	GID8	NBEAL2	STAG1
FAM46C	DCAF11	ZNF121	LRP5
APOF	ATP6V1G1	DIP2B	CDCA5
HYI	DCAF10	LCOR	TM7SF3
NEDD4L	SPTAN1	CDCA8	TECPR2
YWHAG	FOXN2	GLRX3	YWHAB
PPP1CC	IPO7	SLC25A25	SCAND1
MCL1	DDX3X	ACLY	ZNF292
DUSP6	PARP9	SLC6A8	DAD1
ZDHHC20	IGF2BP1	ZNF24	SLC25A5

**MAPK14**	**FLAD1**	**PGPEP1**	**DTNB**
**DUSP15**	**SNRPD1**	**RDX**	**TUBB6**
**KIAA1191**	**ECI2**	**IFT122**	**FAM83H**
**NUP133**	**FSTL1**	**ZFP36**	**WEE1**
**GNA13**	**MARK3**	**KIAA1549**	**CDK1**
**CELSR2**	**PRKCE**	**INPP5F**	**USF2**
**TNIP1**	**USP15**	**WAC**	**MLLT3**
**TYK2**	**SMARCD1**	**GAN**	**SETD5**
**BAX**	**ARHGDIB**	**NME6**	**SFT2D2**
**TBX3**	**FNBP1**	**RUFY3**	**PHF3**
**ZNF639**	**PPIA**	**TBC1D12**	**NUP160**
**HSP90AA1**	**FASLG**	**PBX1**	**AGFG1**
**TRMU**	**MTMR12**	**B4GALT6**	**NFYA**
**SMARCA2**	**SNIP1**	**RCSD1**	**RCOR1**
**ADSS**	**SRSF2**	**ZFYVE26**	**CLUH**
**RALGAPA2**	**DEK**	**KIAA1671**	**TMEM109**
**MKL2**	**CDK2AP1**	**SETD1B**	**BLMH**
**SATB2**	**GPR64**	**TAP1**	**CTDNEP1**
**PXN**	**ADD3**	**ARID1B**	**MSL2**
**MARS**	**CELF1**	**IRGQ**	**IGF1R**
**TM9SF3**	**KLF5**	**MARK2**	**TRIOBP**
**PNPLA6**	**SLC9A6**	**ZNF562**	**MINK1**
**RC3H2**	**CKAP5**	**SRCAP**	**DDX17**
**FBXL4**	**TAOK1**	**MED11**	**RAB12**
**ORC1**	**CAPZA1**	**SERPINB9**	**RFNG**
**CFDP1**	**ABHD2**	**MGA**	**PABPC4**
**TASP1**	**SMG8**	**CDH11**	**DCTPP1**
**ZZZ3**	**KBTBD6**	**CDCA4**	**TSNAXIP1**
**EHD4**	**TIPARP**	**PALD1**	**C6orf211**
**MTMR4**	**CAPRIN1**	**LONP2**	**EEF1A2**
**HNRNPF**	**CNTRL**	**C14orf169**	**KPNA2**
**GMFB**	**RFX7**	**BPTF**	**ZYX**
**ROCK2**	**ZBTB20**	**FBXO45**	**ZFHX3**
**FAM198B**	**HK2**	**TMEM138**	**ARF1**
**ARPP19**	**VPS13A**	**LIPH**	**PTGR2**
**NABP1**	**ABI2**	**PKD1**	**PRR5L**
**VGF**	**NCOA3**	**CHUK**	**RPUSD1**
**KATNBL1**	**PKNOX1**	**NHLRC3**	**CLTC**
**ZNF148**	**HAUS3**	**HOXD11**	**ZNF580**
**SKA2**	**CHM**	**MAP4K3**	**MAD2L1BP**
**PTTG1IP**	**AGPAT5**	**VPS36**	**NOTCH2**
**NRBP1**	**LPP**	**SMARCAD1**	**PTMS**
**CEP78**	**DUS2**	**KIFAP3**	**SAPCD2**
**LMBRD2**	**ATP2B1**	**GMNN**	**DAZAP1**
**SH3BP2**	**ANLN**	**ATPAF1**	**VCL**

PTPN14	PCDH17	PIGM	PSKH1
DYNC1H1	DDX39A	SF3B1	PTPRF
ADCY3	PLEKHA8	ZSCAN22	C6orf48
VARS2	NCL	ARID3B	CTSC
HS2ST1	PHTF1	LDB1	KLF11
ARHGAP17	RPLP1	DBF4	CNIH4
PKM	QSOX2	TNFRSF11A	WWTR1
GNG12	MYCBP2	SELT	IKZF4
EFTUD2	ZGLP1	ECHDC1	ABHD14B
MAP4	MPP5	TMED5	ARFGEF2
SF3B4	TC2N	RNF111	TIMP2
AGRN	MRFAP1	FOXK1	NSMF
FRS2	NHP2L1	NDST2	ZNF768
SNRK	ATXN10	OSTF1	SPTBN2
CTC1	SOCS6	CEP120	HN1L
NPAS3	CBFB	MYH9	SMIM14
ITCH	BAG4	HBP1	RTKN2
GOLGA8B	TNRC6B	ZNF516	GTF2B
ALG8	SPTY2D1	MED13	CHD7
STXBP5	C6orf117(hsa)	IMMT	NDRG2
RBFOX2	EXOC4	TUBB	APITD1-CORT
KIF2A	B3GALNT1	SLC31A1	KDM2A
LOXL2	DDX21	CUL1	UBE2J1
MESDC1	PHF6	MACF1	DTD1
LTBP2	PRRC2C	CCNF	RAB1A
ZNF121	LIMS1	XPOT	KIAA0196
ANKRD50	CEP104	TMTC3	ERBB3
EVA1C	DCTN4	PAPPA	C14orf80
MDFI	ASH1L	DDX6	PSAP
LCOR	ZSWIM6	NUP98	TAB2
RBM23	PANK3	PHACTR4	FBLN1
SLC25A25	HS3ST3B1	SLC16A10	ALDH18A1
ACLY	VARS	HIPK3	HSPH1
ARHGAP31	RERE	APH1A	RPL24
SLC6A8	PURB	PAPOLA	SLC7A5
KIAA0319L	VPS13B	NR4A2	MAGED1
PAPOLG	SREBF2	AGO2	VEPH1
ZNF24	FAM53C	LIN28B	WDR6
SOAT1	RECK	IMP4	CDK6
SAMD9	CTTN	AFF4	ATP6V1G1
TOB1	ICAM1	SH3RF3	DCAF10
ZFP36	RNF149	FBXO6	SPTAN1
KIAA1549	ZNF207	HMOX2	SLC25A29
PLEKHJ1	ITSN2	EPG5	IPO7
ARHGAP32	RFC1	MBTPS2	STAT5B

WNK1	TRIP12	FOXRED1	WBP2
MNT	BCAT1	ZHX1-C8orf76	PTOV1
RUFY3	AZIN1	PRKAR1B	DDX3X
ZWINT	RALGPS2	AGAP1	PDIA6
GSE1	PPFIA4	CRNKL1	SETD2
GPATCH2	NOP14	CHST9	PARS2
PHF20	DCAF7	HNRNPA0	KIAA1217
PBX1	TMEM66	CSDE1	COPB1
CSNK2B-LY6G5B--991	RBMS1	DHX30	OXSR1
KDM3A	SEC63	TTC17	TGFB1
SPATA13	YME1L1	AXIN1	FSTL1
TPST1	TRIM41	DGCR2	CASP2
SNAI2	SLFN11	SLC38A7	NID1
METTL4	PRR14L	VCAM1	MVB12B
IRGQ	HECTD1	MIB1	BDNF
PAIP1	APAF1	LMNB1	EYA3
ZNF562	FAM217B	MEF2D	CORO1C
SRCAP	EFHD2	MTMR9	MCM7
TRPS1	FAM46A	ALDH1L2	FKBP1A
MGA	ABCD3	CHRND	CHD4
CDH11	MLLT4	CLN8	SMARCD1
FNBP1L	LAMTOR5	TNKS2	TMEM106A
DCAF5	UBA6	SP3	FNBP1
B3GNT7	FIS1	SRM	PPIA
LONP2	KRIT1	MOB1B	MTMR12
SOD1	EIF5	ZNF384	GCC1
BPTF	ARHGAP35	GOLT1B	HIRIP3
FBXO45	PTK2	ANKRA2	MSN
NET1	PTPN13	SERINC3	ASF1B
C5orf34	IDI1	ADRB2	ZCCHC2
NHLRC3	CNN2	PPP2R5D	ITGB5
TMEM64	XPO7	RNF217	WDR26
CIT	DUSP10	THRA	POLR3E
EYA4	MAVS	ZNF587	SOWAHB
C7orf43	PTGES2	ERF	QTRTD1
CCT5	ZNF667	PI4KB	NAA15
MAP4K4	STK38L	NOD1	PRPF8
SYT4	HADHA	ABCC10	ACAD9
SLC25A51	ETNK1	WEE1	CELF1
C18orf32	LTV1	PCNXL3	PDS5B
PTBP1	LCORL	RFK	SRSF7
ERLIN2	COPB2	NUP160	FBXL7
GFOD1	CYP4V2	SLC37A4	ALKBH5
PEX5	TFDP2	CLUH	EPAS1
LIN9	NCEH1	ZBTB26	RABEP1

CAT	CCDC127	MSL2	LBR
AFAP1	BRCA1	EIF4EBP1	TUBGCP2
APPBP2	ZNF217	PLXNA1	CAPZA1
PDXK	ARL1	IGF1R	AMMECR1L
SULF1	SALL3	IFT57	ZBTB45
LRP11	NUP214	CD164	CANX
U2AF2	SH3BP4	ASB13	SV2A
NNMT	LARP1	PIK3C2B	DPP9
WNT2B	KLHL42	TNFSF10	PDS5A
ECHDC1	TAF1	ACBD5	TMEM38B
TMED5	PIAS3	DDX17	ADIPOR2
MAP2K3	TRIM59	DCUN1D2	CAPRIN1
RNF111	ZFYVE16	KARS	SLC3A2
SYNJ1	CCAR1	BTBD3	B4GALT2
RNF144B	LRRC59	TLE1	C19orf43
PHB	DOCK10	ACE	MICB
CEP120	DMD	C6orf211	KIAA1109
STYX	CSE1L	PSME4	ACVR1
TPBG	NID2	KPNA2	SART1
AP4E1	POP7	AGO4	IER3
MED13	CERS2	KLF7	RFX7
MAP7D2	ANP32A	ZFHX3	OXLD1
BIRC5	OSTC	ZNF195	FAT4
CUL1	RAB11FIP2	SERPINH1	FJX1
PPP2R4	KIAA1432	SPCS3	LMTK3
MACF1	BRD7	LEFTY1	AGPAT5
XPOT	SMC4	SLC25A10	LPP
SNTB2	OSBPL11	GLS	DPM2
TMTC3	SFXN1	VCL	ASGR1
PAPPA	ZNF185	USP47	GIGYF1
DDX6	TXNDC13(hsa)	CTSC	SKP1
ANKIB1	NFAT5	GNB1	YWHAQ
PTPN1	EDC4	KLF11	FAM96A
PPTC7	APOLD1	AMOT	NSD1
HIPK3	SLC5A3	VAPA	C12orf10
KIF13A	ATP5G3	PA2G4	NCL
RPL38	SF3B3	ERCC6	ADORA2A
PAPOLA	AGO1	TXNDC11	RPLP1
PPP6C	SLIT2	BMP6	FAM222B
UBL3	RBM7	MAP3K1	UNG
CCDC14	HAPLN1	CTD-2368P22.1	SLC16A3
BOD1L1	CHORDC1	TLR4	CPSF1
DCUN1D4	POLR2A	CHTF18	ACP1
DNAJB6	HuD(hsa)	KCTD5	MPP5
AGO2	BTG2	ETF1	CCDC85B

WDR46	PHC3	ASPH	RHOV
GLUD1	FBN1	HSBP1	INSIG1
AFF4	ANKRD49	PARG	ARFIP2
IBTK	GPD1L	PRDM5	DFFA
UBE4A	PABPC1L	ZNF768	RALGAPB
ADH1B	SYBU	SPTBN2	PAQR4
UTP20	RNF4	GTF2B	CNP
AGAP1	FAM208B	MIEF1	PACSIN3
ITPKB	TOP2A	CHD7	TP53
MRPS16	CCND1	H6PD	ALYREF
PRKCD	GRPEL2	TJP1	TBC1D16
RRP7A	C11orf1	KDM2A	SAMD1
N4BP2L2	PPARA	PMP22	EMC7
CSDE1	ST6GAL1	UBE2J1	FGFRL1
IRF2BP2	FNDC3B	ZNF510	KRT18
RASIP1	C10orf12	SLC30A7	NTMT1
PRUNE2	CTNNB1	FAM13A	LITAF
MYO1C	AP3B1	NARS	LEMD3
SLC7A11	EFR3A	HIC2	FAM178A
LMNB1	SPEN	ATG16L1	PRRC2C
RHOBTB1	SNX27	QSER1	UBB
HMGB2	PTBP3	HNRNPU	CXorf36
ALDH1L2	VRK3	TAB2	C14orf119
TNKS2	GPD2	PPP1R18	FOXO6
TOMM70A	ASNS	COQ9	CEP104
PRPF19	IRF2BPL	ABCC8	GHR
MOB1B	RAB27B	HSPH1	RPL23
GOLT1B	FAM89A	UBE2I	HDAC4
MAN2A1	BOD1	EP400	C9orf69
YWHAB	TMEM56	GXYLT1	ASH1L
TMEM68	PABPC3	CDK6	MBD3
RNF217	CCT6AP1(hsa)	PLOD2	SSRP1
CCDC117	SSFA2	IGDCC3	VARS
ZNF292	RASGRP1	NOTCH4	EDEM1
TNFAIP3	LYRM2	ATP6V1G1	SPECC1L
IPPK	STRBP	TFAP2A	SH3PXD2B
THRA	ZADH2	COL3A1	HSPG2
ACAP2	SBNO1	VPS37B	CCDC97
WEE1	GIGYF2	HOMER2	SDHC
SETD5	DOCK1	C1orf21	LRPPRC
SFT2D2	INPP5K	FOXN2	SNX8
PHF3	SKP2	FYN	ZXDB
NUP160	TSPAN2	KAZN	ZMIZ2
RCOR1	SOX9	DDX3X	ARIH1
PPP2R2B	GCN1L1	IGF2BP1	SDHAF2

EIF4EBP1	PI4KA	PARS2	ADAM17
PLXNA1	AXL	SLC35A4	EEF1D
LONRF1	SAMD4A	OXSR1	DPF2
IL24	NUFIP2	FSTL1	RNF149
MINK1	POGK	FBXL16	ZNF207
ASB13	ZNF650(hsa)	MARK3	RFC1
PIK3C2B	COPS5	INIP	TRIP12
MFN2	NEK1	HERC1	MIS18BP1
DDX17	BLOC1S6	SHMT2	AZIN1
RAB12	TMEM243	GBF1	ATP9A
DCUN1D2	DYNC1LI2	CORO1C	RALGPS2
KARS	CLDN7	SLC16A9	VAV2
DNAJC5	HFE	PHF10	NPR1
AFG3L2	PM20D2	FBL	DYRK2
C6orf211	NDUFS1	MID1	ZNF317
DOC2B	E2F3	AP1M1	DCAF7
UGP2	PAIP2	GSTO1	PLCB3
KPNA2	SNAPIN	FKBP1A	CYTH3
CCDC149	SMCHD1	PREP	FRAT2
TMEM41B	DPYSL2	SMARCD1	YME1L1
ZFHX3	ANP32B	TMEM106A	RYK
HSPD1	DMXL1	FZD3	FAM43A
TPX2	YEATS4	CPD	SZT2
WWC1	TLK2	ZBTB41	CHPF2
NACC1	TARBP1	SEMA6B	HECTD1
TARDBP	PPAP2B	MTMR12	NPTX2
SERPINH1	KLHL6	MSN	NUMA1
SERPINE2	ZNF493	ASF1B	APAF1
CLTC	PAG1	FARSA	ASAP1
KPNA4	CSNK1A1	ZCCHC2	LAPTM4A
SPCS3	CREBRF	TRAM2	MTR
MAD2L1BP	SPTLC3	ZNF107	FAM69B
NOTCH2	RNF24	ACTA2	TRIM14
SQSTM1	MKRN1	EPC1	LAMTOR5
GABARAP	F2R	API5	PCGF5
ZNF800	KCTD10	CCDC71L	UBQLN2
GLS	ACTR2	NAA15	SCAMP1
ID4	FOXN3	PRPF8	SLC7A1
VCL	MYC	ACAD9	KRIT1
USP47	RMND5B	HPS3	MYO1D
WDFY2	SRPK1	SPECC1	SLC6A16
GNB1	MRPL16	FBXL7	ARHGAP35
RASGEF1B	HAX1	RABEP1	SLAMF7
ZNF75D	PBRM1	GALC	CLCN4
PA2G4	NR2C2	SLC9A6	MRPS7

GNL1	RNF185	HS6ST3	LCMT1
SNN	EPM2A	7-Mar	FOXP1
LUC7L3	RAB6C	XPNPEP1	SOGA1
MINPP1	SF3B2	LBR	SHKBP1
MAP3K4	RAPGEF6	TUBG1	XPO7
WWTR1	FBXL17	TAOK1	TIMM22
GM2A	FBXO3	ALAS1	RFXANK
CXXC1	DOCK5	TFPI	TRAFD1
SPAG17	SACM1L	ERGIC2	MAVS
ARFGEF2	MYO9A	MLH1	GNA11
XBP1	ASB6	TAB3	GTF3C2
PPP2R5C	RAD23A	DST	PEG10
COA6	ELMSAN1	KIAA2026	MLEC
EIF3B	GPAM	AMMECR1L	ARHGAP18
ASPH	DOCK8	PLAGL1	FAH
FIZ1	MMP9	FAM160A1	AP000350.10
RHOA	SERPINI1	SYNM	AMPD2
B3GALNT2	GPRC5A	RALY	MED16
PCDH10	LIFR	EPHA7	ZNF217
TMEM199	ACY1L2(hsa)	FOXK2	LARP4B
SEC61A1	SUB1	SIKE1	TRIM22
MGLL	DIAPH1	OSGEPL1	FAF2
CACUL1	PTP4A2	PDS5A	GGA3
SF1	COG5	ADIPOR2	NUP214
RTKN2	CCT8	CAPRIN1	NAA20
CHD7	CLIP4	MICB	SH3BP4
HADH	ITPRIPL2	CNTRL	FAM213B
NGFRAP1	ZFP36L1	RFX7	LARP1
VPS26A	GOLGA4	PQLC2	KLHL42
KDM2A	KLF3	NCOA3	CPT1A
PGM3	UBA1	ZNF622	HNRNPUL2
ZNF330	DEGS1	LIMD1	KIAA1147
UBE2J1	TBCEL	MTUS2	CSRP1
SNRNP27	PCBP1	EIF3K	LRRC59
NAAA	ACOT7	GIGYF1	DERL2
APPL1	MSH2	LRRC42	DMD
C18orf25	CPNE3	ATP2B1	CSE1L
TMEM164	SLC16A1	ANLN	BCS1L
CD2AP	MTPN	FAM96A	CNIH1
GALK1	PLAT	PLEKHA8	NPC2
PRSS23	SOCS5	CCR7	KANSL1
PSAP	SKIL	PREB	BEX1
PDP1	TFRC	RPS6KB2	POP7
GOLM1	BLVRB	ATP5B	MB21D1
HOXC11	CCL20	RPLP1	KBTBD4

HIC2	VIM	FAM222B	ANP32A
QSER1	FGB	PNMA2	SP4
HNRNPU	DYRK3	ZNF852	CDH1
MRPS14	TMED10	MYCBP2	KEAP1
CYLD	ATAD2B	ZDHHC3	FZD4
HSDL1	REL	UNG	NUDT16L1
PNPLA8	DMTF1	ALCAM	DDIT4
HSPH1	ATRX	SLC16A3	SFXN1
RCAN2	FKBP5	KLF10	MYH14
VLDLR	ME1	CPSF1	NFAT5
SLC7A5	ECT2	ZNF57	UBL7
OLR1	SET	CSRNP2	PIKFYVE
GXYLT1	TTC1	SMARCB1	PPP1R12A
WDR6	ZMYM2	NHP2L1	TET3
WIZ	ENPP2	G6PD	CMIP
SNX15	FAM20B	C15orf39	MRPL40
ICAM2	WASF2	ALOX5	DDX54
EZH2	HSP90AB1	USP12	EDC4
CDK6	ARHGDIA	PPP2R5A	CSNK1D
EPDR1	MYO5A	FAM104A	ZBED6
HEG1	SLC38A2	RPS6KA5	RIF1
CNTNAP2	NCOR1	CERCAM	ATF5
MAP3K9	DDX46	MFHAS1	TEX10
RHEB	EIF4G1	MAML3	AGO1
DCAF10	RAB11A	TP53	NCLN
IPO7	MIA3	SPTY2D1	EIF2A
GATA2	ANO5	EXOC4	NAV2
DDX3X	SAV1	LRIG2	CLIP1
MDH2	HDDC3	ADAMTS12	NOTCH1
SETD2	CERK	B3GALNT1	RSU1
RER1	JMJD1C	ZNF543	GANAB
TGFB1	NOL4L	LEMD3	BTG2
FSTL1	VEGFC	NUAK1	PHC3
CNNM2	TIMP3	PRRC2C	PDLIM1
MARK3	NCAPG	CDC34	ORMDL1
INIP	TRIM33	RICTOR	ARFGAP2
RPIA	HSF2	ASH1L	FBN1
MVB12B	TGOLN2	ZSWIM6	ENO1
TCEB1	CYB5R4	RBBP6	COMMD4
MCM7	AGGF1	SRSF1	NCOR2
SLC16A9	MUC1	MFSD4	SLC16A14
PHF10	ARIH2	HSPG2	EIF4H
MID1	ASNA1	FADD	PDCD10
SLC35A5	NCOA2	HIVEP2	JUN
NOL9	ZNF354C	ZDHHC12	CRTAP

**GSTO1**	**NIPAL1**	**HDHD1**	**PPP1R15B**
**CHD4**	**PIK3R1**	**HIF1AN**	**RBMS2**
**ARHGDIB**	**ARMCX3**	**PRDM1**	**WDR33**
**MBD1**	**SREK1**	**PNKD**	**DCP2**
**FZD3**	**SATB1**	**DNAH1**	**ENDOG**
**CPD**	**SKI**	**GABPB1**	**XPO6**
**FNBP1**	**ANKRD27**	**ARIH1**	**TXLNA**
**ZBTB41**	**CSNK2A1**	**LEPRE1**	**FAM208B**
**BUB3**	**MARCKSL1**	**DPF2**	**PBXIP1**
**MSN**	**POMT1**	**ITSN2**	**RSAD1**
**SLC35C2**	**DHX38**	**TMEM132B**	**TNFAIP2**
**TPM3**	**LRRC57**	**BCAT1**	**WDR82**
**RPS20**	**CD97**	**SOCS3**	**USP32**
**WDR26**	**DOCK4**	**AMD1**	**FMNL3**
**TRAM2**	**TMEM246**	**VAV2**	**RBM3**
**ZNF107**	**HSP90B1**	**ZNF343**	**JUNB**
**EPC1**	**COPS4**	**NPR1**	**GIT1**
**API5**	**GLCCI1**	**DYRK2**	**ZNF805**
**SREBF1**	**HDAC2**	**H2AFV**	**UNK**
**NDUFC2**	**NAA30**	**ZNF317**	**RHNO1**
**ADD3**	**NUSAP1**	**TEX2**	**MMADHC**
**TANK**	**PEX5L**	**DCAF7**	**ALG14**
**PRPF8**	**RGS1**	**SBF1**	**ELOVL1**
**SLCO3A1**	**TMPO**	**PLCB3**	**SPEN**
**CELF1**	**SRRM2**	**ZNF589**	**SNX27**
**PDS5B**	**TEX261**	**RBMS1**	**PPT1**
**DBF4B**	**ADAM22**	**SEC63**	**TUBA1C**
**EPAS1**	**KLHL15**	**FLII**	**MSH6**
**MED12L**	**FXR1**	**UTY**	**CTNNA1**
**LZTR1**	**DCUN1D3**	**YME1L1**	**CRLS1**
**LBR**	**SENP1**	**RIOK2**	**SNRPA**
**CKAP5**	**EEF1A1**	**HEATR3**	**MAPRE2**
**TAOK1**	**SLC26A2**	**FAM43A**	**TRIM65**
**SIK2**	**TBK1**	**SZT2**	**GFPT1**
**ALAS1**	**EPHA2**	**TRIM41**	**SLC25A13**
**TFPI**	**PRS6(hsa)**	**PSMA2**	**SMARCC2**
**ERGIC2**	**DNM1L**	**SLCO5A1**	**NR3C1**
**ALG9**	**EIF5B**	**TRANK1**	**RFTN1**
**DST**	**LARP4**	**C7orf73**	**SHROOM4**
**CAPZA1**	**CUX1**	**CHPF2**	**ST3GAL2**
**ABHD2**	**ZBTB43**	**HECTD1**	**MB21D2**
**HES1**	**ZC3H14**	**PRTG**	**PIGC**
**PLAGL1**	**SYNE1**	**NUMA1**	**SLC6A2**
**BMP8B**	**LDHA**	**APAF1**	**NRD1**
**CANX**	**FAM3C**	**PEX14**	**POLR2H**

CDR2	EIF3J	ASAP1	KCTD2
F3	SPRY4	CRAMP1L	NCOA6
SMG8	MAPRE1	FAM217B	MYLIP
ZNF746	MYH11	JPH3	NONO
SIKE1	DUSP8	GREB1L	QARS
ADIPOR2	HMGN3	ACVR2B	MAOA
KIAA1109	PEBP1	EDEM3	PNP
ACVR1	ATP6V1A	PCDH18	INPP5K
IER3	KMT2D	ELF4	NPEPPS
CAMKK2	MRPS10	CD81	N4BP3
ZBTB20	SLC25A22	OXA1L	SH2D3C
ABI2	MAP7	MTR	PRDM8
NCOA3	TUBGCP5	FAM69B	LILRB4
HDAC9	PEA15	ATP6V1B2	FAM179B
NFX1	SESN1	SLC7A1	WWC3
WDR5	CLNS1A	HMGXB3	ARHGEF37
LIMD1	ANKRD46	HCFC2	NUFIP2
LPP	RHOQ	CKAP2L	POGK
IFNAR2	CDK11A	SERP1	RING1
TMEM123	RNMTL1	PTK2	TPD52L2
LRRC42	SCO1	SLC9A3R1	IGFBP4
ATP2B1	TMED4	C8orf58	LRRC58
SKP1	SLC36A1	FOXP1	PPP1R10
YWHAQ	TNFRSF10B	NLK	MCC
LSM5	TSNAX	SOGA1	POGZ
SUPT3H	ZNF595	LAMC3	NUP107
RCN2	PIK3C2A	SHKBP1	MXD4
PCDH17	MXD1	XPO7	SACS
PLEKHA8	RPL19	DUSP10	ANP32B
PRCC	PUS7	MAVS	HIST1H2AG
TMEM11	PPM1F	PTGES2	SEC14L1
FAM222B	CXXC6(hsa)	TES	AKT1
SAFB	KIAA0247	MYO1F	UBE3A
CMTR2	TMEM2	EIF2S3	PGF
SNX14	SNRNP40	ZBTB37	ZC3H7B
MYCBP2	PSMC3	LYPD3	PARVB
ALCAM	CCDC88A	TTC26	FAM160B2
KLF10	AP2A1	GTF3C2	CSNK1A1
MPP5	CALD1	PEG10	UQCR10
TMEM181	ADNP	MLEC	CEP68
NHP2L1	HSPA8	ARHGAP18	TRMT1L
INSIG1	NF1	LRCH3	FAM134C
ATXN10	FIGN	SRRD	DLC1
ARFIP2	JPH1	COPB2	ACTR2
FAM104A	LYRM7	SF3B5	FOXN3

RPS6KA5	STRN	POLR2D	IRS4
RALGAPB	IPP	YARS2	MYC
SOCS6	MED28	ZNF217	QKI
CBFB	SUV420H1	ITGAV	TMEM209
MFHAS1	CLIC2	GGA3	CYHR1
SIDT2	CASC5	NXF1	JKAMP
MAML3	YY1	SALL3	ACAP3
CUL4A	SRP72	NUP214	PCDH1
TP53	SP1	NAA20	SON
BAG4	THRB	ZNF385A	HCFC1
TNRC6B	ARID5B	LARP1	BMF
SPTY2D1	LURAP1L	KLHL42	MAGED2
ALDH5A1	ZPR1	ABCF2	ASB8
CPPED1	JAG1	MAPKBP1	FAM83D
MOCS3	RBPJ	PGM2L1	DOCK5
MITF	ARHGAP21	CENPK	MARCHF8
ADAMTSL3	MDM4	LRRC59	PLK2
LITAF	LARS	C4orf29	NIPSNAP3A
GLTSCR1L	SLC2A4RG	PTCHD1	PPM1A
DDX21	BCL6	GSN	TUFM
PRRC2C	BTF3L4	POLR3A	ACO1
SEMA3F	SMIM13	TRAK2	CCKAR
RPL23	CDC73	ANKRD9	TP53INP1
ANXA5	NPTN	CNIH1	GBP2
C9orf69	FAXDC2	THG1L	HIST1H3D
RICTOR	BCAS2	PMAIP1	SLC4A2
DCTN4	TNKS1BP1	RNF145	MRPS2
ADAMTS2	TSEN2	CERS2	ITFG3
TOP2B	UBE2O	ANP32A	WDR11
ZSWIM6	ARRDC3	URB1	LZTFL1
EGR3	FAS	SLC9A1	POLR1B
PANK3	PGRMC1	OSTC	PGBD2
SSRP1	ANAPC5	SP4	HOXA3
PTER	NMT1	RAB11FIP2	DIAPH1
IMPA2	PRKAB2	MICU1	MLST8
RBBP6	ARID4B	PITPNM3	PTP4A2
SRSF1	LAMC1	DENND5A	SERPINB6
SH3PXD2B	PRICKLE2	KIAA1432	TMEM9B
PURB	RAI14	SMC4	CHERP
FADD	RPS6KA3	NFAT5	PSMD11
AMMECR1	TRAPPC2	PPP1R12A	NLRP1
EVL	NFE2L1	LIPT2	ITPRIPL2
HIF1AN	DXO	TET3	ZFP36L1
SLC35F5	CDK19	DROSHA	COL5A1
PNKD	LAMP2	SLC5A6	DBNL

GABPB1	TGFB2	CMIP	PLEKHA3
EEF1D	FAM160B1	NAV1	GPBP1L1
DPF2	PROSER1	CCSER1	BLOC1S5-TXNDC5
RNF40	MOXD1	ZBED6	KLF3
TMEM170B	SUN1	SLC41A3	ATXN1
ZNF207	HDLBP	LFNG	UBA1
ITSN2	DDAH1	RUNX1T1	STRAP
UROD	CBLL1	AGO1	FAM49B
RFC1	ITPA	FBXL6	LRRC32
HNRNPK	GAB1	CRYBG3	DEGS1
COX19	AHSA2	PODXL2	UMPS
TRIP12	PGRMC2	EIF2A	CLDND1
DHRS7	MGEA5	GGA2	NMD3
GRN	SLC44A1	VGLL3	TMEM86B
SIPA1L1	CKB	ARNT2	HNRNPA1L2
AKAP8L	TP53BP1	NAV2	COMT
AZIN1	GNAQ	ROBO4	TULP3
GLTP	BCCIP	SMG9	RILPL1
VAV2	PLAG1	KHSRP	YARS
DYRK2	ATXN7L3B	EEF2	CYP2U1
TEX2	NFIB	PLCG1	KANK1
RELT	REV3L	PGS1	MPLKIP
G6PC3	RASEF	RAB3IP	GPRASP2
DCAF7	TXLNG2P	POLR2A	MTPN
ZNF253	TTC22	ARHGEF2	INTS7
TMEM66	SNX29	BTG2	BIRC6
CREB1	KLHL3	CASP3	FLNB
TSG101	PITPNB	FBN1	VIM
POGLUT1	MRE11A	ENO1	DAPK1
PPP4R2	ATF7IP	NCOR2	RGS5
CCSAP	WDR3	CCDC51	BMP2K
HEATR3	ZNF281	RGS3	RPS16
DAP3	WHSC1	SLC16A14	REL
CCNT1	KAT7	CLCF1	CCDC47
ARL4C	TOPORS	B4GALT1	SLC25A24
ANKRD40	PCDHA9	ANKRD49	SRPR
ATP6V0B	SCAF11	MCM3AP	PDGFB
EZH1	CENTB2(hsa)	ARHGEF40	FRMD6
FAM122B	BCL7A	CRTAP	NDUFB7
PRR14L	HNRPH1(hsa)	PPP1R15B	ATRX
ARL8B	VPS41	PTPN6	ACTN1
HECTD1	PARP1	RBMS2	POLR2E
KLF13	GK5	CCT3	CBX6
ZNF608	CCR1	DCP2	TTC3
ASAP1	FOXP2	CLINT1	KCTD3

GALNT6	ZFYVE20	LRCH2	RPS6KL1
FAM217B	SERPINB5	XPO6	SLC25A17
LAPTM4A	BRD1	FBXO17	IRF3
DHX32	CERS6	FANCM	HMOX1
ACVR2B	C6orf151(hsa)	DHPS	SMURF1
EFHD2	GAPVD1	TMEM173	C19orf48
SDPR	G3BP2	MARS2	ENPP2
OXA1L	SOD2	DSCR3	WASF2
MTR	PAPD4	HMGXB4	GDE1
MLLT4	CEP170	RTKN	IL32
DPY19L4	TP53I11	NCAPD2	TM2D3
SLC7A1	TNS3	TOP2A	PRNP
ICE1	SUZ12	CCND1	HSP90AB1
FOXP4	ARCN1	GRPEL2	BEND3
EIF5	TRAPPC10	ASTE1	SMNDC1
SERP1	COL6A3	URM1	ARHGDIA
PTK2	PDGFD	RBMX	MYO5A
SLC2A3	SNX13	USP32	RPLP0
C9orf41	ZNF326	ERGIC3	ACVRL1
VPS4B	ESYT2	FMNL3	CENPB
MRPS7	GNB2L1	FNDC3B	DDB1
CNN2	ELOVL7	JUNB	DDX46
NLK	GALNT2	MEAF6	EIF4G1
SOGA1	LONRF2	ICMT	RAB11A
MAVS	PJA2	ZNF805	NIPA2
TIMM10	GPSM2	RHNO1	CEMIP
TMEM242	ABAT	CNOT7	FLT3LG
TES	PPP1R3B	MMADHC	PPP4R1
TICRR	SCD	ZNF678	VASH1
ZBTB37	ACSL4	ACTR3	RBL1
LYPD3	GTF2I	LPAR1	PDK1
SETDB1	KLHL9	MAPKAPK5	NOL4L
ETNK1	SDC2	B3GNT1	HNRNPM
PEG10	MYEF2	RAE1	CHTOP
PPP1R16B	PAPD7	CRY2	ZNF382
MOB3B	IL6ST	MSH6	GBAS
PISD	ZNF462	GPD2	ZNF138
LCORL	THOC2	CTNNA1	AAMP
AKT1S1	PLEKHA1	RNF25	FLNA
CBLB	FUBP1	SNRPA	C5orf51
TMEM254	KMT2A	COL15A1	TRIB2
GDA	CEP95	S1PR1	TRIM33
GPR125	AKR1A1	CCNE2	ATP11A
EML5	GOLGB1	TRIM65	TGOLN2
ZNF217	PHF16	SLC25A13	HECA

LARP4B	BAMBI	INTU	SFPQ
JOSD1	UBAP2L	TAPBP	CDC42BPA
FAF2	RTF1	RFTN1	RMDN3
NXF1	MALT1	E2F5	ARL6IP6
NUP214	GRPEL1	SSFA2	PABPC1
NAA20	UBN1	SEC24C	METTL15
PSD3	FOXO3	PHLDB2	DHX8
LIF	ANKRD28	SBNO1	ATIC
LARP1	DERL1	NCOA6	ZNF362
CEP192	GATAD2B	MPHOSPH6	PNRC2
KLHL42	ANXA11	QARS	SCYL2
PRDX4	RAB22A	USP5	AEN
TAF1	RAD54L2	UBXN8	SMARCC1
HNRNPUL2	USP7	CDV3	SKI
ABCF2	EIF4G3	PNP	TGIF1
PIAS3	TMEM30A	NPEPPS	ETV1
NCAM2	BAG6	SKP2	NR1H2
KIAA1147	RBM12B	PBX3	SEPP1
KIF3B	BRD2	ZNF264	TMCC1
ZNF697	STMN1	SH2D3C	PANX2
ARAP3	UHMK1	FAR1	DOCK4
TRIM59	CCDC34	TMEM19	FGF9
PGM2L1	POFUT1	COL18A1	PRR12
WDHD1	PKD2	LILRB4	ARPC1A
MAL2	SYNE2	SLC35C1	NPNT
DDX24	SLC25A19	NUFIP2	PHC2
FUBP3	OSBPL1A	POGK	YWHAZ
EBF3	CLSPN	WBP1L	YIF1A
C4orf29	HIAT1	RAPGEF2	YKT6
CRTC2	DOCK9	TSR1	ACVR2A
DUS1L	TNRC6A	DUSP18	MBD6
KANSL1	MDN1	SLC19A2	PSMB2
DDX18	MAP3K2	ADPGK	RGS2
PMAIP1	RSPRY1	TMEM243	GABRA6
CERS2	FAM213A	DPYSL5	TMPO
SLC9A1	USP9X	COX14	SRRM2
SP4	SAMD5	COL4A2	PLCXD3
ELAVL4	POLR3B	SLC4A7	KLHL15
MRPS18B	MBNL1	TRAK1	SLC30A9
PDHA1	PRC1	DYNC1LI2	EPHA2
FZD4	RP2	SOCS1	PIGV
KIAA1432	MON2	CCDC28B	DDX5
BRD7	SRSF11	LRIG3	LARP4
SFXN1	MYBBP1A	CNNM3	CUX1
CUL5	COBLL1	PM20D2	MIF

TRMT5	ATP2B4	GIT2	WSCD2
NFAT5	OGFOD1	E2F3	FAM219A
MFSD9	ZRANB1	MAST1	ZC3H14
PPP1R12A	PAN3	RGMB	SLC35B2
LIPT2	AVL9	SACS	RAC1
SLC35F3	MCAM	ZNF443	S100A10
MBTD1	KIF21A	ITM2C	H3F3C
TET3	EIF4EBP2	GDAP2	ANKRD13C
DROSHA	MLXIP	ACER2	RANGAP1
SLC5A6	RASGRP3	SEC14L1	POF1B
NAV1	PTX3	MAPK8	MAK16
CCSER1	HNRNPA2B1	KPNA5	CTNNAL1
KLHDC10	NAA25	AKAP8	ANKFY1
ZNF655	ASRGL1	SMAGP	CCDC58
DDX54	PFKFB2	RQCD1	CTPS2
CSNK1D	TPT1	SLC35D2	PAK4
SLC5A3	SPC24	PARVB	PEBP1
ZBED6	ZBTB1	CDK9	ABR
LFNG	OSBPL6	PAG1	NOLC1
ATP5G3	TRIM2	CSNK1A1	ATP6V1A
SF3B3	GPR155	CCR5	DNMT3A
RAB30	CCRN4L	UQCR10	KMT2D
KDELC1	TCEAL3	CEP68	ADAMTS1
KCTD21	SUGT1	DNAJC13	FSCN1
AGO1	KANSL1L	TRMT1L	UBE2K
NECAP1	DDX1	ZFHX2	THAP2
S1PR3	LIMA1	SLC29A1	TSPAN33
TBC1D4	NDUFS5	F2R	KDM6B
MICU3	CCNI	WDR48	MANSC1
AP1S2	CUL3	C1orf226	SESN1
DLX5	BTBD7	DLC1	RARA
OGDH	GID4	POLD3	CTIF
KLRG2	FAM63B	DNMT1	ZFP64
ATG101	PCBP2	HSBP1L1	HES6
YIF1B	RASSF8	MYC	BCL2L2
NAV2	AHNAK	DEDD	ANKRD46
SLIT2	TRPM7	ZDHHC6	PPP1R9B
KLKB1	NRIP1	NDUFA3	DCLK2
ROBO4	PRKDC	IFI44L	SESN2
PCNX	SASH1	p27(hsa)	PHKG2
TTC37	ZBTB34	PBRM1	HIST1H3H
PKP4	OLA1	CTC-534A2.2	TRIO
C1orf52	SMAD7	NR2C2	KIF11
STAMBPL1	UBE2W	PCDH1	CELF6
KHSRP	ZNF726	SON	FRYL

CLIP1	MKNK2	TRIM3	GMPPB
EEF2	PSRC1	SF3B2	PIK3C2A
EXOSC1	HERPUD2	CD46	OR10S1
RAB3IP	PLK1	RGL2	SURF4
BTG2	ZNF367	FBXO3	MPI
PDLIM1	JADE1	PRKAG1	C16orf72
ARFGAP2	TSN	MKLN1	PPM1F
FBN1	ARHGEF7	SMIM15	IGF2BP3
TMTC4	TGFBI	MRPS9	TMEM2
TOMM5	DLAT	PLK2	SSX2IP
GPD1L	PPARÃ□Å½Ã□Â±(hsa)	GALNT1	MGST3
PAQR7	HIST1H1B	HLA-DPA1	DHX15
ZNF711	CLCN5	MYO9A	EMP2
MFN1	DLX2	PSAT1	DYNLL2
PIK3R3	ABCC5	PIGP	CCDC50
ZDHHC2	CMTM6	HSPA14	PSMD10
CRTAP	RNF6	COPS3	ZC3H11A
PPP1R15B	MOAP1	ASB6	ADNP
WDR33	KLHL24	MATN2	HSPA8
ABHD17B	ALDOA	VEZT	CRHR2
CCT3	NR1D2	DOK3	PSEN1
FBXO28	PTAR1	VCAN	SMARCD2
DCP2	EXOC8	USP21	CDK13
FBN2	DOCK7	PCDHAC2	UBXN11
XPO6	ACAT1	WSB1	EIF2S1
RNF4	IVNS1ABP	TP53INP1	GPX8
TXLNA	CDC42SE1	CMKLR1	SUV420H1
CATSPERB	RP1-130H16.16	CCL7	GNG5P2
TMEM173	SPIN1	SLC4A2	CXXC5
MARS2	PDZD8	C12orf29	HIVEP1
HMGXB4	UTRN	MRPS2	SP1
FAM208B	MAP2K4	WDR11	DAB2IP
TOP2A	CNOT6L	GPRC5A	ARID5B
CCND1	EHD1	LIFR	SCARF1
IMPAD1	ZNF518A	ST3GAL6	LURAP1L
PBXIP1	CLSTN1	SUB1	CLDN1
ILF3	XRCC5	IGF2	MCRS1
TSSC1	RYBP	PTP4A2	MFSD5
LHFPL2	YAE1D1	CCT8	IDH2
WDR82	RND3	THOC5	DZIP1L
EPHB4	PAMCI(hsa)	CHERP	SLC39A6
ERGIC3	ALPP	RBM19	SYNJ2
FNDC3B	TXNL4A	TK1	FAM168B
JUNB	GTF2A1	PSMD11	C19orf10

MEAF6	EPHA4	FLRT2	DNTTIP1
CTNNB1	TRIM71	ARPC2	GTPBP2
CNOT7	UNC13B	SPRYD3	NPTN
ZNF678	PHIP	ORMDL2	ZNF451
POLDIP3	ST5	PRKCSH	MAX
PUM1	VEGFA	MAPRE3	CCDC61
ELOVL1	CPEB3	ZFP36L1	INO80E
SPEN	PTEN	SMAP1	AHSA1
MAPKAPK5	SYT14	GOLGA4	TNKS1BP1
THOC3	ZC3H4	CDCA7L	UBE2O
SNX27	DUSP16	ARID1A	ARRDC3
PTBP3	MARCHF6	PANK2	IKZF2
CBX1	DTX3L	FAM49B	ANAPC5
PPT1	HPS5	MTRF1	STX6
CRY2	PALLD	DEGS1	ILF2
NEBL	SPTBN1	CLDND1	SLC20A1
GPD2	PI4K2B	NMD3	NFE2L1
CTNNA1	ATL3	LLGL1	NEDD4
IRF2BPL	LATS1	DPH3	CNPPD1
PMM2	ACSL6	FBXO8	ATP5E
GFPT1	IRAK4	MTPN	MKI67
INPP4A	DAAM1	RRAGA	RRP1B
NR3C1	SOX2	CTSZ	UBE2V2
TEX22	CAP1	SOCS5	PRKAR1A
RFTN1	ALDH1A1	E2F6	PEF1
E2F5	RNF213	SKIL	VMA21
SHROOM4	TAF5	TFRC	CSNK1E
SSFA2	DYNC2H1	CABLES1	SGK494
ST3GAL2	PIGN	BLVRB	CYB561A3
MB21D2	MAPK1	PCNXL2	EP300
NRD1	LAMTOR1	TMEM38A	POM121
MMP1	IMP3	ADAM12	PPWD1
POLR2H	NIPBL	XKR7	AMOTL2
GIGYF2	SLAIN2	GPR45	SLC10A7
KCTD2	NUPL1	TMED10	GHITM
MYLIP	RNF168	KPNA1	ZNF35
RPL17-C18orf32	TLN1	MTMR14	MGEA5
TRAPPC9	NUP205	IFI16	MPRIP
CTTNBP2	RCC2	ANO2	ARFIP1
BHLHE40	RRAGC	SLC25A39	HARS
ARL6IP1	PPP2R1B	P2RX4	ZFYVE19
EPHA3	CHD9	SLC25A24	CASZ1
ASPM	SESTD1	FCHO2	GNAQ
CDV3	PRDX3	PDGFB	REV3L
DOCK1	CXCL10	COL1A1	EXOC2

NPEPPS	IL10	CHD3	ECI1
C2orf44	MMP-2(hsa)	KCTD3	AKT3
FAR1	AP3M1	XPR1	PRPF4
AQP1	BOC	IRF3	ZNF484
SAMD4A	KMT2C	CDC42EP4	CELSR3
NUFIP2	CYBRD1	PSMD14	PITPNB
GCC2	MGAT4A	CNNM4	CLN6
NEK4	CCNG2	SLC11A2	MYOZ3
WBP1L	GOLGA3	NDUFV3	ATF7IP
RAPGEF2	ZSCAN9	WASF2	BRD3
TSR1	GTSF1	GDE1	GRK6
SEC23B	TLDC1	ARHGDIA	YIPF5
BLOC1S6	NUCB1	ZBTB5	POMC
MSL1	PNPLA3	MYO5A	PHF12
AOX1	ZNF567	LRP1	TOPORS
TMEM243	MRPL49	SLC38A2	HNRNPH2
MRPL30	C20orf194	MYCBP	MZT1
LRRC58	COL5A2	COQ10B	DMPK
SLC27A1	ARF4	BAZ1A	CREB3L2
DYNC1LI2	ENAH	NCOR1	TSHZ1
CISH	SGCB	ZNF598	DHRS13
PAICS	RNF17	HIC1	CDH5
CCDC28B	KIF5C	TBRG4	RIN2
HIF1A	FAM79A(hsa)	DDB1	GBE1
PPP1R10	RHOB	EIF4G1	PARP1
HELZ	BRWD1	RAB11A	CTSB
POGZ	CBX5	RBBP5	H3F3B
PAIP2	TEK	NIPA2	PPFIBP1
RGMB	TBL1XR1	CEMIP	MPHOSPH9
SACS	SETX	CEP44	CAPN1
C2CD2	BAZ2B	SHISA5	GRAMD1A
CTNNBL1	PIGO	DENND6A	CLIP2
C8orf4	IREB2	LYL1	DDX39B
SLIT1	NA	VASH1	SOD2
ANP32B	TGFBR2	CERK	PAPD4
SEC14L1	COLGALT1	HIST1H2BG	FLT4
MAPK8	MEF2A	XYLT2	CEP170
KPNA5	GRSF1	KDM3B	AHCY
ICE2	DDHD2	JMJD1C	CHGA
PPAP2B	COPS6	NOL4L	ARCN1
STAT2	ZBTB47	HNRNPM	HNRNPH1
PDE8A	SNRNP70	RNMT	COL6A3
DNAJB9	RAPH1	SNX16	DOK4
RIT1	GCNT2	SMAD5	CCDC88C
HERC2	NBEA	BTN2A1	BSG

**CYP24A1**	**ITFG1**	**PDE12**	**RACGAP1**
**CSNK1A1**	**MDM2**	**FLNA**	**FASTKD5**
**CREBRF**	**ELOVL4**	**CYP2R1**	**CAPN15**
**CGGBP1**	**PSMB5**	**C5orf51**	**EIF3M**
**DNAJC13**	**PGK1**	**PSMC4**	**SYNGAP1**
**KIDINS220**	**SLC39A14**	**TGOLN2**	**TPST2**
**LNPEP**	**TOMM6**	**PTGS2**	**BAHD1**
**MED26**	**NIN**	**SLC39A9**	**GPSM2**
**AFF3**	**LPGAT1**	**RNF20**	**C5orf24**
**STAU1**	**PPAP2A**	**DAG1**	**ASS1**
**OGG1**	**FANCI**	**RNGTT**	**NTN4**
**FOXN3**	**DDR2**	**SF3A2**	**NCDN**
**RMND5B**	**TRIM38**	**WAF1(hsa)**	**CYP1B1**
**SRPK1**	**KIAA1715**	**UQCRH**	**SCD**
**HOXA5**	**VPS54**	**MRPL17**	**PDIK1L**
**LIMK2**	**PCMT1**	**MSANTD2**	**NR1D1**
**MRPL16**	**MEIS1**	**TBC1D25**	**LGALS1**
**HAX1**	**CNDP2**	**CFL2**	**CHAF1A**
**NR2C2**	**HAT1**	**HOOK1**	**PLEKHG2**
**KIF4A**	**BAZ1B**	**KLHDC3**	**NGRN**
**CENPF**	**CDC123**	**ID1**	**EDC3**
**SULT4A1**	**SMG1**	**GINS4**	**KIF18B**
**IKZF5**	**MCM3**	**APBA2**	**ZNF462**
**SON**	**BMPR2**	**FZD2**	**NBEAL1**
**HMCN1**	**KIAA1551**	**SEC24A**	**NUP43**
**PIM3**	**EIF4G2**	**TCF20**	**FAM102A**
**HCFC1**	**CLOCK**	**TEAD1**	**KMT2A**
**SF3B2**	**KBTBD7**	**RB1**	**RARG**
**CD46**	**KIF5B**	**WDR13**	**RPN2**
**AHCTF1**	**COG1**	**AEN**	**SCAF1**
**RGL2**	**39701(hsa)**	**BCAP29**	**EPS8**
**FBXO3**	**LMO4**	**NEK9**	**LGALSL**
**PRKAG1**	**CCDC6**	**SREK1**	**BAMBI**
**AHDC1**	**ZDHHC7**	**BAZ2A**	**TDP1**
**FAM83D**	**LIN7C**	**SMARCC1**	**LRRC1**
**MKLN1**	**PRRX1**	**SKI**	**HPS6**
**PLK2**	**PLS3**	**NADK**	**UBAP2L**
**GALNT1**	**USP34**	**CSNK2A1**	**ALDH9A1**
**TMEM91**	**GNE**	**FAM118A**	**MALT1**
**MYO9A**	**CD44**	**MARCKSL1**	**DNHD1**
**STRN4**	**KIAA0020**	**TMEM185B**	**TPP1**
**EDNRB**	**CD47**	**PRPF31**	**FOXO3**
**AGAP2**	**KIAA1430**	**ARSK**	**HDAC6**
**SFXN5**	**COL4A1**	**ARL4D**	**ZNF585A**
**VCAN**	**ZKSCAN1**	**PRPF38B**	**DDR1**

USP21	SNAP29	USO1	MAPK8IP3
MTCH1	CDC25A	CNKSR3	LMNA
USP46	BNIP2	UBR2	SLC7A2
WSB1	FNIP2	DOCK4	TULP4
ACO1	C2orf43	IDH1	TSC22D4
TP53INP1	RXRA	POM121C	FAM136A
INSIG2	ATP5O	HSP90B1	STARD7
GPAM	ZBTB8A	FARP2	ATF3
MLH3	EIF3I	H2AFY2	IPO8
ITFG3	DSP	PHC2	MYO1E
FXYD6	EDIL3	YWHAZ	BASP1
LIFR	GSPT1	C11orf84	SGTA
LBH	ZDHHC13	YLPM1	CTCF
POLR1B	RMND5A	CUEDC2	GFRA1
BCL7B	C7orf55-LUC7L2	EIF4ENIF1	PXDN
SUB1	ZCCHC3	GLCCI1	FAM173A
DIAPH1	TXNIP	HDAC2	RAD54L2
PTP4A2	CCR6	FBXL19	AKTIP
TMEM9B	NCOA4	NRP1	COBL
CHERP	TADA2A	KIAA0284	APOOL
SPRYD3	RP1-130H16.18	PRKAR2A	MATR3
PRKCSH	GTF2I	ZNF256	LRIF1
CLIP4	SLC6A14	NAA30	TNC
RELN	CTDSP2	NTPCR	AKAP12
ZFP36L1	CPNE1	MBD6	GSDMD
COL5A1	ZNF28	GDI1	C21orf59
PLEKHA3		PHLPP1	TMEM30A
GOLGA4		PSMB2	PPAN-P2RY11
GPBP1L1		WDR81	BAG6
BLOC1S5-TXNDC5		SEMA4C	CDKN1A
KLF3		ZC3HAV1	STMN1
ATXN1		DHTKD1	UHMK1
UBA1		PPP2R3C	PRMT2
EPS15		CHAC1	BDP1
ESM1		RAP2C	KIAA0232
PRDX1		KAT2A	SYNE2
STK35		TMPO	OSBPL1A
PPP2R1A		SRRM2	NANS
FAM126A		RNFT1	EDARADD
IGF2R		TEX261	GPT2
DYNLT1		RPAP1	TNRC6A
KANK1		EOGT	TP53INP2
UBAP1		PEX11B	MBNL1
SLC16A1		SENP1	WDR43
CLPX		TBK1	ZFHX4

GALNT3	ICOSLG	ANKHD1-EIF4EBP3
SOCS5	EPHA2	CNEP1R1
E2F6	ASCC3	MCAM
SKIL	SYT7	KIF21A
TFRC	CUX1	ATG9A
BIRC6	SEC31A	ZBTB18
IKBKAP	XRN1	TDRP
SLC25A23	ZNF74	EIF4EBP2
BLVRB	RAC1	TUBA4A
FLNB	INSR	SMG7
DSN1	SNX2	D2HGDH
DAPK1	SRC	FAT1
BMP2K	TCOF1	HNRNPA2B1
NEK6	MAPRE1	FOCAD
JRKL	ZSWIM4	POLG
SPAG9	STC2	NOP9
SUPT6H	PRKAA1	MYL6
MAPKAPK3	PRMT1	HK1
TMED10	CAP2	ZBTB1
ATAD2B	ANKFY1	CHST2
REL	DDOST	ADARB1
IFI16	CTPS2	TTI1
SRPR	FAM208A	PPM1B
FRMD6	PTP4A1	TNPO2
HAUS6	PEBP1	ZBTB4
DMTF1	NOLC1	NOL8
WT1	SHOC2	IFI27L1
GLO1	PMPCB	GORASP2
VCP	KMT2D	PTPN12
CBX6	HOXC5	LMF2
CHD3	ADAMTS1	CCNI
ZMYM1	FHDC1	TMEM263
ECT2	FSCN1	ERalpha(hsa)
XPR1	LDLRAD3	HNRNPL
MAFK	KDM6A	RUFY2
ANKLE2	UBE2K	PHF13
ZBED6CL	THAP2	CCNJL
ZMYM2	KDM6B	HSD17B4
SMURF1	IPO4	RASSF8
GPR56	SESN1	STAT1
APLP2	DOLK	HIPK1
GIPC1	FAM122A	AHNAK
NDUFV3	ANKRD46	LUZP1
TM2D3	TSEN34	YTHDF3
GMPS	SESN2	PRKDC

PRNP	NARF	SASH1
HSP90AB1	RAP1GDS1	ZBTB34
ZFYVE9	TRIO	AP1G1
NFKBIA	BMP2	SMAD7
MYO5A	KCNC4	PAFAH1B2
BAZ1A	TMED4	KIF1C
HIC1	CS	PRKAR2B
ZNF100	TNFRSF10B	EHD2
SNAPC4	NLGN2	NDUFV1
RBBP5	KDELR1	SETD6
NIPA2	TIA1	RHBDD1
CEMIP	SURF4	SPHK1
PPP4R1	DZIP1	PPIF
SHISA5	C16orf72	TRRAP
RAB18	MRGBP	ZFP36L2
TMEM87B	TSC22D2	C11orf31
C17orf58	PYGO2	PIEZO1
DENND6A	SLC12A9	PPM1G
HIST1H2BG	IGF2BP3	ZNF3
XYLT2	ELAVL1	HIST1H1B
KDM3B	COPG1	ZNF106
JMJD1C	CCER2	PSMD8
COPZ1	PHKA1	FAM134A
RNMT	TMEM2	ZC3HAV1L
GLUL	LYPLA2	KHNYN
RBM26	SSX2IP	MAFG
GBAS	EMC4	AGPAT3
GALK2	AP2A1	ULK1
KIAA2013	CCDC50	GATAD1
TLE4	CALD1	PRKD2
MID2	ADNP	UQCRC1
NCAPD3	HSPA8	CTDSPL2
CLK1	MAD2L1	HTR2C
ZNF138	LCLAT1	CMTR1
H2AFZ	C6orf120	NR1D2
C5orf51	FIGN	RPS4Y1
TRIM33	CCNY	FGFR2
ELOVL5	PNPO	METTL3
MMD	CDK13	UBXN2B
CDC42BPA	STK25	MOB1A
ARIH2	RNF152	HEY1
PATZ1	MED28	KIRREL
DAG1	UBAP2	CHST1
RMDN3	RANBP2	ITGA6
CACNG4	DSG2	SLC30A3

RNGTT
RP11-385D13.1
NCOA2
POP5
SAP130
PABPC1
CDYL
EI24
MEPCE
RBM47
THAP11
CDCA7
HOOK1
KLHDC3
SEC62
NOL11
SEC24A
PIK3R1
NEO1
PCMTD2
ARMCX3
TEAD1
PNRC2
SCYL2
GEM
ERP29
BAZ2A
SATB1
SMARCC1
TGIF1
ASH2L
FKBP8
MARCKSL1
TMEM185B
WDTC1
KDM5A
SEPP1
CPEB2
TMCC1
RORA
SLC22A23
CDK18
MRPL43
PTPN11
BUD31
SUV420H1
ULK3
YY1
CXXC5
SP1
ARID5B
B4GALT3
DUSP7
VSNL1
KLHDC8B
COL1A2
CCDC15
FAM98A
DUSP3
RBPJ
MFSD5
MDM4
IDH2
ACVR1C
ADRM1
SYNJ2
FUT11
SMIM13
PLXND1
C19orf10
EGLN2
ONECUT2
MGAT1
HIST1H2BL
RBPMS
ANGPTL2
AKIRIN1
CDC23
ZNF451
GNPTAB
MAX
NRARP
TSPYL4
MYH15
ARRDC3
USP42
IKZF2
FAS
PGRMC1
ANAPC5
PRKRA
PHLDB1
ATP6V0D1
UTRN
PBX2
CNOT6L
EHD1
RUSC2
FLOT2
SHH
RPS6KA4
CLSTN1
DKK3
SSBP3
PHEX
CHKA
RELA
ITPR3
TMCO3
CHMP1A
TANC2
PSIP1
TMEM170A
FAM171A1
PTEN
ZNF652
APBB2
MTF2
TUBB4B
EWSR1
DUSP16
MARCHF6
HPS5
STX4
SIX4
SPTBN1
HNRNPUL1
CCDC85C
ATL3
SLC35D3
FAM160A2
ACADM
BRAT1
AP1B1
KIAA1841

HSP90B1	MBNL2	C9orf91
MAD2L2	NMT1	NFIC
ARPC1A	DOT1L	SORD
CDC42SE2	PRKAB2	RNF213
C2orf49	MELK	INF2
FARP2	PEX13	DCAF16
TMEM39B	LAMC1	PPFIA1
PHC2	USP24	RNF141
DYNC2LI1	RAB40C	MAPK1
GTF2H2	RPS6KA3	PNRC1
ERAL1	KCNJ2	HSPA12B
NRP1	NFE2L1	AMER1
CRELD1	C14orf166	SLAIN2
MPP6	ERC1	TLN1
PRKAR2A	ZNF414	NUP205
PRKX	MKI67	RCC2
ZNF592	TSPAN14	SPTSSB
TUT1	RRP1B	AMOTL1
BHLHE22	TGFBR3	MICAL3
ZC3HAV1	ASCC2	SRF
TAF4B	PCYT2	MAPK6
SRRM2	FAM160B1	RRAGC
SS18L1	MANEAL	ADSL
DLGAP5	TTLL11	CREBBP
AGPAT6	PLIN2	C22orf29
KLHL15	SLC4A5	CHD9
FXR1	CD200R1	GREB1
SLC30A9	EP300	ABL2
PPIL4	POM121	KDR
PEX11B	MIDN	TRAF3
SENP1	PRDM2	WRNIP1
JAZF1	HDLBP	RAD21
GNPNAT1	SLC10A7	SERPINE1
GALNT10	RNF165	NKIRAS2
METTL9	CBLL1	ARHGEF4
EPHA2	BTG3	SCARB2
MID1IP1	PAAF1	AP3M1
ASCC3	ARL5B	CDK16
EIF5B	ZCCHC14	HEATR2
CERS5	PIGT	WDR77
LARP4	GPCPD1	PUS1
BACE2	SAMD10	ADCY9
PTPRJ	CTSA	GOLGA3
DIS3L	TP53BP1	BTG1
KITLG	BCCIP	HOXA10

**TNFRSF21**	**TTLL4**	**NRN1**
**KBTBD11**	**RPUSD3**	**USP22**
**SYNE1**	**PLAG1**	**SERTAD2**
**SRPX2**	**S100PBP**	**GPR151**
**IRS1**	**PUM2**	**VAT1**
**WDFY3**	**ATXN7L3B**	**SEPHS2**
**LZTS2**	**PIAS4**	**CAPRIN2**
**GLCE**	**REV3L**	**UBE4B**
**RAC1**	**RSBN1L**	**NUCB1**
**NRP2**	**ZNF511**	**TMEM136**
**SNX2**	**OSBPL10**	**PCM1**
**GABPA**	**TTC22**	**ATP5G2**
**CD99L2**	**CELSR3**	**SERBP1**
**FAM120C**	**CLN6**	**TMUB1**
**TNFRSF25**	**RHBDF2**	**ENTPD4**
**SPRY4**	**FBXW2**	**SETD1A**
**CHRAC1**	**BRD3**	**KLF6**
**ANKRD13C**	**ARHGEF15**	**SLMO2**
**RANGAP1**	**TGM2**	**MYL12A**
**DUSP8**	**MYLK3**	**MRPL52**
**STC2**	**GRK6**	**PSMD6**
**MAPK3**	**GJC1**	**MAPKAPK2**
**PRKAA1**	**YIPF5**	**TMEM165**
**CAP2**	**CDK12**	**RBM4**
**UBXN2A**	**CNOT1**	**RHOB**
**CTNNAL1**	**ZNF281**	**NCKIPSD**
**ANKFY1**	**UBE2D3**	**CBX5**
**FAM208A**	**USP14**	**TNKS**
**SDAD1**	**CASP6**	**TEK**
**NOLC1**	**STARD13**	**NOP58**
**MESDC2**	**EIF4E2**	**JMY**
**ATP6V1A**	**COX10**	**HIST1H3J**
**CDC42**	**MPL**	**UQCR11**
**KMT2D**	**CREB3L2**	**HYOU1**
**SEPN1**	**DIABLO**	**GOLPH3**
**ADAMTS1**	**TSPYL1**	**TIE1**
**DNAJB14**	**CDH5**	**SLC1A5**
**FSCN1**	**CKAP4**	**EPHX2**
**PAK6**	**SHE**	**EIF1**
**KDM6A**	**POLQ**	**SLC39A1**
**UBE2K**	**GBE1**	**GLYR1**
**NUP50**	**ATP8B3**	**TONSL**
**KDM6B**	**PARP1**	**MAML1**
**BCORL1**	**CTSB**	**MGAT4B**
**PEA15**	**UBE2G2**	**RPL15**

IPO5
IPO4
LDHB
SESN1
AIF1L
TSEN34
SESN2
VPS52
SDE2
HSPA4L
YRDC
POLR3C
KIF11
CS
FRYL
C2CD4A
COMMD2
DSC3
TIA1
PIK3C2A
CIPC
C16orf72
KCNJ6
MRGBP
RPL19
TSC22D2
TXLNG
KIAA0247
TMEM2
LYPLA2
ADCK3
DNAJC12
MIOS
SNRNP40
ZBTB21
DHX15
BAG2
DYNLL2
AP2A1
CALD1
ADNP
RPS27L
C6orf120
NF1
ZNF430
H3F3B
FOXP2
ZFYVE20
CLPTM1
GRAMD1A
RIIAD1
STAMBP
RGS16
RNF167
TRMT112
SOD2
MBD4
PAPD4
IDE
CYTH1
PCDH7
ZFR
VPS33A
TRAPPC10
PYGB
ACTL6A
SMIM3
NMNAT1
CDC27
KPNA6
SLC1A4
KAT5
BAHD1
TLCD1
GALNT2
KIAA0355
GPSM2
PHLDA3
PLXNC1
ABI1
SPINT1
RPL22L1
SLC6A9
ATP6V1C1
CYP1B1
SCD
SAMD4B
VPS53
LRGUK
TFIP11
SNRNP200
PTGES3
SGK1
PRRC2A
ATP5SL
RAPH1
SYNPO2
JAK1
RAB5B
DEF8
MAF1
CD63
AKAP11
MAP7D1
ELOVL4
ICT1
RNF126
DYNLRB1
SLC39A14
CMPK1
MTDH
TOMM6
LPGAT1
PRR11
FANCI
ABL1
ICA1L
RABL6
PCMT1
VKORC1L1
GNB5
BAZ1B
SMG1
BMPR2
RPS29
RTN3
TMBIM6
PGAM5
GINM1
EIF4G2
ECE1
KIF5B
MTF1
NCOA1
NOA1

STRN	C14orf28	FBXW9
HMG20A	DNA2	HLA-A
CDK13	SUV420H2	LIN7C
KIAA0100	NGRN	SIN3A
UBAP2	AURKA	PLS3
NIFK	ZNF644	MLLT10
CACYBP	ZNF512B	ANKMY2
RANBP2	THOC2	CDC42BPB
SUV420H1	GPN2	ZNF689
DTNA	NOC3L	SIDT1
ZFP1	NUP43	DDX49
SPSB1	PLEKHA1	C21orf33
HIVEP1	SGOL1	SARS
SP1	KMT2A	PRKACB
ARID5B	ROGDI	XIAP
B4GALT3	RARG	EGLN1
DUSP7	RPN2	MAP1B
ABCB7	SCAF1	APH1B
COL1A2	AKR1A1	CTGF
JAG1	VEZF1	MCF2L
FAM98A	MYO5C	DCHS1
MDM4	TAF5L	QRICH1
OGFRL1	MED19	GSTCD
CSNK1G1	PXT1	COL4A1
ACVR1C	GRPEL1	PFKFB3
KRT80	TMEM8A	UBR4
SLC39A6	HOXD10	ZKSCAN1
FUT11	CHD5	SENP5
SMIM13	MEX3D	NACC2
PLXND1	TPP1	TTC9
C19orf10	LASP1	IDH3G
ONECUT2	RLF	ANAPC16
KLC1	FUT10	TRIM28
TWSG1	IGSF3	WHAMM
AGAP5	ZNF585A	ASAP2
AKIRIN1	FBXO22	ATP5O
NPTN	LMNA	TOR3A
TNRC18	NDC1	DSP
ZNF451	DRG2	RPL10
GNPTAB	CANT1	RIN3
MTERFD3	FN1	KDM5B
TLE3	TSC22D4	GSPT1
AP3D1	ACSL1	CYB561D2
CNOT2	NWD1	NDEL1
RASSF3	MYCN	ADCY6

TAF7
USP42
NFE2L2
EPC2
ANAPC5
GFM1
MBNL2
STX6
DOT1L
SLC20A1
PPP6R1
NDUFA8
LAMC1
PRICKLE2
USP24
RAI14
RPS6KA3
NFE2L1
NEDD4
FOXJ3
ERC1
CDK19
ATP5E
MKI67
SCOC
IGF1
ELP6
TGFBR3
CNTLN
UBE2F
LAMP2
PRKAR1A
SUMO3
FAM160B1
UBL4A
KIF26A
MIDN
AMOTL2
BRE
PRDM2
ZNF714
DDAH1
SLC10A7
FCGRT
PTPRA
NECAB3
UBN2
MYO1E
SLC35E3
ATP7B
OR2AG1
PXDN
RAD54L2
PKN2
CALR
AP3S2
TEAD4
CDKN1A
PYGO1
PCIF1
UHMK1
LHFP
DCAF15
BIRC3
RHOF
ATG12
CCNJ
IRAK2
C3orf38
NANS
CHTF8
HIAT1
TNRC6A
TRAPPC1
SLC2A1
MAP3K2
RBM10
ARF6
RSPRY1
USP9X
SAMD5
TMEM201
WDR43
NFIA
GFM2
MON2
ZFHX4
SRSF11
PAN3
INPP5A
RMND5A
ZCCHC3
RBM15B
HIST1H3B
HIST1H3F
CYFIP1
OTUD7B
RP5-850E9.3
HIST1H3I
HIST1H3E
HIST1H4L
SNX12
GPR75-ASB3
HIST1H3G
SYNRG
LIX1L
ZFP91
CTDSP2
DDX52
HIST1H4A
BRD4

AHSA2	ANKHD1-EIF4EBP3
BTG3	AVL9
PPARG	UBR3
MYT1L	PLD3
POU2F2	TNFAIP1
PAAF1	RFC5
MGEA5	ATG9A
DENND2D	POLL
ARL5B	MON1B
CASP8	CARM1
NOTCH3	SCYL1
TTLL4	EIF4EBP2
PLAG1	MLXIP
PUM2	SUV39H2
NFIB	RRM2
THUMPD1	VGLL4
C2orf47	CAD
TWISTNB	COPA
RASEF	KLHL12
C12orf57	FAT1
CHML	C17orf51
OSBPL10	EIF1AD
STON2	DONSON
ATF7IP	ZNF555
IGFBP2	GTPBP4
MEDAG	ZNF473
PLEKHB1	NUDT16
COL11A1	PFKFB2
GJC1	PRDX6
LY6G5B	PPM1H
POMK	NUP155
CNOT1	TPT1
ZNF281	WDR24
EFNB1	PARD3
ATG3	TTI1
CEP135	GORASP2
UBE2D3	HMGCS1
ZZEF1	BCOR
STARD13	GAREM
PHF12	HOXC6
TSHZ1	NCKAP5L
PPARGC1A	MMRN1
TSPYL1	BTBD7
SCAF11	HNRNPL
CBY1	SLC25A27

CYB561
CDH5
CKAP4
PARP1
UBE2G2
H3F3B
GPR160
SEC24B
MPHOSPH9
CAPN1
SLC4A8
CLIP2
CNN3
TMEM150C
CD99
GAPVD1
G3BP2
RBBP7
B4GALNT1
TP53I11
ZFR
TTC39A
SUZ12
TRAPPC10
HNRNPH1
OAZ2
ABCA5
CCDC88C
FAM210A
RC3H1
SPX
CDC27
KPNA6
IGFBP3
FASTKD5
CAPN15
MYCT1
ZNF81
GALNT2
LONRF2
PJA2
ZBTB33
FAM193B
ABI1
VPS39
ERGIC1
PPIG
ATP2A2
CCNJL
AFAP1L1
MPZL3
RASSF8
EDN1
AHNAK
YTHDF3
PRKDC
CNBP
ZBTB34
SH2B1
SMAD7
PAFAH1B2
KIF1C
SRGN
E2F4
ENTHD2
BVES
TNFSF9
RPL9
GLRX5
UBE2W
C1GALT1
RBM4B
PDE7A
SPHK1
DUSP5
ZBTB32
PPIF
TRRAP
YIPF1
VPS25
ZFP36L2
ARHGEF7
PIEZO1
DLAT
AURKB
USP11
PSMA4
GPR157
ABCC5
ANAPC13

DDA1	PRKD2
ABAT	ZCCHC10
NCDN	CTDP1
ATP6V1C1	ARHGEF17
NGLY1	RNF6
CYP1B1	FOSL1
LAPTM4B	SNX25
KLHL9	WIPI2
MARCHF5	KLHL24
UBE2G1	ALDOA
SNRPB	CTDSPL2
TFIP11	PTHLH
RCBTB1	LEPROT
SREK1IP1	CLDN12
RLIM	UHRF1BP1
IL6ST	SRSF10
SEMA7A	KREMEN1
ZNF462	DPH2
THOC2	ZNF449
NAPB	MOB1A
ENPP1	DOCK7
FAM102A	PRDM4
LEF1	VCPIP1
OCRL	CDC42SE1
MRPL28	KIRREL
KMT2A	GFOD2
RPN2	IGDCC4
EPS8	SLC30A3
VEZF1	TADA1
UPF2	JAGN1
BAMBI	PDZD8
CLK2	LUC7L
PRMT5	PFKL
PACSIN2	SUCO
ZER1	UTRN
ALDH9A1	MAP2K4
ZNF682	PBX2
GPI	C9orf64
TPP1	EHD1
LIMK1	KMT2E
LASP1	PTPRB
RLF	USP3
MECOM	CHKA
FOXO3	VAV3
DHX9	ITPR3

ANKRD28
DERL1
IFNG
FBXO22
LMNA
SLC7A2
CANT1
TULP4
FN1
FAM136A
STARD7
IFFO1
SEPT2
IPO8
RNASET2
ERI3
ANXA11
CTCF
GFRA1
RRP12
PXDN
RAB22A
TMEM218
TXN2
RAD54L2
SEMA4D
LRIF1
TNC
ROBO1
AKAP12
CALR
ELOF1
EIF4G3
TMEM30A
RBM12B
CDKN1A
ERH
PCIF1
STMN1
UHMK1
RALB
POFUT1
NAPEPLD
SYNE2
CPT1B
DNAJA2
CEP290
SPHK2
RFWD3
XPO1
EPHA4
TRIM71
AP2S1
NFATC3
MTF2
FGFR1
FUCA1
HNRNPUL1
AGL
LIN54
TRIM25
PHF21A
LATS1
SLC35F6
FOXO1
AP1B1
SALL4
ZSWIM8
SOX2
BLOC1S4
RNF213
EMC1
CTPS1
BBX
INF2
CAB39
TAF5
PCYOX1
ATP13A3
TRIB1
MAPK1
MFI2
MVK
PNRC1
TTLL9
MAP3K7
FNDC3A
NUPL1
RAD54L
DDX26B

C3orf38
GTPBP10
CDC42EP3
GPT2
DOCK9
TNRC6A
MDN1
ZNF662
AKR7A2
USP9X
IRX3
MBNL1
ISL1
WDR4
TMEM201
EIF6
TBP
MYBBP1A
COBLL1
ATP2B4
LTBP1
NOL10
PAN3
ANKHD1-EIF4EBP3
MKNK1
ZMAT2
MCAM
SEMA6D
KIF21A
PRKD3
ABCA12
ZNF579
CHAMP1
HSD17B12
KIAA0586
ZBTB18
REEP1
PRKCH
PPA1
HHLA3
MON1B
CARM1
TUBA4A
MLXIP
KIFC3
KANK3
LRRC41
TLN1
HIST1H2AC
ITPR2
SRF
MAPK6
MMP14
MED6
CREBBP
C22orf29
C9orf37
FBXW11
GATAD2A
RHOBTB2
CHD9
SESTD1
ZNF146
RNASEH2B
MAFB
ABL2
TMPPE
GNG5
TET2
SLC43A3
PCGF3
ZNF33A
ESRP1
AP3M1
DNAJC27
CDK16
APLN
TBPL1
PIP4K2C
MGAT4A
PUS1
ADCY9
PSMC6
HELLS
GOLGA3
KATNAL1
SERTAD2
ZSCAN9
RNF26
DLGAP4

PDZD11
RRM2
SMG7
VGLL4
CKAP2
SESN3
LDLR
FAT1
TSEN54
C17orf51
HNRNPA2B1
TEX15
FOCAD
HRSP12
PDPK1
NOP9
PPM1H
NUP155
TPT1
TRIM44
DKK1
LRP3
ZBTB1
POLA1
FIP1L1
HMGCS1
FAM47C
BNIP3L
BCOR
LIMA1
PNMA1
TUB
CCNI
HOXC6
BTBD7
OPA1
ERalpha(hsa)
HNRNPL
PHF13
PPIG
MAP1A
ATP2A2
NAV3
RPL23A
AFAP1L1
CAPRIN2
VRK1
TLDC1
UBE4B
PCM1
PATL1
ZNF567
TRIM32
MTCL1
ALG3
SETD1A
FNBP4
C20orf194
GLB1
ARF4
ZNRF3
ZNF623
MYBL2
OS9
TNPO3
ZNF17
KIF5C
CDC42EP2
PLBD2
GPR161
STK40
SLC12A7
RHOB
SIM2
BRWD1
CBX5
STARD4
TEK
JUP
JMY
PIAS1
DMKN
9-Sep
WDR37
DNASE1
LEFTY2
SNRPC
BAZ2B
DUSP1
GATA6

CCNK
ID3
RASSF8
HIPK1
AHNAK
TINAGL1
PRKDC
RINT1
SASH1
ZBTB34
SH2B1
AP1G1
SMAD7
COL14A1
KIF1C
EYA1
E2F4
KCNB2
TNFSF9
MEF2C
GLRX5
PLEKHH1
UBE2W
ZNF726
ABCA1
TMEM127
RAN
NDUFV1
MKNK2
PSRC1
FANCA
ZIC5
DCLRE1B
STAT6
STAB1
PDE7A
PALM2
PPIF
ADD1
GDI2
JADE1
MTOR
TRRAP
ARPC4
PRR13
FOXF2
ARHGAP28
BZW2
TIE1
SECISBP2L
TGFBR2
GPRIN3
EIF1
C3orf52
SLC52A3
BICC1
MAML1
PON2
USP10
TMEM60
SNRNP200
COPS6
PTGES3
PRRC2A
MBOAT2
FADS3
GCNT2
JAK1
EXO1
CABIN1
ZNF142
MDM2
SH3TC1
WNT9A
MTDH
GPR137C
LPGAT1
ZNF629
ABL1
NOP16
PLCB4
AP5M1
KIAA1715
NIPA1
VPS54
BMP4
SNX24
EHMT1
COIL
SVIL

VPS25
ZFP36L2
PPM1G
PLEKHA6
PHF5A
ZNF106
NCKAP1
MAGT1
GATAD1
DYRK1A
CTDP1
CMTM6
ABHD17C
FOSL1
MOAP1
WIPI2
C1orf115
ALDOA
BCAR3
NR1D2
SLC35B4
PTAR1
DENND2C
EXOC8
SLC30A2
METTL3
MOB1A
ZNF574
NFASC
VCPIP1
ITGA6
ZFY
SGPP1
PDZD8
ATP1A1
PFKL
SUCO
ST20
UBE2B
UTRN
MAP2K4
XPC
EHD1
RUSC2
AKAP6
BAZ1B
SMG1
MCM3
STK24
BMPR2
RPS29
TMBIM6
ATF4
H1FX
NNT
ZNF141
BCL2L11
RHOG
ECE1
MED8
SYT16
TBRG1
NR2F2
ZMYND8
HOXC8
SLC27A4
SOBP
DAXX
LIN7C
ABCF1
SIN3A
MLLT10
ANKMY2
ZNF689
WBP11
ARRB1
PAX3
ZNF695
MAP1B
FLCN
EPB41
SOX5
EIF3C
MICAL2
DHX33
COL4A1
PFKFB3
SENP5
CDC25A
FNIP2

SHH
ZNF518A
RYBP
RND3
PTRH2
TMCO3
COL5A3
NME1
ZNF618
GTF2A1
TANC2
XPO1
PHIP
FAM171A1
FBXO33
SCNN1A
ZNF408
CPEB3
PCF11
ZC3H4
MTF2
TUBB4B
DUSP16
MARCHF6
DCTN6
HPS5
PALLD
SIX4
FUCA1
SPTBN1
CHD6
ATL3
LIN54
TRIM25
CD55
FOXO1
AP1B1
IRAK1
CAP1
RNF213
ADAMTS9
BBX
CAB39
PCYOX1
ATP13A3
TRIM28
PEX1
SYNCRIP
BIRC2
RIMKLA
KCNH2
ATP5O
DSP
INO80D
IL6R
LRCH4
UBOX5
C9orf142
GSPT1
COA7
SMCR8
UTP11L
DSEL
EFNA1
TUSC2
C7orf55-LUC7L2
ZCCHC3
LUC7L2
KMT2B
PIGW
GGNBP2
SOCS7
MT-CO1
MT-ATP8
MT-ND5
OTUD7B
BOP1
LHX1
RP11-201K10.3
NR4A1
ZNF25
MARCKS
SERPINA3
GPR75-ASB3
ARHGAP23
ZNF792
H2BFS
VAMP3
RPS6KA2
TMEM189-UBE2V1

PSMF1
SPATS2
RNF141
DYNC2H1
TRIB1
NSFL1C
QPCTL
MAPK1
MAP3K7
FNDC3A
BRI3
SDCBP
SLAIN2
PAX9
NUPL1
MTMR3
RNF168
RCC2
METTL7A
AMOTL1
MAPK6
MED6
PTGFRN
CREBBP
FBXW11
GATAD2A
TMEM231
PPP2R1B
SESTD1
C9orf114
PRDX3
ABL2
CSF1
ZHX1
IL10
RAD21
HECTD4
HMMR
AP3M1
CIRBP
DNAJC27
KMT2C
HEATR2
APLN
TBPL1
ZFP92
HIST1H4D
SARS2
ZNF280B
PSMB3
MUC16
BRD4

GRB2
MGAT4A
FAM84B
CALCRL
BTG1
HOXA10
ITSN1
KIAA0895
USP22
EIF2B4
RNF26
C17orf70
DLL4
SLFN5
SMAD1
UBE4B
VBP1
RAB10
PCM1
PARD6B
SERBP1
TMUB1
CPNE8
TRIM32
DNMBP
MTCL1
C1GALT1C1
LYSMD3
TAF3
KLF6
SRP14
NGDN
KIAA1279
MYBL2
ENAH
ARHGEF26
ITPK1
RAB20
ZBTB25
GPR107
DNAJC2
MAPKAPK2
GPR176
ZNF675
RNF139

KNTC1
SLC12A7
KPNB1
RHOB
C19orf24
BRWD1
CBX5
TTPAL
CDK10
JMY
MXRA7
TBL1XR1
SEPT9
WDR37
SETX
GALNT5
C14orf132
LETM1
GATA6
RNASE1
ATRN
IREB2
SPIDR
CCP110
GPS1
SECISBP2L
TGFBR2
SLC1A5
MEF2A
EIF1
C3orf52
BICC1
RAB14
ORC5
PON2
MTSS1L
TMEM60
ATP2C2
PLA2G15
SGK1
PRRC2A
ZBTB47
ARHGAP5
ATP5SL
RAPH1

KHDRBS1
PLEKHG1
JAK1
RPA2
RAB5B
CABIN1
MAF1
CLDN3
AKAP11
GLYCTK
SLC46A3
MDM2
TTC5
SLC18B1
PGK1
ARMCX2
WNT9A
MTDH
TOMM6
SYTL5
NIN
ZNF669
TNRC6C
SLC35E2B
N4BP1
SCML1
ABL1
KIAA1429
EHMT1
SVIL
STK24
BMPR2
KIAA1551
PNISR
TMBIM6
ARHGAP12
TOR1AIP1
ZW10
EIF4G2
BCL2L11
ECE1
NR2F2
ZMYND8
MAP3K5
ZMYM3

KIF5B
MTF1
ILK
ZFP91-CNTF
LIN7C
SP6
SIN3A
CDC42BPB
PDGFRB
HOXA13
RPS6KB1
ARRB1
ERLEC1
CD44
ARHGEF28
KCTD20
EGLN1
MAP1B
FLCN
EPB41
MIER3
ADH5
DCHS1
DHX33
SIAH2
KIAA1430
DGKH
TRIM26
SLC30A1
UBR4
ZKSCAN1
SENP5
NACC2
CDC25A
C2orf69
BNIP2
FNIP2
VDAC3
TRIM28
YY1AP1
RXRA
RIMKLA
SPRY2
MEX3C
TRAPPC8

EIF3I
LRFN5
INO80D
PANK1
SEC11A
p53(hsa)
KDELR2
NEURL4
B4GALT7
KDM5B
GSPT1
NDEL1
COA7
ADCY6
SMCR8
UTP11L
TUSC2
ELF1
RMND5A
OR7C2
ZCCHC3
TXNIP
KMT2B
HIST1H2AH
HIST1H4E
PIGW
GGNBP2
PILRB
PIP4K2B
GTF2H5
ZNF8
NUDT3
C5orf22
HIST1H2BJ
SNX12
MARCKS
GPR75-ASB3
MYO19
VAMP3
RPS6KA2
ACACA
ZFP91
CTDSP2
CPNE1
PECAM1

BRD4
ZNF28
F11R

miR-181a-5p	miR25-3p	miR31-5p	miR28-3p
BMI1	CALU	TBC1D20	BMI1
FAM47B	ZKSCAN5	CAMTA1	VAPB
CCNT2	POLR2B	ATF6	ESR1
POLR2B	RTN4	TMEM120A	BRK1
RTN4	ABCD4	ANP32E	SLC25A44
SLK	RPRD2	ACTB	CECR1
WWC2	ZNF503	CCNG1	HOXB7
E2F7	PELI1	PCNXL4	ACTB
PELI1	DDI2	JARID2	EAF1
DDI2	CAMTA1	DFNA5	SLC38A1
RAB2B	RPA1	CRTC3	SNAP23
TWF1	IRS2	CCNB2	IQCJ-SCHIP1
ZNF419	RBM14	IL1RAP	PPP1R2
DTD2	RNF11	URGCP	FOS
IRS2	BRAF	FARP1	ZEB2
FHL2	FHL2	ITPRIP	TCF3
SFT2D1	ANP32E	SCAMP2	MTA3
ANP32E	TNFRSF11B	SPARC	BICD2
ZNF544	ACTB	GUCD1	ADIPOR1
ACTB	CCNG1	STAT3	RNF44
CCNG1	SLC38A1	TAGLN	CREBL2
EAF1	PHLPP2	CCAR2	WAPAL
EIF3L	MTO1	C2CD3	PKIA
SLC38A1	TMEM50A	RAB3GAP2	MFAP3
RP6-24A23.6	BRI3BP	ZSCAN25	MYBL1
DUSP4	NRCAM	TBC1D13	MGAT2
PRLR	NEK7	NPDC1	SPARC
ZNF239	PAQR3	YIPF3	UGCG
NEK7	SAP18	NFKB1	SAR1A
MSI2	SERTAD3	FAM189B	B3GNT5
SEC16A	PPP1R37	OAZ1	RREB1
DCP1A	MLK4	MET	FERMT2
RAB3GAP1	ZMAT3	ATP6V0E1	CDH2
FOS	JARID2	GRB10	G3BP1
MLK4	EDRF1	CLEC14A	ZNF638
JARID2	BICD2	DCTD	DIRAS1
NETO2	GSK3B	IL1R1	MORF4L1
HIPK2	RNF44	RNF38	HMG2L1(hsa)
TMED8	ALKBH3	HTRA1	GGCT
GSKIP	ASB7	WDR47	CHST3
C5orf30	FAM120A	THEM6	RFESD
SOX4	C5orf30	CDK4	FAM53B
ARL4A	SOX4	CCDC92	LRP6

**FBXL3**	**MCOLN2**	**EIF2AK3**	**IPO11**
**FBXO11**	**UIMC1**	**BRD2**	**SMC1A**
**PKIA**	**RBL2**	**ASCC1**	**MEGF9**
**RBL2**	**OTUD1**	**DICER1**	**ZNF616**
**OTUD1**	**DAZAP2**	**TMEM205**	**ADAMTS19**
**TMPRSS11A**	**NDUFB10**	**CBX2**	**DDX3Y**
**MFAP3**	**SCAMP2**	**SDC1**	**ZBTB44**
**NUMB**	**RBM27**	**MLF2**	**CDS2**
**KSR2**	**PEAK1**	**RP4-758J18.2**	**ITGB8**
**UBXN7**	**ANKRD13B**	**EMC10**	**FAM126B**
**POC1B-GALNT4**	**BRMS1L**	**POU4F1**	**ATF2**
**SLMAP**	**FREM2**	**TMEM194A**	**KCTD12**
**SCAMP2**	**TUBGCP3**	**ATXN2L**	**POU3F2**
**MYBL1**	**PCNA**	**C12orf76**	**USP36**
**RBM27**	**SPARC**	**CD59**	**HNRNPA1**
**PEAK1**	**ZSCAN26**	**THUMPD3**	**DICER1**
**SIN3B**	**ETAA1**	**TET1**	**SDC1**
**BRMS1L**	**NAGA**	**YWHAE**	**ZBTB10**
**C18orf21**	**CLPP**	**DENND4B**	**LRSAM1**
**MGAT2**	**UTP15**	**CALM1**	**GAX(hsa)**
**UGCG**	**PTPRK**	**ZNFX1**	**MTERFD2**
**STAT3**	**GALNT7**	**RGS4**	**MMGT1**
**CEBPG**	**UBE2Z**	**VPS13D**	**PRKAA2**
**SFXN4**	**GLG1**	**HIST1H2BK**	**CRKL**
**TNPO1**	**TVP23B**	**SIK1**	**DGKE**
**UBE2Z**	**HSPA1A**	**STOM**	**SLC7A6**
**SWAP70**	**EID2B**	**TMEM167A**	**YWHAE**
**WNT16**	**TGFBR1**	**DAB2**	**TSC22D3**
**G3BP1**	**CHMP7**	**AGO3**	**ELK4**
**ZNF638**	**PDE4DIP**	**DHCR24**	**TMEM59**
**PDCD4**	**TCEB3**	**LENG8**	**CYR61**
**ZNF568**	**COX8A**	**SMIM8**	**TMED7-TICAM2**
**TM4SF1**	**PPP2R5E**	**MEIS2**	**ATP5F1**
**CDON**	**PLP2**	**FAT3**	**GNS**
**ADAMTS5**	**FEM1B**	**ANKRD52**	**CLDN11**
**PDE4DIP**	**RAP2B**	**REST**	**DCTN5**
**TCEB3**	**TROVE2**	**USP31**	**TCF4**
**GGCT**	**YWHAH**	**PLAGL2**	**GTF2H1**
**METTL2A**	**SPRYD4**	**DIDO1**	**GOSR1**
**PPP2R5E**	**CNOT11**	**NF2**	**TMED7**
**UNKL**	**ZNF616**	**PIK3R4**	**PPAT**
**FEM1B**	**ZDHHC21**	**DDX56**	**THBS1**
**LRP6**	**ADAMTS19**	**UBQLN1**	**UBTD2**
**OAZ1**	**GRAMD1B**	**PAFAH1B1**	**ITGA8**
**EIF3F**	**EPCAM**	**REEP3**	**CMBL**

SMC1A	UBR1	PPP1CC	AGO3
MET	MED13L	GNA13	DBN1
ATP6V0E1	RPL6	FAM35A	ETV3
UTP14A	CIC	ZSCAN20	ZNF681
TROVE2	FBXL5	TOMM20	CLCN3
ZBTB11	RPN1	NUP210	TMEM248
WASF1	ZFAND5	SLC25A32	MARVELD1
SENP3	DDX3Y	ZNF16	SSU72
YWHAH	PTGER4	PDE3A	HOOK3
CST5	PLAA	SPRED1	B4GALT5
ZNF273	NDFIP2	PTPN14	ZNF131
BLCAP	GATM	MAP4	RBM28
HLCS	RNF38	KIF2A	UTP6
UBP1	MRS2	FZD6	ZNF275
USP53	ZBTB38	PPP3R1	PITPNA
CXorf23	ZNF827	ASXL1	SGPL1
RRAGD	RB1CC1	WNK1	YWHAG
ITGB1	FAM126B	NME6	SEC23A
ANO6	RHOBTB3	PPRC1	CCND2
C5	RPL7A	SETD1B	PRKCDBP
ZNF32	CDK4	SPATA13	ANXA1
CCNB1	LAMB1	SRCAP	DKK2
HNRNPR	BSDC1	TMEM138	FAM35A
SOCS4	FAM135A	TMEM64	ETS2
GNA12	ATF2	AKAP1	RC3H2
CEP97	NUCKS1	AFAP1	LMBR1
DDX3Y	C12orf49	UBA52	TRIM35
YAP1	RIOK3	ECHDC1	MYOF
ADRBK1	CTNND1	SRGAP1	ABCG2
HMGB3	HDGFRP3	NDST2	NKTR
WASL	SMAD2	RFX1	ARNT
DDHD1	SH3PXD2A	DCBLD2	ETS1
ZNF621	ZNF791	APH1A	DPP8
ELF2	MRPS35	SVEP1	TCF12
SPIRE1	BRD2	AFF4	KCNAB3
AREL1	USP36	HNRNPA0	FRS2
SH3BGRL	USP38	CSDE1	STX3
C1orf43	TDRD1	AXIN1	MEX3A
TRIM23	SCRN1	WDR20	LTBP2
ZIC2	DICER1	MRPL48	FICD
KAT6A	TMEM205	MCU	DNAJC10
ANGEL2	PHB2	ATP1B1	ORMDL3
NUDT12	RBM12	C19orf12	GPX1
DNAJC6	MAST4	SLC25A5	GSE1

PGAP1	SLC37A3	THRA	PARK7
ZNF594	HCN2	SETD5	RCSD1
L1CAM	LRCH1	SFT2D2	SETD1B
FUS	BCAT2	WBSCR22	IRGQ
PLEC	NRAS	TMEM109	TRPS1
PGR	DNAJC3	CD164	RAF1
TCERG1	IP6K1	DSE	FRK
FAM126B	CRKL	BTBD3	ATP7A
SMS	MTHFD2	P4HB	NHLRC3
FAM135A	CD59	ARF1	TMEM64
USP9Y	ATXN1L	CLTC	TIAM2
NUCKS1	DGKE	PTMS	NUP93
GPR137B	REXO1	ZNF225	CCT5
METAP1	PIGX	GLS	GMNN
C12orf49	ELK4	VCL	C18orf32
MFSD6	CLIC4	NOP56	C20orf166
CLASP2	KBTBD2	TIMP2	CPSF6
SHROOM3	CHD1	HN1L	MPZL1
ZNF12	KIF1B	SF1	CDKN1B
RAD23B	HIST1H2AE	ENSA	TMED5
CHCHD1	ATP5F1	MIEF1	PTPRM
EIF2AK3	CLDN11	ANXA2	MYH9
H2AFJ	MBTPS1	NGFRAP1	DSTYK
GARS	BET1L	BANF1	BCAR1
STAM	EXOC5	RAB1A	SNTB2
USP38	DENND4B	ISCU	PHACTR4
ELAVL2	ANKHD1	FOXC1	NUP88
OFCC1	ZMYM4	PRSS23	HIPK3
SH2B3	TCF4	RCAN2	PPP6C
DICER1	LGALS3BP	GPX7	CAND1
ARHGAP11A	INHBB	WDR6	DDX20
IQCG	YOD1	CNOT8	UBE4A
PPP1R9A	WARS	HEG1	HNRNPA0
FBXO21	PPAT	HOMER2	SLC7A11
PAM	ENTPD5	SLC25A40	CDKN2B
CAMK4	RRN3	IPO7	TIAM1
SLC37A3	HMGCR	IGF2BP1	HMGB2
PTBP2	FBXW7	SLC35A4	CLN8
MGAT5	GUCY1A3	OXSR1	TNKS2
ETV6	SSH1	S100A11	ZNF292
NRAS	COL12A1	HERC1	WEE1
IKBIP	WSCD1	NOL9	CDK1
SIK3	ARRDC4	CALM2	SETD5
DIEXF	SIK1	PPIA	SFT2D2
TCTN3	RUNX1	FOXRED2	CD164

CRKL	SLC33A1	APP	LSM14A
LMO2	HSPA4	MARCH7	FMR1
TMEM194A	HMGA2	TAOK1	DDX17
NAT8L	VANGL1	SIK2	ZC3H15
CD59	TOR1AIP2	SLC35A2	KARS
TSPAN5	GTF3C4	AMMECR1L	ODC1
KLF15	UBTD2	SIKE1	KPNA2
CNOT4	ITGA8	SPIN4	ZFHX3
VTA1	PIK3CB	FAT4	ARF1
PPP2R3A	LGR4	DPM2	GEN1
ZNF124	CMBL	NSD1	TMOD3
MRPS23	MMP15	UNG	ZNF226
CRIM1	PIP5K1C	INSIG1	SQSTM1
BRIX1	LENG8	RPS6KA5	GNB1
DUSP11	KIF20A	CBFB	MAP3K4
PRR3	ATMIN	CNP	WWTR1
AP3S1	CDKAL1	TBC1D16	ASPH
KBTBD2	FAM47E-STBD1	FYTTD1	RHOA
CYR61	RECQL	RICTOR	SPATS2L
CHD1	PRKCI	ASH1L	ATAD5
KIF1B	PRRC2B	USP19	GTF2B
LYRM5	TMEM184B	ZSWIM6	CDK5R1
TMEM39A	SETD7	PANK3	UBC
CCNL2	EXTL2	HIF1AN	ZNF510
KIAA0195	CEBPZ	FAM53C	MCEE
CCT7	IFITM1	PNKD	CAV2
LPCAT1	GNPDA1	RNF149	RAB1A
FBXO30	SEL1L	ITSN2	ERBB3
CALM3	ZNF431	RYK	FOXC1
CALM1	CPEB4	CCNT1	QSER1
RFFL	USP31	LAPTM4A	ZKSCAN8
C16orf87	SPTLC2	FAM46A	WDR6
TCF4	FTH1	SLPI	GID8
NUP153	IL10RB	UBA6	CDK6
MAP3K3	NF2	UCKL1	C10orf2
IMMP2L	PIK3R4	VPS4B	HEG1
YOD1	UBQLN1	TTC28	ATP6V1G1
NKX3-2	CTBP1	SOGA1	TFAP2A
IWS1	DPF3	ZNF134	DDX3X
ZNF10	RNF103	WSB2	SERINC1
APC	PAFAH1B1	EML5	CORO1C
HMGCR	DENND1B	NXF1	C1orf35
VPS13D	PITPNA	RASA1	PHF10
OTX2	KLF2	LARP1	CPD
CFL1	SOWAHC	FH	PPIA

COL12A1	DUSP12	MED24	MSN
UBR5	PWP1	PIAS3	SFT2D3
ARF3	ZNF200	CSRP1	FARSA
AFTPH	SGPL1	LRRC59	SRSF2
SIRT1	LMBR1L	CERS2	DEK
THBS1	NEDD4L	MAP3K14	CDK2AP1
USP6NL	YWHAG	TET3	LYSMD1
HMGA2	SEC23A	THRAP3	APP
ZNF780B	MCL1	EPT1	TAOK1
PIK3CB	DUSP6	MAPK9	DST
ATP6V0E2	HLA-E	EIF2A	CANX
ZMYND11	TMEM98	SLIT2	TIPARP
ZNF267	GNA13	ARHGEF2	PDS5A
AGO3	ZDHHC5	IGFBP7	VPS13A
ROPN1L	CCND2	PDSS1	NSD1
RORB	RAB7A	ENO1	RCN2
FAM47E-STBD1	NDNL2	B4GALT1	NCL
TMEM14A	PSMD2	RPL13A	FAM222B
FKBP4	ACTG1	XPO6	CSRNP2
C11orf30	SATB2	TXLNA	C15orf39
AKIRIN2	MARS	MUL1	INSIG1
TPR	TM9SF3	CCND1	PAQR4
ANKRD52	DBT	C10orf12	CBFB
PRRC2B	SLC25A32	CNOT7	CUL4A
SAMD8	ZZZ3	LPAR1	SIX1
SPATA2	SNX30	CTNNA1	ZNF197
TADA2B	STK38	GIGYF2	MBD3
REST	LMBR1	MYLIP	ZSWIM6
GBA2	MAPKAP1	EPHA3	PANK3
PPP2CA	PERP	ASPM	PURB
SETD7	ROCK2	PRRG1	SREBF2
HOOK3	ZNF551	ZNF264	ADAM17
B4GALT5	RECQL5	GCN1L1	EEF1D
SEL1L	ZNF148	NUFIP2	STK39
ZNF431	ZNF45	SEC23B	PDE8B
IPO13	SYPL1	EAPP	TRIP12
CACNB4	ZFC3H1	LRRC58	BCAT1
CPEB4	BACH1	HIF1A	STC1
MIR1199	CASP1	SACS	DYRK2
COPS7B	YPEL2	MAPK8	NFYB
TCF7L2	EXOC6B	PPP2R2A	RELT
MTX3	BCL9L	LNPEP	DCAF7
ZYG11B	GOLGA8B	DESI2	ETV5
ZNF33B	RAD54B	FAM134C	FRAT2
USP1	MAP2K5	IRS4	PPP4R2

TSC22D1	PTPRD	MFSD10	PRR14L
KIF2C	ZNF566	SON	FAM46A
ATG2B	BUB1	HNRNPC	PPP3CA
NR6A1	LCOR	LPL	AGPAT1
KCNQ5	FAM69A	FAM83D	UBQLN2
CDKN3	FZD6	HSPA14	MME
AP2B1	GPR180	WDR36	KIF16B
SORT1	ZNF24	GPRC5A	ARHGAP35
RNF103	TOB1	PTGER2	FOXP1
RSF1	MIEN1	PTP4A2	CNN2
PQLC1	GAN	COG5	NLK
TAF2	KIAA1671	FLRT2	HECTD2
GPAA1	SETD1B	ITPRIPL2	ZBTB37
CDADC1	ARID1B	ZFP36L1	HADHA
KIF3A	IRGQ	C4orf3	PEG10
SEPT11	TRPS1	ARID1A	MLEC
OGT	ITGA5	UBA1	WSB2
IDS	FBXO45	UBAP1	LARP4B
ATAD1	PLCL2	ZNF460	FAF2
ZNF597	EYA4	RRAGA	SF3B6
NAP1L1	MAP4K3	CABLES1	KIF3B
ARL2BP	SLC4A4	ATRX	ZFYVE16
ZNF200	CCT5	COL1A1	RAB11FIP2
IL2RB	IER2	CBX6	HDGF
YWHAG	NDUFAF3	HCN3	DDIT4
SEC23A	BCL11A	RNF19A	CCSER1
PPP1CC	PTBP1	ASL	RIF1
MCL1	ZNF507	CHTOP	CDKN1C
COASY	DBF4	REEP4	CASP3
PTPN4	SELT	ITPR1	ZNF227
DUSP6	CDKN1B	RPRD1B	ZNF711
GNA13	SYNJ1	NEK9	PPP1R15B
FRA10AC1	MYH9	SATB1	PTPRE
CELSR2	DSTYK	RPL5	RNF4
ZFP14	RFX1	DOCK4	TXLNA
MRPL34	TUBB	COMMD10	BAG3
NUDT21	SLC31A1	COPS4	CCND1
COL27A1	MACF1	GLCCI1	LHFPL2
HSP90AA1	ADNP2	NXT2	WDR82
ADSS	DDX6	NRP1	RBM3
ZNF445	ANKIB1	SLC25A36	FNDC3B
RABGAP1	HIPK3	CUTA	MSH6
ZNF260	DLG5	VTI1A	SLAMF6
SATB2	CCDC14	MBD6	PABPC3
HNRNPDL	CASD1	LDOC1L	METTL17

FKBP14	CAND1	SRRM2	MYLIP
TM9SF3	AFF4	SYNE1	RBM17
TOMM20	IBTK	WDFY3	QARS
LSG1	ANKRD17	RAC1	CDV3
TTN	STAG2	SPRY4	PGBD4
RC3H2	EPG5	MESDC2	TMEM19
MERTK	DIRC2	KMT2D	TPD52L2
SLC25A32	CHST9	VPS26B	C1orf198
HNRNPF	CSDE1	SLC25A11	E2F3
ACOX3	TTC17	C6orf89	SMARCA5
EPHA5	NA	ATP6V0C	PAIP2
SSB	WDR20	CS	RGMB
PERP	MEF2D	TNFRSF10B	DPYSL2
HERPUD1	MTMR9	HSPBP1	ANP32B
FAM198B	TNKS2	PLIN5	RQCD1
ARPP19	STAG1	UBXN11	ZC3H7B
AKAP9	MAN2A1	YY1	HERC2
SPRED1	C19orf12	TFDP1	PPP2R2A
SKA2	DEPDC1B	SP1	RNF24
OTUD6B	TECPR2	DUSP7	YPEL3
MYH10	SETDB2	JADE2	LNPEP
ARNT	ZNF292	PDCD11	FAM134C
ETS1	RAB21	KRT80	ACTR2
ZNF850	WEE1	SYNJ2	SUPT16H
HLA-DQA2	SETD5	RRNAD1	PPP1R15A
DYNC1H1	ZNF41	EGLN2	QKI
ZC3H12B	TMBIM1	TNKS1BP1	p27(hsa)
ARHGAP17	CEP85	SERF2	ASXL3
SYPL1	AGFG1	ARHGAP29	EPM2A
BACH1	PPCS	USP42	CTDSPL
SAMSN1	MSL2	REPS2	FBXO3
PAK7	SH3D19	LAMC1	FAM83D
LRRC8D	CD164	MKI67	PRELID1
PRPF39	LLPH	RRP1B	RPS24
AGRN	RAB12	SUMO3	VCAN
FRS2	SSR2	MYADM	ELMSAN1
CBX4	C6orf211	CSNK1E	LIFR
CTC1	UGP2	SLC39A13	ATXN1
ITCH	EVI5	DESI1	ARID1A
BCL9L	ZFHX3	AMOTL2	CLDND1
TOM1L1	GNAI3	CBLN3	AGPS
CAMSAP2	HSPD1	PPHLN1	PPP2R1A
ZNF121	ARF1	GNAQ	GALNT3
DIP2B	LY75	TTLL4	ZNF460
ANKRD50	RAB23	FAM114A1	TFRC

ANGPT2	TARDBP	DPH5	BIRC6
RNF150	CLTC	USP14	KPNA1
SLC25A25	NOTCH2	STARD13	CEP350
SOAT1	SERAC1	CREB3L2	BRAP
PPP3R1	USP47	SCAF11	ARNTL2
SAMD9	ARHGEF10	CKAP4	VCP
TOB1	SNN	RGS16	CHD3
ENTPD6	LUC7L3	NAE1	APLP2
PLEKHJ1	NOP56	HNRNPH1	CNNM4
WAC	NHLRC2	OAZ2	NDUFV3
GAN	ZNF224	ABCA5	HES4
ASXL1	ARFGEF2	DDX19A	ZBTB5
MTHFD1L	XBP1	RC3H1	MIA3
WNK1	PPP2R5C	GLP2R	ERC2
NME6	ITM2B	FBXO44	KIT
GSE1	ASPH	BAHD1	PLIN3
CDC5L	GRAMD3	ZBTB33	TRIM33
MEST	SF1	C5orf24	CREBZF
SEMA3C	ANXA2	CYP1B1	ARIH2
ZFYVE26	ST6GAL2	SCD	PABPC1
TMEM41A	VPS26A	CSTF2	MAP3K10
CA12	TJP1	LETMD1	DHX8
ARID1B	CDK5R1	ZNF644	NOL11
SNAI2	FAM91A1	ATXN2	BAZ2A
MARK2	ASCL2	LASP1	SATB1
ZNF562	APPL1	IPO8	CSNK2A1
HSPA5	TMEM164	EPS15L1	JAK3
MGA	CD2AP	NHSL2	TMCC1
DCST1	RAB1A	CALR	PTPN11
ADO	KIAA0196	BAG6	HSP90B1
CDCA4	PRSS23	RBM12B	PHC2
C14orf169	COPS2	SGPP2	HDAC2
FRK	QSER1	HIAT1	NRP1
CLCC1	HNRNPU	TBP	SLC25A36
BPTF	TCF19	LTBP1	UBE2N
FBXO45	COQ9	KIAA1919	KLHL15
BACH2	SLC16A13	EIF4EBP2	PFDN6
TMEM138	KRAS	VGLL4	RCHY1
LSS	GXYLT1	LDLR	TBK1
PLCL2	CNOT8	DCK	ENTPD1
CHUK	CDK6	RIPK1	LARP4
NHLRC3	VPS37B	DKK1	PTPRJ
TMEM64	KALRN	PPM1B	LDHA
NBN	FOXN2	CREG1	SPRY4
EYA4	FYN	TUB	IFRD1

**MAP4K3**	**THOC1**	**BTBD7**	**CYBB**
**RRM2B**	**GATA2**	**HIPK1**	**CTPS2**
**ZNF664**	**DDX3X**	**AHNAK**	**MESDC2**
**MAP4K4**	**HACE1**	**LEPR**	**FGF2**
**CHL1**	**SETD2**	**UBE2W**	**DNMT3A**
**BCL2**	**ELAC2**	**HERPUD2**	**RNF219**
**AFAP1**	**SLC35A4**	**MFGE8**	**BCL2L2**
**CPSF6**	**ECI2**	**DYRK1A**	**SESN2**
**CDKN1B**	**CNNM2**	**UHRF1BP1**	**C6orf89**
**SLCO2A1**	**HERC1**	**PELP1**	**CS**
**FOXK1**	**WBP5**	**PRDM4**	**MXD1**
**CEP120**	**CORO1C**	**ATP1A1**	**C16orf72**
**SERTM1**	**PHF10**	**TTC9C**	**TXLNG**
**NDUFAB1**	**PRKCE**	**RXRB**	**BUB1B**
**MED13**	**CHD4**	**GTF2A1**	**PSMD12**
**BIRC5**	**ASF1B**	**CD55**	**CLASP1**
**TUBB**	**WDR26**	**RNF213**	**ZC3H11A**
**SLC31A1**	**HRH1**	**QPCTL**	**ERCC4**
**SNX9**	**TRAM2**	**NUP188**	**HMG20A**
**ADNP2**	**ADD3**	**PNRC1**	**RANBP2**
**DDX6**	**CCDC71L**	**MAP3K7**	**SUV420H1**
**RALBP1**	**CELF1**	**WLS**	**ULK3**
**ANKIB1**	**FGFBP1**	**ADSL**	**HIVEP1**
**PPTC7**	**NUDCD1**	**PRDX3**	**DAB2IP**
**ERG**	**RABEP1**	**ABL2**	**NDUFA1**
**HIPK3**	**LBR**	**RPL27A**	**CLP1**
**CCR6**	**CKAP5**	**VPS35**	**RBPJ**
**PPP6C**	**TAOK1**	**CYB5R3**	**SSR3**
**NR4A2**	**SIK2**	**SERTAD2**	**OSTM1**
**CCDC14**	**ERGIC2**	**TIMM10B**	**SLC39A6**
**CAND1**	**UCHL5**	**TMEM136**	**FAM168B**
**TAAR6**	**NPY1R**	**CPNE8**	**PLXND1**
**KLHL5**	**PLAGL1**	**LYSMD3**	**AKIRIN1**
**LIN28B**	**CANX**	**SLMO2**	**NPTN**
**TMX1**	**KBTBD6**	**MAPKAPK2**	**MARK1**
**AFF4**	**KIAA1109**	**STK40**	**TLE3**
**IBTK**	**DCTN2**	**JMY**	**TSPAN17**
**STAG2**	**NCOA3**	**GATA6**	**PEAR1**
**UBE4A**	**RNF128**	**SLC1A5**	**UHRF1**
**UTP20**	**AGPAT5**	**HIVEP3**	**NMT1**
**AGAP1**	**ANK2**	**RAB14**	**SLC20A1**
**STT3A**	**DUS2**	**RFT1**	**PRKAB2**
**PRKCD**	**ZXDA**	**RAB5B**	**LAMC1**
**N4BP2L2**	**NSD1**	**RABL6**	**MKI67**
**DGCR2**	**CCT6A**	**RITA1**	**METTL2B**

MCFD2
MIB1
WDR20
SLC7A11
C9orf16
SUDS3
TMEM257
MTMR9
HMGB2
SP3
TOMM70A
MOB1B
CHRM3
ATP1B1
URI1
C19orf12
CTSD
YWHAB
RNF217
PSMD3
WEE1
SETD5
SFT2D2
DGUOK
PHF3
ZNF736
RCOR1
IVD
EPN2
BLMH
IGF1R
SH3D19
LONRF1
CD164
OSBPL3
MINK1
DDX17
ZNF627
BTBD3
EGFR
C6orf211
HERC3
SRPK2
ANKRD1
PIBF1

PHTF1
NAB1
CHEK1
USP28
SLC35B3
C4BPB
MEGF8
INSIG1
FAM104A
CNP
PRRC2C
RPP14
RICTOR
ASH1L
PANK3
EDEM1
PURB
KLHL11
FADD
USP45
TAS2R30
ARIH1
IARS2
IFIH1
SLC25A38
STK39
BCAT1
DHRS7
FAM20C
CDC42BPG
AMD1
DYRK2
H2AFV
RAB29
SBF1
SNX3
HECTD1
NUMA1
PAPD5
ASAP1
KIAA1524
LAPTM4A
FAM46A
ACAA1
MTR

KRBA1
ZW10
MTF1
HLA-A
ZDHHC7
PLS3
USP34
COQ7
MAP1B
LIG4
YY1AP1
ZNF774
ASAP2
UBOX5
ZDHHC13
TXNIP
PALM2-AKAP2
SMEK2

MIDN
BBC3
HDLBP
ZCCHC14
SLC44A1
DCUN1D1
CHSY1
TAX1BP1
PPP2CB
ZBTB7A
REV3L
RASEF
SEP15
HOXC10
COX15
TOPORS
H3F3B
SEC24B
BRD1
STAMBP
UBE2D1
RBBP7
LYRM4
MFF
KIAA0040
PDGFD
RC3H1
CDC27
KPNA6
GNAI2
MYO10
ZBTB33
SLC6A9
PDE7B
CYP1B1
SCD
SEH1L
VPS53
SDC2
NUF2
NGRN
PCDHGA4
NUP43
KIAA0368
VEZF1

AGO4	PKDCC	TRIM5
CD96	GET4	p27kip1(hsa)
COX7A2L	ATP6V1B2	UBN2
TMOD3	ZNF721	GATAD2B
MSANTD4	KRIT1	SGTA
RAB23	SERP1	ABHD3
PRR5L	ARID2	CTCF
NACC1	VPS4B	GJA1
TARDBP	PRICKLE1	STMN1
CLTC	NPM1	BIRC3
KPNA4	NLK	POFUT1
DNAJC7	DKC1	NAPEPLD
NOTCH2	DUSP10	CCNJ
ZNF519	TRAFD1	HIAT1
CARS	PTGES2	MBNL1
ZNF800	TES	TMEM201
GLS	CEBPB	OXR1
C1QTNF9	ZNF134	SEMA6D
ID4	GTF3C2	ZBTB18
VCL	SETDB1	CSTF2T
PSKH1	WSB2	VGLL4
C6orf48	POLR2D	CAD
CTSC	EML5	SESN3
GNB1	ZNF217	COPA
AMOT	ITGAV	HNRNPA2B1
ZNF625	DCAF6	PER2
MTCH2	MCM2	ZIK1
HAS2	EHBP1	BNIP3L
TIMP2	FBN3	NR2C2AP
ETF1	ITGA11	BCOR
PPP2R5C	SH3BP4	EIF5A2
POLR1D	FH	TUB
HNRNPH3	TAF1	CUL3
MAP3K13	ABCF2	MMRN1
HN1L	KIAA1147	FAM214A
FAM175B	KIF3B	HIPK1
CHD7	NRF1	AHNAK
SH3GLB1	CCAR1	YES1
TJP1	TMX4	AP1G1
UBE2J1	MAL2	PAFAH1B2
FAM91A1	CNIH1	EHD2
ZNF510	PHTF2	TMEM127
TMEM164	NDE1	ZNF367
CD2AP	CDH1	MTOR
RAB1A	DENND5A	ZFP36L2

PDCD6	KIAA1432	OSBPL8
CFI	PITPNM2	ZNF106
PSAP	SFXN1	ABCC5
COPS2	CUL5	NKRF
IRF8	NFAT5	DYRK1A
EIF1AX	PIKFYVE	FUT8
STT3B	PPP1R12A	KLHL24
HNRNPU	AEBP2	CTDSPL2
MRPS14	KLHDC10	LEPROT
HSDL1	ZBED6	DPH2
ZKSCAN8	PLEKHG3	UBXN2B
UBE2I	NECAP1	ACIN1
MAGED1	PKP4	ATP1A1
KRAS	CLIP1	SUN2
GXYLT1	GANAB	NAPA
WIZ	BTG2	TRIM71
SPRYD7	FBN1	C9orf156
RSL1D1	PPP1R15B	PTEN
EPDR1	HIST2H2AA3	ZNF652
HEG1	DCP2	NFATC3
HEY2	FBN2	TRAM1
IPO7	RNF4	FERMT3
WBP2	TXLNA	ISCA1
DDX3X	TMEM173	SLC35F6
PDIA6	GOLGA8A	ALDH1A1
IGF2BP1	CCND1	PPFIA1
MDH2	N4BP2	FNDC3A
COPB1	LHFPL2	SDCBP
GOLGA6L4	FNDC3B	NUPL1
FSTL1	C2CD5	SUCLG1
INIP	NRXN3	TET2
EYA3	CTNNB1	USP18
TCEB1	ZNF805	SLC40A1
ZNF189	AP3B1	SCARB2
MTX2	EFR3A	AP3M1
ARPC1B	MMADHC	KMT2C
PHF10	LPAR1	HELLS
FBL	ANXA7	HOXA10
SLC35A5	PTBP3	RNF26
TCP1	ASNS	SERBP1
ACOT12	PMM2	DVL2
FKBP1A	CCNE2	TAF3
CALM2	AXIN2	ARF4
PRKCE	PLEKHB2	ZCCHC24
SMARCD1	NR3C1	MRPL19

CPD	SSFA2	BRWD1
ZBTB41	SBNO1	TBL1XR1
BUB3	GIGYF2	ERO1LB
MTMR12	KCTD2	ZNF91
ZCCHC2	MYLIP	HYOU1
SNIP1	TRAPPC9	BZW2
SRSF2	ASPM	SECISBP2L
SLC35C2	CDV3	EIF1
MORF4L2	ZNF264	PTCD3
WDR26	FAR1	TFG
ZNF107	AXL	MAML1
EPC1	NUFIP2	ZFAND3
DEK	TMEM87A	TSPAN13
ADD3	MSL1	ARHGAP5
PRPF8	LRRC58	RAPH1
CELF1	ADM	RAB5B
PDS5B	DYNC1LI2	MDM2
SRSF7	GIT2	PSMB5
FBXL7	E2F3	PGK1
SLC9A6	SMARCA5	ZNF629
CKAP5	SACS	UBE2J2
TAOK1	ATP5A1	TAF6L
TFPI	GDAP2	BMPR2
ERGIC2	DMXL1	TMBIM6
DST	KPNA5	TOR1AIP1
KIAA2026	COX5B	ATF4
SUMF1	RQCD1	BCL2L11
RBAK	DNAJB9	DUSP19
KIAA0101	CGGBP1	MTF1
FOXK2	DNAJC13	LIN7C
ADIPOR2	NEFH	PLS3
ABI3BP	CHCHD10	PIN4
KIAA1109	DPY30	USP34
HK2	LNPEP	MIER3
NCOA3	AFF3	SLC30A1
C3orf14	ZNF44	UBR4
PKNOX1	IRS4	NACC2
ZNF622	MYC	TCTEX1D2
LPP	JKAMP	FNIP2
TMEM123	NR2C2	ANAPC16
GIGYF1	CENPF	KDM5B
ATP2B1	IKZF5	EFNA1
GPR183	SON	ELF1
NSD1	HCFC1	ACACA
TMEM184C	IQGAP2	MLLT6

CCT6A
PLEKHA8
RNF220
PIP4K2A
ATP5B
PRCC
NAB1
RPLP1
RFC2
MYCBP2
ZDHHC3
USP28
RBM38
SLC16A3
POLR2K
MPP5
FOXB1
INSIG1
RALGAPB
MFHAS1
ZNF571
TP53
TNRC6B
RNF187
SPTY2D1
LRIG2
ZNF543
LITAF
LEMD3
GLTSCR1L
DDX21
ZNF197
RICTOR
ASH1L
MBD3
ADAMTS2
ZSWIM6
EGR3
PANK3
MYNN
PURB
KLHL11
ACSS2
AMMECR1
LCP1
RAPGEF6
MARCHF8
TXNRD1
PPM1A
BTBD2
VEZT
MTMR1
DEPDC1
WSB1
INSIG2
ELMSAN1
HIST1H3D
GPAM
MRPS2
OCIAD1
PGBD2
C21orf91
TMEM9B
ARPC2
GOLGA4
GPBP1L1
KLF3
ATXN1
FAM49B
STK35
HNRNPA1L2
PCBP1
YARS
ATP10B
CPNE3
MTPN
ZNF460
TFRC
JRKL
TMED10
CCDC47
SRPR
ARNTL2
ZC3H12C
SYT1
XPR1
MAFK
AARS
ZNF687
APLP2
ZNF749

SREBF2	CNNM4
ZDHHC12	CR1
HDHD1	PRNP
PRDM1	FBXO10
ARIH1	MYO5A
ADAM17	SLC38A2
UBA2	MYCBP
ZNF432	MUC5B
PDE8B	ZNF100
TRIP12	MIA3
BCAT1	ABHD5
DHRS7	ZC3H13
SIPA1L1	MED31
AZIN1	DEPDC4
ATP9A	CERK
AMD1	XYLT2
POSTN	NOL4L
DYRK2	HNRNPM
ZNF317	CHTOP
ZNF253	SMAD5
CREB1	PSMC4
TSG101	MMD
SORBS3	GTF3A
RBMS1	SF3A2
ZMYM6	NCOA2
PPP4R2	ITPR1
CCSAP	RBM47
DSCR8	DKK4
SLFN11	TCF20
FAM122B	AP1S3
C7orf73	NEK9
CHPF2	SREK1
ARL8B	BAZ2A
HECTD1	SKI
PRTG	TGIF1
APAF1	PRDM15
PAPD5	SOS1
MRPS5	ADPRHL2
MAPK1IP1L	CPEB2
GREB1L	UBR2
BPGM	RORA
ACVR2B	UMOD
EDEM3	KIAA0284
GABBR2	SLC25A36
RAB5A	ZNF184

**MTR**	**SRRM2**
**UGT3A1**	**GEMIN5**
**PCGF5**	**METTL10**
**UBQLN2**	**KLHL15**
**KRIT1**	**FXR1**
**ZNF136**	**PRODH**
**HSD17B3**	**DCUN1D3**
**GADD45G**	**METTL21A**
**EIF5**	**SLC26A2**
**ARHGAP35**	**LYST**
**PTK2**	**CUX1**
**SLC2A3**	**PTPRJ**
**ST3GAL5**	**XRN1**
**SLC25A30**	**S100A10**
**FBXO34**	**C6orf62**
**SLC9A3R1**	**DUSP8**
**PTPN13**	**NOLC1**
**IDI1**	**SHOC2**
**LCMT1**	**AIMP1**
**TRUB1**	**MCUR1**
**FOXP1**	**SNAPC1**
**SOGA1**	**SH3RF1**
**DKC1**	**ANKRD46**
**FURIN**	**TRIO**
**TES**	**PIK3C2A**
**HECTD2**	**RDH10**
**ZNF134**	**C16orf72**
**TICRR**	**PAWR**
**ZBTB37**	**DSTN**
**PNPT1**	**PUS7**
**ETNK1**	**FAM199X**
**PEG10**	**TXLNG**
**STX7**	**TMEM2**
**MOB3B**	**PPP1R12C**
**MLEC**	**LCLAT1**
**ARHGAP18**	**NF1**
**AP000350.10**	**FAIM**
**ZNF232**	**ZNF430**
**CADM1**	**STRN**
**LARGE**	**MADD**
**BRCA1**	**CDK13**
**ZNF217**	**DDX55**
**DIP2C**	**RANBP2**
**KLRC4**	**SUV420H1**
**LARP4B**	**ZFP1**

NAA20	CXXC5
PSD3	HIVEP1
RASA1	SP1
NAA50	DAB2IP
LIF	RARS
ZNF268	LURAP1L
PTCD1	COL1A2
CPT1A	NDUFA1
LDLRAP1	RBPJ
ZNF697	SYNPR
DMWD	PSMB1
TBX4	LRPAP1
PRCP	BCL6
DERL2	GOT2
DOCK10	OSTM1
CSRNP1	FNIP1
CSE1L	SMIM13
FKBP7	FAM168B
NA	NPTN
CNIH1	CDC23
PHTF2	PTPLAD1
PMAIP1	RASSF3
ANP32A	YBX1
URB1	ARRDC3
YIPF4	NFE2L2
MRPS18B	IKZF2
SMC4	PSMA3
DDIT4	PGRMC1
SFXN1	SLC20A1
CUL5	FOXJ3
RBM39	MKI67
TET3	FXR2
NA	PRKAR1A
DROSHA	BARX2
ZBED6	FAM160B1
LFNG	EP300
RIF1	SUN1
SF3B3	MGEA5
AGO1	ARL5B
NECAP1	ZCCHC14
TMSB4X	PCDHB13
EPT1	GNAQ
OGDH	RASSF9
SGMS2	PUM2
CRYBG3	NFIB

**PCNX**	**AHR**
**TTC37**	**REV3L**
**C1orf52**	**CELSR3**
**KHSRP**	**ATF7IP**
**CLIP1**	**BRD3**
**NOTCH1**	**GOLGA1**
**GANAB**	**TEF**
**POLR2A**	**YIPF5**
**ZNF26**	**CNOT1**
**CENPI**	**UBE2D3**
**IGFBP7**	**OSGIN2**
**FBN1**	**SLC9A7**
**ENO1**	**CREB3L2**
**B4GALT1**	**ATXN7**
**ZNF227**	**SCAF11**
**AK6**	**AMIGO2**
**ALG10B**	**VPS41**
**JUN**	**CTSB**
**ZNF711**	**H3F3B**
**PIK3R3**	**ARMC1**
**PPP1R15B**	**CERS6**
**RPL13A**	**UBE2D1**
**WDR33**	**TARBP2**
**CCT3**	**C11orf24**
**DCP2**	**G3BP2**
**ATP6AP2**	**PCDH7**
**CLINT1**	**ZNF730**
**ZNF530**	**FAM210A**
**LRCH2**	**SNX13**
**XPO6**	**CDC27**
**NCS1**	**KPNA6**
**FAM208B**	**IGFBP3**
**SLC20A2**	**ZNF211**
**CCND1**	**LONRF2**
**IMPAD1**	**MYO10**
**ILF3**	**ABI1**
**N4BP2**	**NTN4**
**PFN2**	**MED23**
**LHFPL2**	**MARCHF5**
**URM1**	**UBE2G1**
**PPARA**	**SDC2**
**WDR82**	**CSTF2**
**FNDC3B**	**MYEF2**
**C2CD5**	**PAPD7**
**JUNB**	**MIS12**

**ICMT**	**PCMTD1**
**CTNNB1**	**AURKA**
**AP3B1**	**IL6ST**
**EFR3A**	**ATG5**
**HMBS**	**LEPREL2**
**ZNF678**	**DNAJB12**
**SOWAHA**	**THOC2**
**PUM1**	**NUP43**
**ACTR3**	**PLEKHA1**
**LPAR1**	**FAM102A**
**SPEN**	**AKR1A1**
**PTBP3**	**VEZF1**
**S1PR1**	**MYO5C**
**ZNF236**	**NSF**
**GFPT1**	**UBAP2L**
**GGPS1**	**MED19**
**PLEKHB2**	**CTTNBP2NL**
**CHRFAM7A**	**RLF**
**NR3C1**	**ZNRF2**
**E2F5**	**NDC1**
**SEC24C**	**SLC7A2**
**STRBP**	**FGD4**
**SBNO1**	**TULP4**
**NCOA6**	**AMACR**
**MPHOSPH6**	**FMN2**
**BHLHE40**	**IPO8**
**ARL6IP1**	**GATAD2B**
**IFFO2**	**CTCF**
**CDV3**	**COG3**
**DOCK1**	**RNF182**
**ZNF264**	**CDKN1A**
**C1orf131**	**KIAA0232**
**FAR1**	**DOCK9**
**TSR1**	**MDN1**
**SEC23B**	**SLC2A1**
**ST8SIA4**	**USP9X**
**BLOC1S6**	**WDR4**
**SLC19A2**	**ZFYVE21**
**EAPP**	**WDR43**
**IGFBP4**	**RP2**
**LRRC58**	**PDXDC1**
**SLC4A7**	**CNEP1R1**
**ADM**	**KIAA1919**
**TRAK1**	**MRPL44**
**MTA1**	**ZBTB18**

DYNC1LI2	GFPT2
CISH	SMG7
GATC	SESN3
HELZ	SLX4
ESRP2	RCOR3
POGZ	TMEM25
SACS	NOP9
C2CD2	PER2
ZNF443	ATXN3
SMCHD1	DKK1
ATP5A1	PPM1B
CEP41	CCDC186
ICE2	TNPO2
UBE3A	BCL11B
ATL2	SUGT1
ZNF101	GAREM
PPAP2B	TUB
KTN1	AIFM1
RIT1	ACPP
CSNK1A1	GID4
CREBRF	CACNA2D1
DNAJC13	ATP2A2
LRRC17	MPPED2
RNF24	HIPK1
LNPEP	AHNAK
CEBPA	RINT1
F2R	RGL1
WDR48	AP1G1
MED26	SMAD7
MNDA	PAFAH1B2
QKI	LEPREL1
TMC5	CEP55
UBE3C	RAB8B
NR2C2	UBE2W
CENPF	ABCA1
SON	RAN
PIM3	HERPUD2
CD46	DUSP5
AHCTF1	GDI2
CTDSPL	JADE1
ZNF626	SLC12A5
TMEM14C	OSBPL8
MKLN1	PLEKHA6
PPM1A	USP11
MYO9A	HIST1H1B

**HSPA14**	**NCKAP1**
**PRELID1**	**ANAPC13**
**RASSF6**	**DYRK1A**
**SFXN3**	**SNX25**
**WSB1**	**MOAP1**
**SRSF6**	**CTDSPL2**
**ELMSAN1**	**PTAR1**
**HIST1H3D**	**STEAP3**
**MLH3**	**PRDM4**
**FXYD6**	**DLST**
**LIFR**	**HIST2H2BF**
**TMEM9B**	**CAMK1D**
**ZFP36L1**	**KIRREL**
**COL5A1**	**ITGA6**
**DBNL**	**SPIN1**
**ARV1**	**SGPP1**
**SMAP1**	**PDZD8**
**GOLGA4**	**MAP2K4**
**GPBP1L1**	**SAMD9L**
**BLOC1S5-TXNDC5**	**RPS6KA4**
**KLF3**	**XRCC5**
**ATXN1**	**CHKA**
**FAM49B**	**RSBN1**
**CCDC82**	**RAB11FIP1**
**DEGS1**	**ATP8B1**
**DENND5B**	**GTF2A1**
**ZFAND1**	**TANC2**
**PCBP1**	**XPO1**
**YARS**	**EPHA4**
**BEND4**	**RRBP1**
**MSH2**	**GPBP1**
**ZNF132**	**PHIP**
**CPNE3**	**CPEB3**
**CENPO**	**MUT**
**EIF4A2**	**NFATC3**
**MTPN**	**DUSP16**
**SLC35A3**	**FDPS**
**ZNF460**	**PALLD**
**TFRC**	**SGK3**
**ZMIZ1**	**POLR1A**
**MCTP1**	**LIN54**
**BIRC6**	**PLEKHM1**
**FLNB**	**HDHD2**
**ADAM12**	**DEAF1**
**DAPK1**	**GRHL1**

**TMEM241**	**RAD17**
**TMED10**	**C16orf62**
**ZAK**	**ATP13A3**
**ATAD2B**	**RNF141**
**SETD8**	**SLAIN2**
**KPNA1**	**TLN1**
**TSHR**	**GPR126**
**AAR2**	**MAPK6**
**CEP350**	**LATS2**
**FRMD6**	**PTGFRN**
**KIAA1462**	**CREBBP**
**ZC3H12C**	**ZNF146**
**COL1A1**	**PRDX3**
**ANKRD44**	**ABL2**
**ZMYM1**	**FGF7**
**KCTD3**	**TRAF3**
**ZNF558**	**WRNIP1**
**C6orf106**	**USP33**
**ANKLE2**	**ZHX1**
**ZMYM2**	**RAD21**
**APLP2**	**TET2**
**FLT1**	**PCGF3**
**SLC11A2**	**DNAJC27**
**BEND3**	**KMT2C**
**SMNDC1**	**CDK16**
**C8A**	**MGAT4A**
**RPLP0**	**GOLGA3**
**SLC38A2**	**SERTAD2**
**NCOR1**	**TRMT10C**
**AK3**	**VAT1**
**ZNF180**	**SLFN5**
**ARRB2**	**PRSS12**
**NIPA2**	**FAM129A**
**UCHL1**	**ENTPD4**
**ULK4**	**KLF6**
**CEMIP**	**ARF4**
**ZNF812**	**RANBP6**
**C8orf33**	**TMEM74**
**TMEM87B**	**KIAA1279**
**SAV1**	**PDK2**
**VASH1**	**RNF139**
**CTD-2228K2.5**	**ATP5J**
**JMJD1C**	**ZNF470**
**RNMT**	**KPNB1**
**RBM26**	**CBX5**

SMAD5	KLF4
ZNF175	TNKS
C5orf51	JMY
NCAPG	TBL1XR1
UBE2Q1	WDR37
TRIM33	SETX
CREBZF	ZNF91
TGOLN2	BAZ2B
PTGS2	NR2F1
DENR	GATA6
AGGF1	TIMM17B
HECA	ZFP30
PATZ1	PIGA
NCOA2	EIF1
ARL6IP6	GLYR1
PABPC1	IRF1
ITGA2	ADRB1
MAP3K10	SGK1
SEC24A	PRRC2A
PIK3R1	MARCHF3
ZNF426	MBOAT2
NEO1	NAA38
RB1	RBM15
PNRC2	MDM2
AP1S3	BAG5
LRRC8C	GPR137C
AEN	PRR11
BCAP29	TNRC6C
BAZ2A	FANCI
SLC35E1	AP5M1
MARCKSL1	VPS54
CDKN2AIP	FAM110C
KDM5A	VKORC1L1
TXNDC12	RPS29
ARL4D	PNISR
TMCC1	ATF4
RORA	EIF4G2
SLC22A23	BCL2L11
HSP90B1	CLOCK
CDC42SE2	KIF5B
ZFAND6	MTF1
FARP2	SOBP
EIF4ENIF1	MYO1B
GLCCI1	LIN7C
NRP1	CDC42BPB

SLC25A36
NAA30
UBE2N
MBD6
GDI1
TMPO
SRRM2
RAD18
KLHL15
ATF7IP2
NUAK2
ZNF506
TBK1
JAZF1
GNPNAT1
DDX5
EIF5B
LARP4
RIMKLB
SEC31A
MIF
BACE2
XRN1
NIF3L1
C6orf62
ANKRD13C
OTUD4
STC2
ZNF823
MAPK3
AQR
ANKFY1
PTP4A1
SDAD1
PRR4
DNMT3A
TCF21
PMPCB
KMT2D
ADAMTS1
SS18L2
MAP7
NUP50
IPO5
SESN1
NDUFB4
PAX3
CD44
KCTD20
NPTX1
ATR
MAP1B
CTGF
MIER3
CD69
MDH1
DNAJC30
COL4A1
TCEA1
NUDT19
UBR4
ZKSCAN1
LIG4
BSN
PPP1CB
FNIP2
IDH3G
KDELR2
AGPAT9
TXNIP
MRPS21
HIST1H2AH
NEFL
ANKRD18A
SRGAP2
HNF1B
MARCKS
SLC6A14
HIST1H2BH
TAF15
RNF115
MYO18A

MCUR1
BCL2L2
TXNDC15
STX12
C6orf89
TMED4
ZNF14
CITED2
PHYH
DSC3
HILPDA
CARD10
CHD2
C16orf72
PAWR
INTS6
RAD1
LYRM1
IGF2BP3
P2RY1
ELAVL1
ADCK3
SSX2IP
MIOS
SNRNP40
ZBTB21
DHX15
BAG2
CCDC50
CLASP1
CALD1
ZC3H11A
HSPA8
LCLAT1
NF1
FIGN
CCNY
ZNF700
STRN
MADD
SMARCD2
KIAA0100
EIF2S1
RANBP2
DSG2

GPR83
RAB9A
YY1
SRP72
USPL1
INCENP
FAM98A
RBPJ
INPP1
MDM4
LARS
CD93
RNF146
SSR3
BLOC1S2
PHOX2A
ACVR1C
SLC39A6
SMIM13
FAM168B
PROSC
ONECUT2
GTPBP2
AKIRIN1
NPTN
MAT2A
PTPLAD1
ZNF451
TRPC4AP
MARK1
AP3D1
TSPYL4
PEAR1
TAF7
ARRDC3
EPC2
GFM1
ZNF788
SLC20A1
ARID4B
LAMC1
USP24
RAI14
C15orf57

NFE2L1
CNPPD1
FOXJ3
SLC22A15
GSTM2
RRP1B
TGFBR3
APEX2
VMA21
FAM160B1
MTRR
CD200R1
EP300
MIDN
AMOTL2
DDAH1
GHITM
BTG3
ZNF35
TMEM245
ARL5B
ZCCHC14
ARFIP1
GPCPD1
FAM73A
CDK17
DCAF17
PLAG1
PUM2
ZNF780A
NFIB
THUMPD1
TWISTNB
AKT3
RSBN1L
SPDL1
ZNF511
ZNF484
PITPNB
PIAS2
ARHGEF15
TEF
YIPF5
CNOT1
ZNF281

CEP135
UBE2D3
FSIP1
USP14
WHSC1
OSGIN2
TFB1M
SCAF11
BCL7A
KBTBD3
H3F3B
PTPRZ1
ZNF564
BRD1
WNT3A
CD4
GAPVD1
DDX39B
G3BP2
RBBP7
SEC22A
CPOX
MBD4
SMC2
ZFR
ZNF730
ARCN1
CCNE1
RC3H1
ZNF440
CDC27
KPNA6
GNAI2
TUSC1
FASTKD5
LRRN3
LONRF2
MYO10
SPG21
CEP57
C5orf24
ATP6V1C1
PPP1R3B
CYP1B1
SCD

LAPTM4B
ACSL4
UBE2G1
C14orf28
RLIM
ZNF644
SEMA7A
THOC2
NUP43
PLEKHA1
FAM102A
ZNF704
KIAA1244
KMT2A
RPN2
LGALSL
UBAP2L
ZBTB48
TRIM21
ALDH9A1
GRPEL1
RLF
IGSF3
ZNRF2
p27kip1(hsa)
CMC2
NDC1
SLC7A2
SEPT2
ZNF570
UBN2
CDIPT
CTCF
TMEM45A
PKN2
USP7
COG3
AKAP12
GSDMD
MCCC2
CALR
ARHGAP11B
TMEM30A
TEAD4
ZNF92

MAP2K1
STMN1
UHMK1
PKD2
CCNJ
OSBPL1A
ATP8B2
MFSD8
NANS
JRK
CHTF8
TNRC6A
MAP3K2
ARF6
RSPRY1
C1orf109
USP9X
MBNL1
WDR4
HEXIM1
NFIA
ZNF66
DNAJA4
ZFHX4
TRMT1
CNEP1R1
AVL9
UBR3
ARSJ
BHLHB9
GSG2
MAP3K8
ELOVL6
SSBP2
ZBTB18
PPA1
CARM1
EIF4EBP2
HIGD2A
SMG7
RPS14
SLC39A10
TGIF2
SESN3
COPA

RCOR3
KLHL12
FAT1
C17orf51
HNRNPA2B1
NAA25
PDPK1
PRDX6
RPRD1A
PER2
HK1
ATXN3
ZNF483
TAF15
ZBTB1
ADARB1
TTI1
ZBTB4
HMGCS1
SUGT1
BNIP3L
BARD1
WNT2
TNFRSF6B
NDUFS5
PNMA1
CCNI
MMRN1
BTBD7
FAM63B
RUFY2
FAM214A
CKS2
ATP2A2
PCBP2
STAT1
HIPK1
ENY2
AHNAK
TRPM7
YES1
YTHDF3
PRKDC
SMAD7
PAFAH1B2

ORC6
SRGN
BVES
ZNF717
CEP55
RAB8B
UBE2W
TCL1A
TMEM127
RAN
MKNK2
RHBDD1
ZIC5
ZNF367
GDI2
TRRAP
ZFP36L2
TMEM192
OSBPL8
NMRK2
ZNF106
KIAA1467
NCKAP1
TTC27
PSMD8
PSMA4
GPR157
ADK
NFYC
AGPAT3
PLXDC2
NKRF
ULK1
ABHD17C
SKA3
CTDSPL2
ATG10
EXOC8
MOB1A
HSPA1B
CLUAP1
VCPIP1
IL1A
SPIN1
SUCO

UBE2B
CNOT6L
TACSTD2
UQCRQ
CLSTN1
ZNF22
HOXA11
TMCO3
ZNF699
DNAJA2
B3GNT2
CCNC
ZNF618
ATP8B1
GTF2A1
ZNF77
XPO1
TRIM71
FAM171A1
FBXO33
SELK
TIGD2
PTEN
ZNF652
NUB1
MTF2
EWSR1
MARCHF6
SNX4
SIL1
SPTBN1
ATL3
LIN54
ISCA1
LATS1
FAM160A2
S100A13
C9orf91
CAP1
ALDH1A1
ADAMTS9
DCAF16
PCYOX1
MAPK1
NUP188

NIPBL
FNDC3A
SLAIN2
MICAL3
MAPK6
MMP14
PTGFRN
ZNF561
ZNF146
PRDX3
ABL2
ZNF772
RFC3
USP33
H1F0
RTEL1
SERPINE1
USP18
FAM73B
PLAU
KMT2C
TBPL1
GRB2
ADCY9
CALCRL
CCNG2
HELLS
GCNT1
GTSE1
BTG1
RBM6
HOXA10
SERTAD2
CAPRIN2
SMAD1
VBP1
PARD6B
AVEN
ZNF567
CBX3
DVL2
MTCL1
LYSMD3
DIS3
GNB4

ZNF766
ENAH
AKNA
TNPO3
ANKRD10
ZNF675
KNTC1
RBM4
KPNB1
ABHD13
FDX1L
RHOB
ZNF30
BRWD1
CBX5
TBL1XR1
ERO1LB
WDR37
ZNF91
LETM1
GATA6
ZFP30
ATRN
CCP110
GOLPH3
ASXL2
LYPLA1
TGFBR2
COLGALT1
SCN8A
EIF1
GLYR1
CLEC16A
FERMT1
ZNF529
ZFAND3
RPL15
SNRNP200
MARCHF3
MBOAT2
GCNT2
RBM15
PCDHB8
CAMK2N1
GLYCTK

DYNLRB1
PGK1
SLC30A6
NIN
LPGAT1
ZNF629
TNRC6C
SCML1
NIPA1
FAM98B
VKORC1L1
S100A1
PLA2G4C
BMPR2
KIAA1551
DMXL2
FAM169A
PNISR
ZNF565
ZNF703
KBTBD7
ZMYND8
MAP3K5
CCDC6
AJUBA
ZDHHC7
SOBP
EPB41L3
LIN7C
SIN3A
PLS3
BBS7
USP34
ARHGEF28
KCTD20
XIAP
MOSPD2
MAP1B
EPB41
NOL4
TMF1
CTGF
MIER3
ADH5
CAMTA2

DHX33
PPP1R3D
GSTCD
NUDT19
UBR4
ZKSCAN1
NACC2
FKBP10
PPP1CB
C2orf69
BNIP2
NCALD
SPRY2
MEX3C
YTHDC1
EIF3I
INO80D
PANK1
CUL4B
KDELR2
SIX6
GSPT1
AGPAT9
RMND5A
OR11A1
C7orf55-LUC7L2
ZCCHC3
TXNIP
GGNBP2
PIP4K2B
MT-ND2
ZNF229
PALM2-AKAP2
ZNF25
GDPGP1
PCGF2
GPR75-ASB3
RASL10B
SYNRG
TAF15
CTDSP2
ORAI1

miR-151a-5p	miR-221-3p
BMI1	BMI1
EFNB2	EFNB2
ZNF503	ZNF503
IRS2	IRS2
CDC6	CDC6
ACTB	ACTB
DUSP4	DUSP4
PRLR	PRLR
MSI2	MSI2
EGR1	EGR1
ACKR3	ACKR3
BICD2	BICD2
ADIPOR1	ADIPOR1
RNF44	RNF44
SOX4	SOX4
OLFM1	OLFM1
RIC8A	RIC8A
DAZAP2	DAZAP2
NFKBIZ	NFKBIZ
NAT1	NAT1
TXNL1	TXNL1
C18orf21	C18orf21
G3BP1	G3BP1
TCEB3	TCEB3
PLP2	PLP2
ATG13	ATG13
SNX11	SNX11
SMC1A	SMC1A
FSTL3	FSTL3
RP4-695O20__B.10	RP4-695O20__B.10
HNRNPR	HNRNPR
SOCS4	SOCS4
RIPK2	RIPK2
PLEC	PLEC
TM9SF1	TM9SF1
RAPGEF1	RAPGEF1
BRD2	BRD2
PHB2	PHB2
MAST4	MAST4
H2AFX	H2AFX
LPHN2	LPHN2
SCAF4	SCAF4
ZNF532	ZNF532

**MIER2**	**MIER2**
**ZBTB42**	**ZBTB42**
**SIK1**	**SIK1**
**GTF3C4**	**GTF3C4**
**FAT3**	**FAT3**
**WDR41**	**WDR41**
**VAC14**	**VAC14**
**PITX1**	**PITX1**
**BNIP1**	**BNIP1**
**KDM4B**	**KDM4B**
**RAB7A**	**RAB7A**
**ANXA1**	**ANXA1**
**PPIL1**	**PPIL1**
**ADSS**	**ADSS**
**RC3H2**	**RC3H2**
**MYOF**	**MYOF**
**HUWE1**	**HUWE1**
**MAP4**	**MAP4**
**ETFDH**	**ETFDH**
**TOM1L1**	**TOM1L1**
**STX3**	**STX3**
**COLEC12**	**COLEC12**
**IQGAP1**	**IQGAP1**
**CDC5L**	**CDC5L**
**CDH11**	**CDH11**
**OCLN**	**OCLN**
**ZNF620**	**ZNF620**
**APH1A**	**APH1A**
**DDX20**	**DDX20**
**OTUB1**	**OTUB1**
**CSDE1**	**CSDE1**
**LAPTM5**	**LAPTM5**
**SRM**	**SRM**
**PRPF19**	**PRPF19**
**ZNF292**	**ZNF292**
**KLHL21**	**KLHL21**
**CEP85**	**CEP85**
**BLMH**	**BLMH**
**IFITM3**	**IFITM3**
**ARFGEF2**	**ARFGEF2**
**FEM1C**	**FEM1C**
**TAB2**	**TAB2**
**SPRED2**	**SPRED2**
**SLC7A5**	**SLC7A5**
**RSL1D1**	**RSL1D1**

PES1	PES1
CDK6	CDK6
EPDR1	EPDR1
MAP3K9	MAP3K9
RHEB	RHEB
ZC3H3	ZC3H3
FBL	FBL
CXCL2	CXCL2
PPIA	PPIA
EPC1	EPC1
CANX	CANX
DPP9	DPP9
ADIPOR2	ADIPOR2
SKP1	SKP1
ZNF57	ZNF57
TNRC6B	TNRC6B
PRRC2C	PRRC2C
HSPB1	HSPB1
HIVEP2	HIVEP2
SREBF2	SREBF2
MED1	MED1
RNF40	RNF40
ZNF207	ZNF207
TRIP12	TRIP12
RALGPS2	RALGPS2
CCNT1	CCNT1
SLCO5A1	SLCO5A1
PRR14L	PRR14L
RBM8A	RBM8A
SLC7A1	SLC7A1
ZNF721	ZNF721
SPINT2	SPINT2
XPO7	XPO7
PEG10	PEG10
FAM192A	FAM192A
NXF1	NXF1
LARP1	LARP1
NFAT5	NFAT5
EEF2	EEF2
NOL6	NOL6
PPP1R15B	PPP1R15B
BABAM1	BABAM1
BAG3	BAG3
CTNNB1	CTNNB1

**TAPBP**	**TAPBP**
**NONO**	**NONO**
**WWC3**	**WWC3**
**NUFIP2**	**NUFIP2**
**TSR1**	**TSR1**
**SMCHD1**	**SMCHD1**
**ATP5A1**	**ATP5A1**
**PKMYT1**	**PKMYT1**
**MDGA1**	**MDGA1**
**RNASEK-C17orf49**	**RNASEK-C17orf49**
**MYC**	**MYC**
**FAM83D**	**FAM83D**
**CEP72**	**CEP72**
**CCT6B**	**CCT6B**
**PTGER2**	**PTGER2**
**LBH**	**LBH**
**HOXA3**	**HOXA3**
**RNASEK**	**RNASEK**
**COL5A1**	**COL5A1**
**ARID1A**	**ARID1A**
**E2F6**	**E2F6**
**FOXA1**	**FOXA1**
**PRKACA**	**PRKACA**
**XPR1**	**XPR1**
**DUT**	**DUT**
**HSP90AB1**	**HSP90AB1**
**ARHGDIA**	**ARHGDIA**
**SLC38A2**	**SLC38A2**
**GABARAPL1**	**GABARAPL1**
**DDB1**	**DDB1**
**EIF4G1**	**EIF4G1**
**RAB11A**	**RAB11A**
**MIA3**	**MIA3**
**COPZ1**	**COPZ1**
**SLC39A9**	**SLC39A9**
**RHOH**	**RHOH**
**KCMF1**	**KCMF1**
**ARL6IP6**	**ARL6IP6**
**MEPCE**	**MEPCE**
**TEAD1**	**TEAD1**
**TXNDC12**	**TXNDC12**
**NXN**	**NXN**
**GDI1**	**GDI1**
**ZNF592**	**ZNF592**
**TEX261**	**TEX261**

SMYD1	SMYD1
GPR137	GPR137
ZNF74	ZNF74
WDFY3	WDFY3
INSR	INSR
SNX2	SNX2
RPL26	RPL26
IPO4	IPO4
BMP2	BMP2
KDELR1	KDELR1
MPI	MPI
FAM199X	FAM199X
DHX15	DHX15
ZC3H11A	ZC3H11A
RRM1	RRM1
KRT80	KRT80
RAB40C	RAB40C
AMOTL2	AMOTL2
POT1	POT1
TAX1BP1	TAX1BP1
PIAS4	PIAS4
ARL6IP4	ARL6IP4
FBXW2	FBXW2
BRD3	BRD3
POMK	POMK
MPL	MPL
DMPK	DMPK
FOXP2	FOXP2
PABPN1	PABPN1
FAM210A	FAM210A
SEC23IP	SEC23IP
CAPN15	CAPN15
LONRF2	LONRF2
ATP6V1C1	ATP6V1C1
PPP1R3B	PPP1R3B
UBE2M	UBE2M
FAM102A	FAM102A
C6orf201	C6orf201
SLC7A2	SLC7A2
FN1	FN1
STARD7	STARD7
DIMT1	DIMT1
USP7	USP7
KIAA0232	KIAA0232
AAK1	AAK1

PRKD3	PRKD3
R3HDM4	R3HDM4
HNRNPA2B1	HNRNPA2B1
BCOR	BCOR
ERGIC1	ERGIC1
STAT1	STAT1
VIMP	VIMP
PAPSS2	PAPSS2
IFITM2	IFITM2
AGPAT3	AGPAT3
DYRK1A	DYRK1A
XRCC5	XRCC5
PTEN	PTEN
HDHD2	HDHD2
MAPK1	MAPK1
SLAIN2	SLAIN2
MICAL3	MICAL3
ZNF561	ZNF561
TMPPE	TMPPE
GNG5	GNG5
RPL27A	RPL27A
GRB2	GRB2
CYB5R3	CYB5R3
BTG1	BTG1
SERTAD2	SERTAD2
SERBP1	SERBP1
SETD1A	SETD1A
OS9	OS9
RNF139	RNF139
RBM4	RBM4
RHOB	RHOB
NCKIPSD	NCKIPSD
JUP	JUP
CDK10	CDK10
DUSP1	DUSP1
SPOPL	SPOPL
BZW2	BZW2
GPS1	GPS1
SLC1A5	SLC1A5
ZFAND3	ZFAND3
USP10	USP10
MGAT4B	MGAT4B
COPS6	COPS6
SGK1	SGK1
AIG1	AIG1

**N4BP1**	**N4BP1**
**RPS29**	**RPS29**
**RTN3**	**RTN3**
**CAMTA2**	**CAMTA2**
**DSP**	**DSP**
**KDELR2**	**KDELR2**
**TXNIP**	**TXNIP**
**NR4A1**	**NR4A1**
**MARCKS**	**MARCKS**
**ZFP91**	**ZFP91**
**BRD4**	**BRD4**
