# Supplementary material for: Differential Expression of Host miRNAs During Ad14 and Ad14p1 Infection
Source: Viruses. 2025 Jun 11;17(6):838. doi: 10.3390/v17060838 (PMC12197781; doi:10.3390/v17060838)
Supplement: Supplementary file 1 [file viruses-17-00838-s001.zip › Supplementary Figures.pdf]

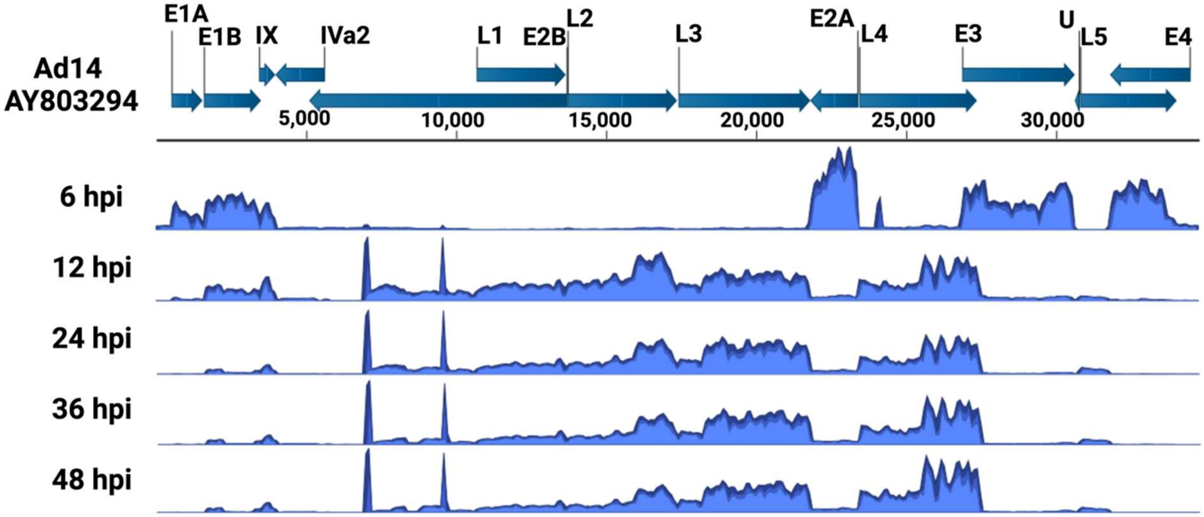

Figure S1. Mapping of total reads to Ad14 genome over time. Total reads mapping to the Ad14 genome from Ad14 infected A549 cells at 6, 12, 24, 26 and 48 hours post infection. Identical mapping with Ad14p1 infected cells was observed.

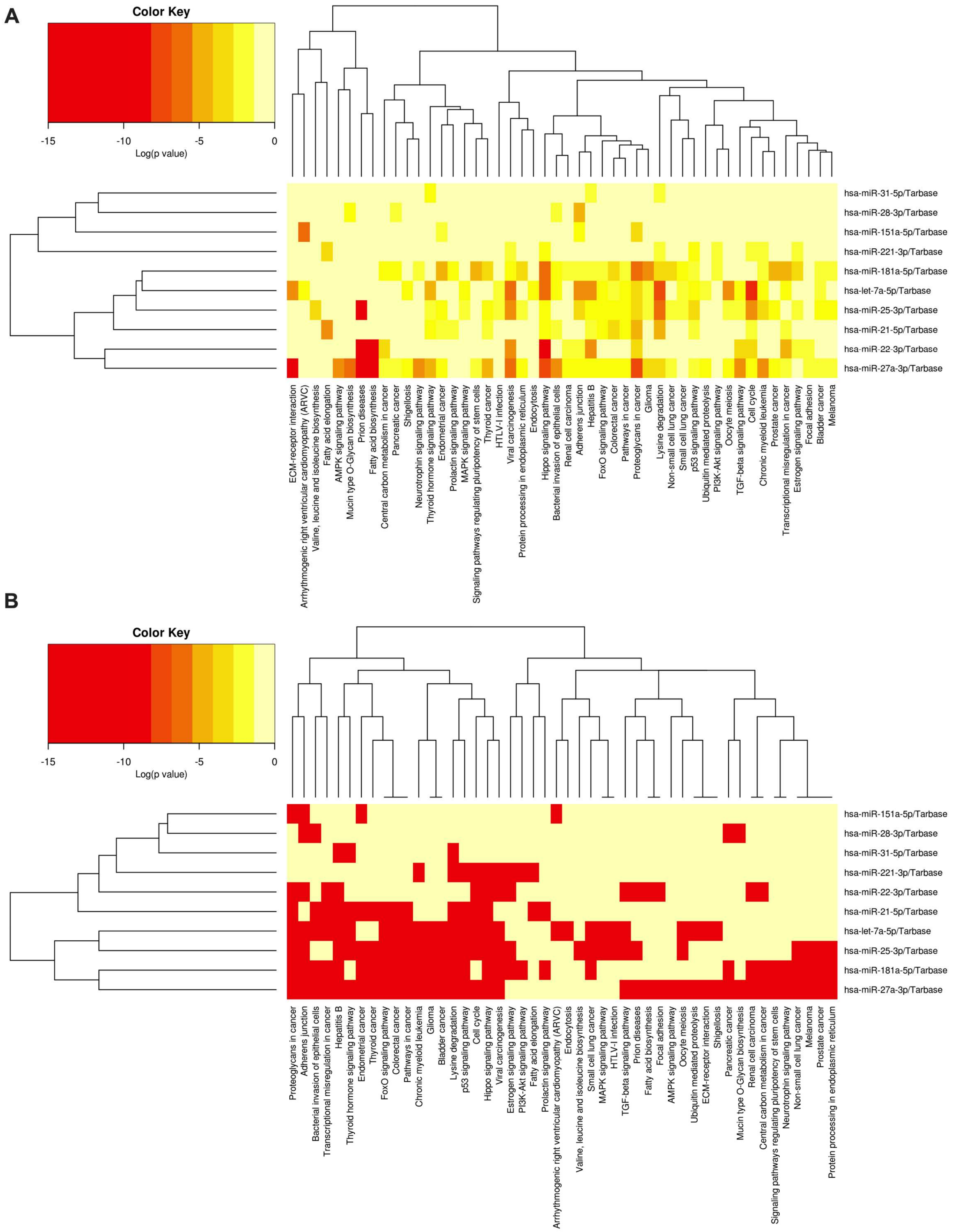
A
Color Key
-15
-10
-5
0
Log(p value)
hsa-miR-31-5p/Tarbase
hsa-miR-28-3p/Tarbase
hsa-miR-151a-5p/Tarbase
hsa-miR-221-3p/Tarbase
hsa-miR-181a-5p/Tarbase
hsa-let-7a-5p/Tarbase
hsa-miR-25-3p/Tarbase
hsa-miR-21-5p/Tarbase
hsa-miR-22-3p/Tarbase
hsa-miR-27a-3p/Tarbase
ECM-receptor interaction
Arrhythmogenic right ventricular cardiomyopathy (ARVC)
Valine, leucine and isoleucine biosynthesis
Fatty acid elongation
AMPK signaling pathway
Mucin type O-Glycan biosynthesis
Prion diseases
Fatty acid biosynthesis
Central carbon metabolism in cancer
Pancreatic cancer
Shigellosis
Neurotrophin signaling pathway
Thyroid hormone signaling pathway
Endometrial cancer
Prolactin signaling pathway
MAPK signaling pathway
Signaling pathways regulating pluripotency of stem cells
Thyroid cancer
HTLV-I infection
Viral carcinogenesis
Protein processing in endoplasmic reticulum
Endocytosis
Hippo signaling pathway
Bacterial invasion of epithelial cells
Renal cell carcinoma
Adherens junction
Hepatitis B
FoxO signaling pathway
Colorectal cancer
Pathways in cancer
Proteoglycans in cancer
Glioma
Lysine degradation
Non-small cell lung cancer
Small cell lung cancer
p53 signaling pathway
Ubiquitin mediated proteolysis
PI3K-Akt signaling pathway
Oocyte meiosis
TGF-beta signaling pathway
Cell cycle
Chronic myeloid leukemia
Prostate cancer
Transcriptional misregulation in cancer
Estrogen signaling pathway
Focal adhesion
Bladder cancer
Melanoma
B
Color Key
-15
-10
-5
0
Log(p value)
hsa-miR-151a-5p/Tarbase
hsa-miR-28-3p/Tarbase
hsa-miR-31-5p/Tarbase
hsa-miR-221-3p/Tarbase
hsa-miR-22-3p/Tarbase
hsa-miR-21-5p/Tarbase
hsa-let-7a-5p/Tarbase
hsa-miR-25-3p/Tarbase
hsa-miR-181a-5p/Tarbase
hsa-miR-27a-3p/Tarbase
Proteoglycans in cancer
Adherens junction
Bacterial invasion of epithelial cells
Transcriptional misregulation in cancer
Hepatitis B
Thyroid hormone signaling pathway
Endometrial cancer
Thyroid cancer
FoxO signaling pathway
Colorectal cancer
Pathways in cancer
Chronic myeloid leukemia
Glioma
Bladder cancer
Lysine degradation
p53 signaling pathway
Cell cycle
Hippo signaling pathway
Viral carcinogenesis
Estrogen signaling pathway
PI3K-Akt signaling pathway
Fatty acid elongation
Prolactin signaling pathway
Arrhythmogenic right ventricular cardiomyopathy (ARVC)
Endocytosis
Valine, leucine and isoleucine biosynthesis
Small cell lung cancer
MAPK signaling pathway
HTLV-I infection
TGF-beta signaling pathway
Prion diseases
Fatty acid biosynthesis
Focal adhesion
AMPK signaling pathway
Oocyte meiosis
Ubiquitin mediated proteolysis
ECM-receptor interaction
Shigellosis
Pancreatic cancer
Mucin type O-Glycan biosynthesis
Renal cell carcinoma
Central carbon metabolism in cancer
Signaling pathways regulating pluripotency of stem cells
Neurotrophin signaling pathway
Non-small cell lung cancer
Melanoma
Prostate cancer
Protein processing in endoplasmic reticulum

C

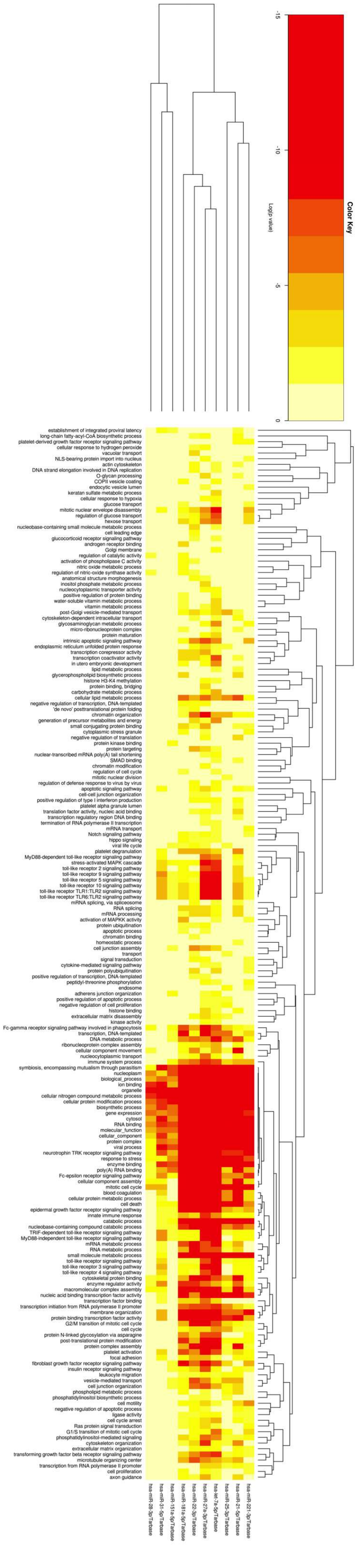

Figure S2. Heatmaps of KEGG and GO analysis of Ad14 miRNAs. The Ad14 miRNAs were uploaded to mirPath V3. **A-C.** Heatmaps of the KEGG Pathways Union (A), KEGG Genes Union (B) and GO Categories Union (C) analysis.

A.

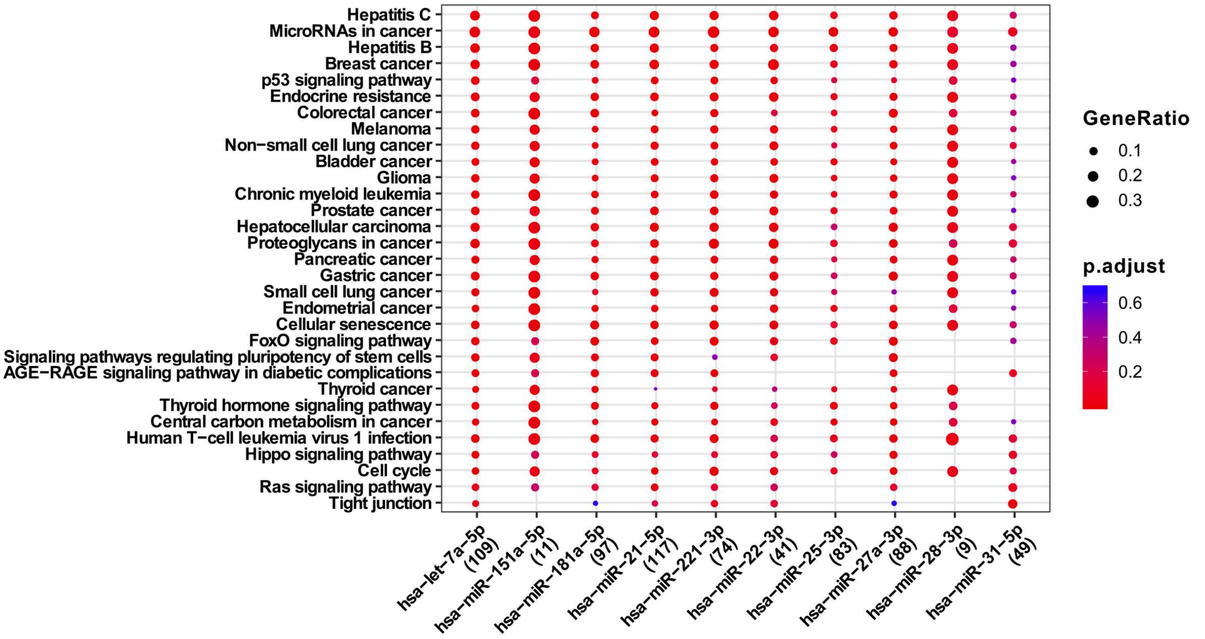

B.

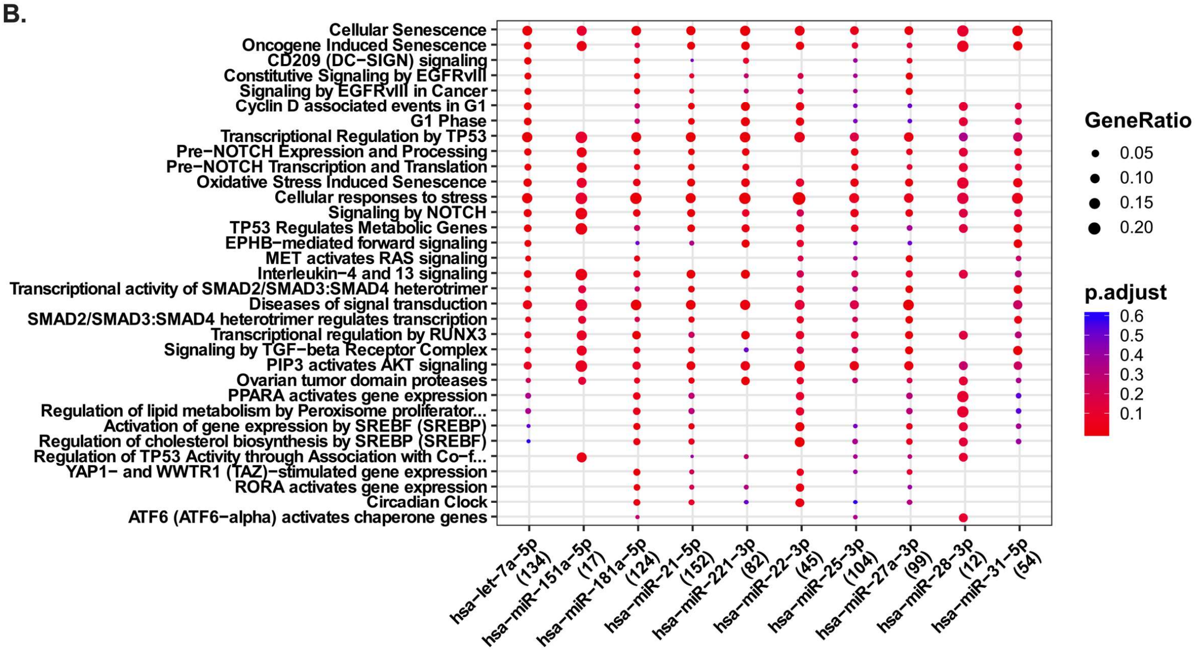

Figure S3. Dotplots of KEGG and Reactome functional enrichment of Ad14 miRNAs. A&B. The 10 enriched miRNAs in Ad14 CPE corpses were uploaded to MIENTURNET. Targent enrichment was performed with miRTarBase with a minimum of 2 miRNA-RNA interactions and an FDR threshold of 1. Dotplots of KEGG (A) and Reactome (B) functional enrichment. Number of genes targeted by each miRNA are shown in parentheses.

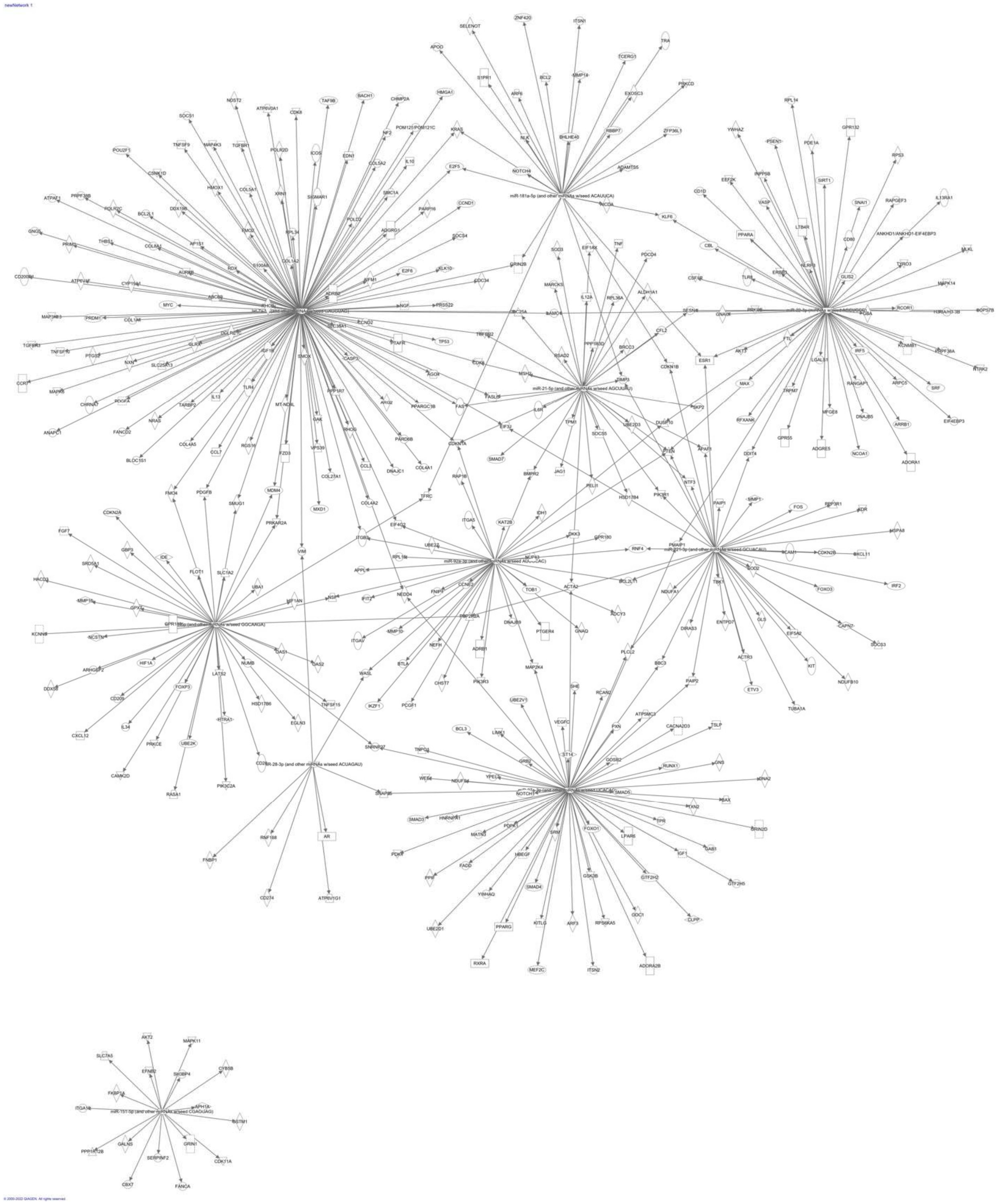

Figure S4. IPA network analysis of Ad14 miRNA and predicted targets. IPA microRNA filter analysis was performed on the Ad14 miRNA. Network analysis of miRNA and targets filtered for cell type (macrophages), pathways (cellular stress & injury, cytokine signaling, disease specific

pathways and pathogen-influenced signaling) and confidence level (experimental observed and high predicted).

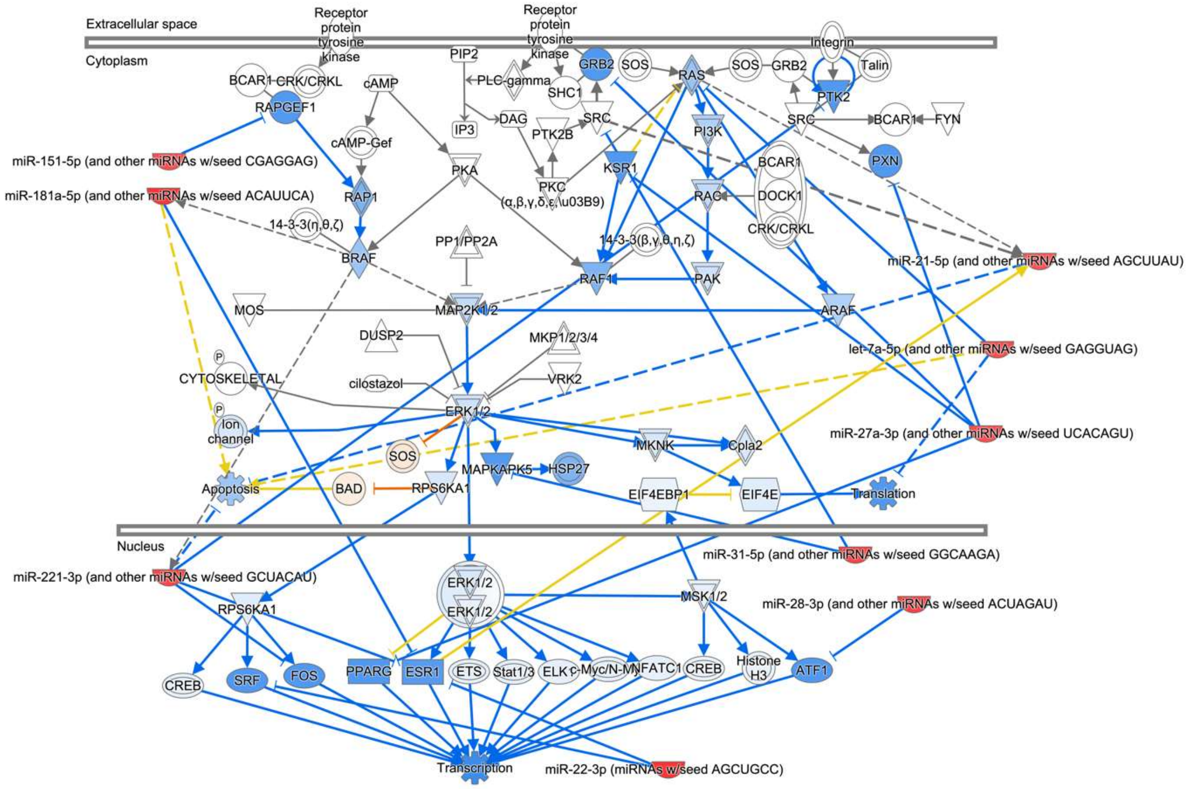

© 2000-2022 QIAGEN. All rights reserved.

Figure S5. Effect of Ad14 miRNAs on ERK signaling pathway. IPA was used to predict the effects of Ad14 miRNAs on proteins involved in the ERK signaling pathway. Lines ending with an arrowhead show the direction of activation, while lines ending with a dash show the direction of inhibition. Solid blue lines indicate validated repressive effects of miRNA-target interactions, while dashed blue lines are for predicted repressive effects of miRNA-target interactions. Red cresents are miRNAs, and the remaining shapes are proteins in the ERK pathway. Dark blue shapes are direct targets of the Ad14 miRNAs, while light blue shapes are predicted to have decreased activation based on repression of an upstream activator.

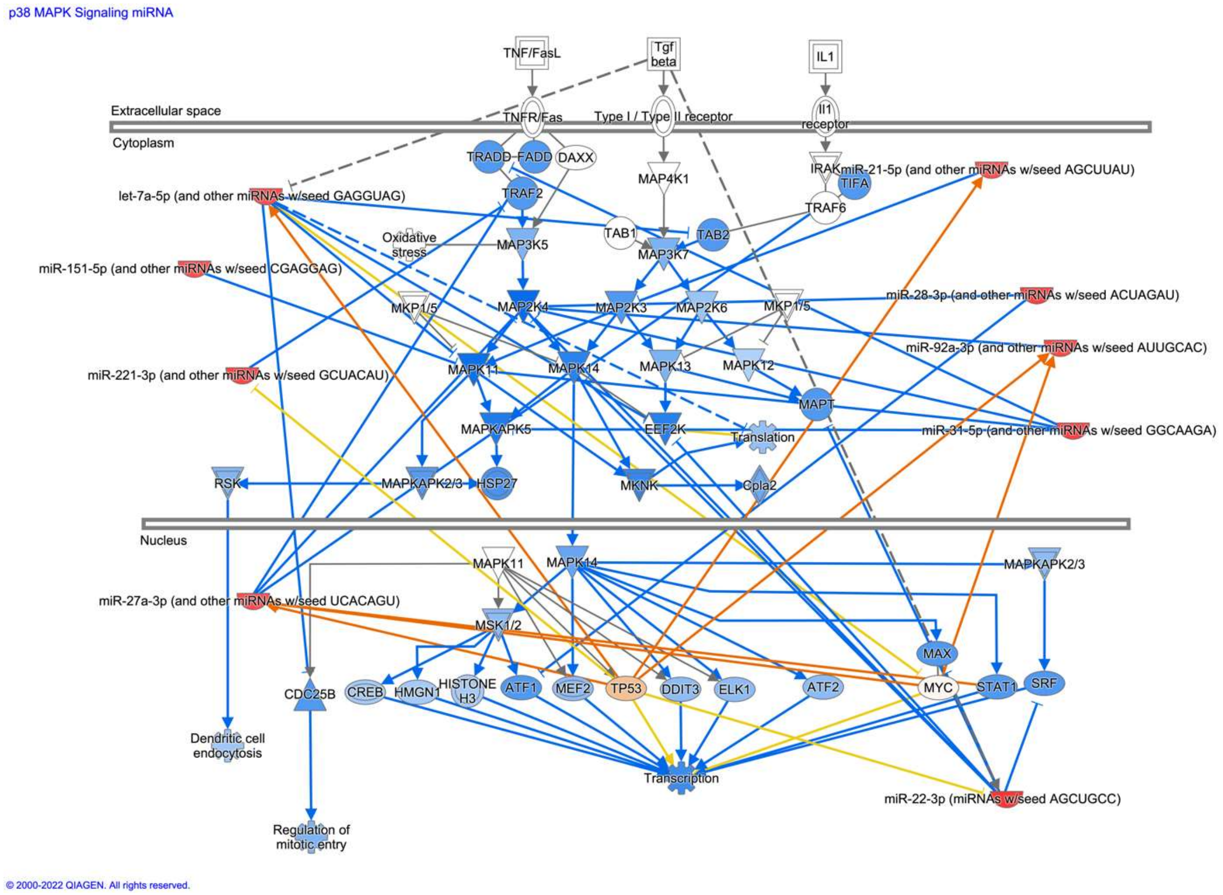

© 2000-2022 QIAGEN. All rights reserved.

Figure S6. Effect of Ad14 miRNAs on p38 MAPK signaling pathway. IPA was used to predict the effects of Ad14 miRNAs on proteins involved in p38 MAPK signaling. Lines ending with an arrowhead show the direction of activation, while lines ending with a dash show the direction of inhibition. Solid blue lines indicate validated repressive effects of miRNA-target interactions, while dashed blue lines are for predicted repressive effects of miRNA-target interactions. Red cresents are miRNAs, and the remaining shapes are proteins in the p38 MAPK pathway. Dark blue shapes are direct targets of the Ad14 miRNAs, while light blue shapes are predicted to have decreased activation based on repression of an upstream activator.

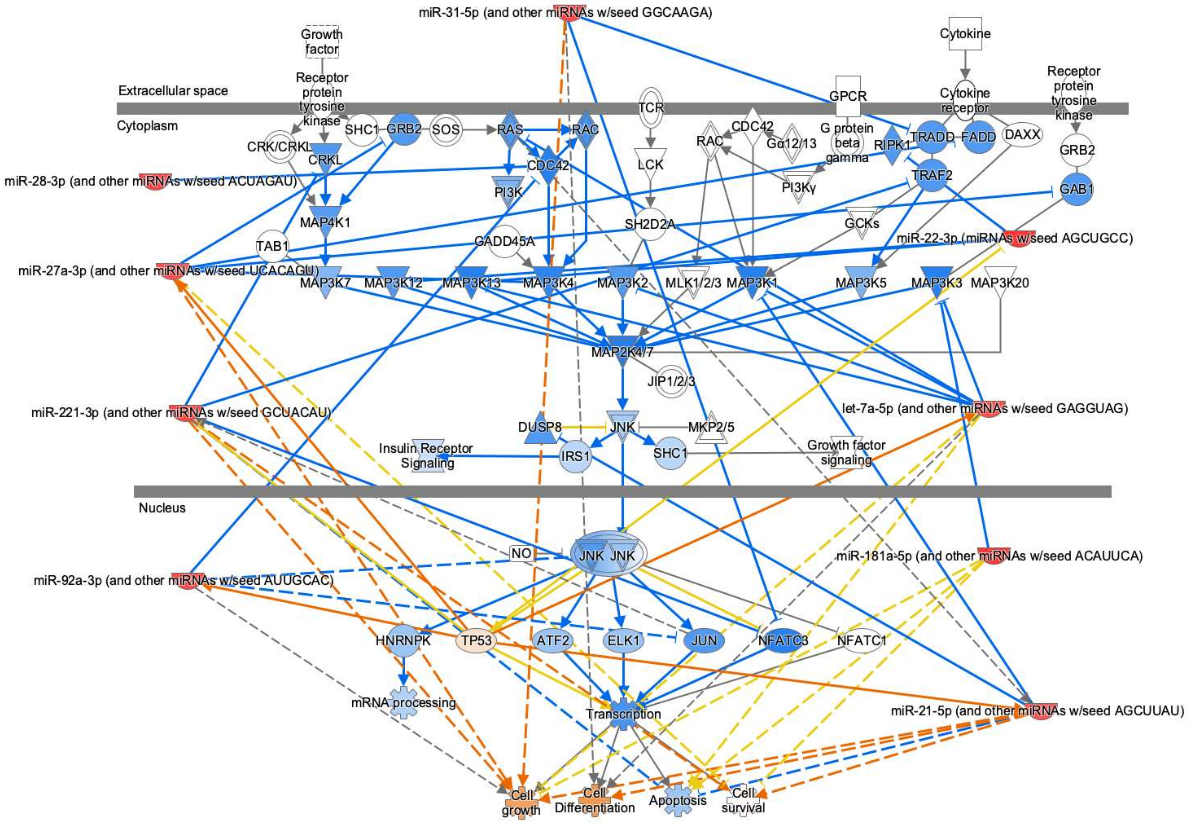

© 2000-2022 QIAGEN. All rights reserved.

Figure S7. Effect of Ad14 miRNAs on JNK signaling pathway. IPA was used to predict the effects of Ad14 miRNAs on proteins involved in JNK signaling. Lines ending with an arrowhead show the direction of activation, while lines ending with a dash show the direction of inhibition. Solid blue lines indicate validated repressive effects of miRNA-target interactions, while dashed blue lines are for predicted repressive effects of miRNA-target interactions. Red cresents are miRNAs, and the remaining shapes are proteins in the JNK pathway. Dark blue shapes are direct targets of the Ad14 miRNAs, while light blue shapes are predicted to have decreased activation based on repression of an upstream activator.
